# MiR-23a Facilitates the Replication of HSV-1 through the Suppression of Interferon Regulatory Factor 1

**DOI:** 10.1371/journal.pone.0114021

**Published:** 2014-12-02

**Authors:** Jing Ru, Huahui Sun, Hongxia Fan, Chunmei Wang, Yixuan Li, Min Liu, Hua Tang

**Affiliations:** 1 Tianjin Life Science Research Center and Department of Microbiology, School of Basic Medical Sciences, Tianjin Medical University, Tianjin 300070, China; 2 Department of Pathophysiology, School of Basic Medical Sciences, Yunnan University of Traditional Chinese Medicine, Kunming 650500, China; Institut Pasteur of Shanghai, Chinese Academy of Sciences, China

## Abstract

MicroRNAs (miRNAs) are small, non-coding RNAs that negatively regulate gene expression. It has been reported that miRNAs are involved in host-virus interaction, but evidence that cellular miRNAs promote virus replication has been limited. Here, we found that miR-23a promoted the replication of human herpes simplex virus type 1 (HSV-1) in HeLa cells, as demonstrated by a plaque-formation assay and quantitative real-time PCR. Furthermore, interferon regulatory factor 1 (IRF1), an innate antiviral molecule, is targeted by miR-23a to facilitate viral replication. MiR-23a binds to the 3′UTR of IRF1 and down-regulates its expression. Suppression of IRF1 expression reduced RSAD2 gene expression, augmenting HSV-1 replication. Ectopic expression of IRF1 abrogated the promotion of HSV-1 replication induced by miR-23a. Notably, IRF1 contributes to innate antiviral immunity by binding to IRF-response elements to regulate the expression of interferon-stimulated genes (ISGs) and apoptosis, revealing a complex interaction between miR-23a and HSV-1. MiR-23a thus contributes to HSV-1 replication through the regulation of the IRF1-mediated antiviral signal pathway, which suggests that miR-23a may represent a promising target for antiviral treatments.

## Introduction

MicroRNAs (miRNAs) are small, ∼22-nucleotides, RNA molecules that were first discovered in *Caenorhabditis elegans* and are expressed in a wide range of eukaryotic organisms [1], [2]. Mammalian miRNAs can bind to imperfectly complementary sites in the 3′ noncoding regions (3′UTRs) of target mRNAs and thereby act as specific post-transcriptional inhibitors of mRNA function [3]. The gene-silencing effect triggered by miRNAs may serve major function at two levels to modulate host–virus interactions [4]–[6]. On the one hand, cellular miRNAs target viral mRNAs in the defense against viral infection [7]. Secondly, several viral miRNAs regulate the expression of cellular factors that are involved in cellular innate responses that down-regulate the expression of key viral proteins [8], [9].

HSV-1 is an alpha herpesvirus that most commonly causes localized mucocutaneous lesions but can also cause meningitis and encephalitis [10]. The global prevalence of HSV-1 is approximately 90%. HSV-1 can establish lifelong persistent infection (latency). In response to a variety of stimuli, the virus can periodically reactivate to resume replication. The interactions of HSV-1 and its host cells, including miRNA regulation, contribute to the establishment of HSV-1 infection [11]. For example, HSV-1 uses viral miRNAs to down-regulate the immediate-early transactivators ICP0 and ICP4 in latently infected cells, most likely stabilizing the latent state [12]. Additionally, herpes simplex virus IE63 (ICP27) protein interacts with spliceosome-associated protein 145 and inhibits splicing to inhibit pre-mRNA processing during HSV-1 infections [13]. However, few studies focus on the regulation of cellular miRNAs [14].

MiR-23a is thought to have oncogenic effects via the modulation of cell proliferation, survival, and apoptosis during the initiation and progression of human cancers [15]–[17]. Dysregulation of miR-23a has been found in various human cancers, including tumors occurring in the breast, colon, and lung; gastric cancers; hepatocellular carcinoma; and acute myeloid leukemia [18]–[23]. miR-23a regulates cell functions through modulation of target genes, such as transcription factor HOXB4 and metallothionein 2A [24], [25]. Recently, interferon regulatory factor 1 (IRF1), which is involved in innate antiviral immunity, inflammation, and the pro-apoptotic pathway, was identified as a target of miR-23a to regulate cells growth and apoptosis in gastric adenocarcinoma [26]. We hypothesized that miR-23a may modulate viral-host interaction through IRF1. In this study, we found that miR-23a modulated the IRF1-mediated pathway to facilitate HSV-1 replication in HeLa cells, revealing that miRNAs play an important role in virus-host interaction during viral infection.

## Materials and Methods

### Cell culture

HeLa cells were cultured in RPMI 1640 medium (GIBCO BRL, Grand Island, NY, USA) supplemented with 10% fetal bovine serum (FBS), 100 U/ml penicillin and 100 µg/ml streptomycin at 37°C under 5% CO_2_.

### Virus preparation

The HSV-1 Stocker strain (wild type) was obtained from Chinese Center For Disease Control And Prevention and was propagated in the HeLa cells. At the peak of cytopathogenic effect (CPE), viruses were harvested by fast freezing and slow thawing for three cycles. At low centrifugation force (5500×g) for 5 min, the supernatant was aliquoted and stored at −80°C.

### Plasmids construction

To express miR-23a, we amplified a DNA fragment containing the pri-miR-23a from genomic DNA using the following PCR primers: miR-23a-S, 5′– GCGGTACCTGGCTCCTGCATATGAG – 3′, miR-23a-AS: 5′ – GATGAATTCCAGGCACAGGCTTCGG – 3′, the amplified fragment was then inserted into pcDNA3 between the KpnI and EcoRI sites.

Anti-miR-23a plasmid expressing miR-23a antisense was constructed by inserting annealed double strand oligogmers of miR-23a-sense-Top(GATCCGGAAATCCCTGGCAATGTGATTTTTTC) and miR-23a-antisense-Bot (TCGAGAAAAAATCACATTGCCAGGGATTTCCG) into BamHI and XhoI sites of pRNAT-U6.2/Lenti. The specificity of the anti-miR-23a has been validated in our previous study [21], [26].

The full-length human RSAD2 gene was amplified by PCR using specific primers (RSAD2-S: 5′ CGAGAATTCGCCACCATGTGGGTGCTTACAC 3′; RSAD2-AS: 5′ CATAGCTCGAGACCAATCCAGCTTCAGATCAG 3′) from cDNA and cloned into pcDNA3 at EcoRI and XhoI sites. The triple Myc tags were placed at the 3′ terminal of RSAD2 gene to generate C-terminal Myc-tagged RSAD2 proteins. The recombinant plasmid was designated as pcDNA3/Myc-RSAD2.

Other plasmids presented in this report were generated by previous vector-construct work in our lab, including pcDNA3/IRF1 and pSilencer/sh-IRF1.

### Transient transfection and HSV-1 infection of HeLa cells

Transient transfection was performed using the Lipofectamine 2000 reagent (Invitrogen, Carlsbad, California, USA), according to the manufacturer's specifications.

HeLa cells seeded on 48-well plate were transfected with experimental plasmids and controls. At 24 h post-transfection, the plate was incubated with HSV-1 at a multiplicity of infection (MOI) of 0.01, until the peak of CPE the viruses were harvested by freezing and thawing for three cycles. Virus titers in the supernatants and cells were determined by standard plaque assay [27]. To visualize plaques, neutral red staining was used as described previously [28]. Briefly, monolayers of HeLa cells were infected with serial dilutions of the above harvested virus for 90 min, then the virus suspensions were removed and cells were overlaid with RPMI 1640 containing 1.6% methylcellulose to allow virus only spread via cell to cell route. After 48–72 h post-infection, the number of plaques in each well was counted under the microscope. To measure the plaque areas, the plates were stained with neutral red for 6 h and examined under the microscope.

### Fluorescent report assay

HeLa cells were transfected with 0.2 µg of the fluorescent reporter vector with 0.2 µg of the miR-23a expression vector or the inhibitor and controls. The vector pDsRed2-N1 (Clontech, Mountain View, CA), expressing red fluorescent protein (RFP), was spiked in and used for normalization. At 48 h post-transfection, cells were lysed with RIPA lysis buffer (0.15 M NaCl, 0.05 M Tris-HCl pH 8.0, 1% Triton X-100, 0.1% SDS). The fluorescence intensities of EGFP and RFP were measured using an F-4500 Fluorescence Spectrophotometer (Hitachi, Tokyo, Japan), according to the manufacturer's protocol.

### MTT Assay

HeLa cells were seeded on 48-well plates at 4000 cells per well and transfected with pcDNA3/miR-23a or pcDNA3/IRF1 and controls. At 24 h post-transfection, the cells were transferred to 96-well plates and the MTT (3-(4, 5-dimethylthiazol-2-yl)-2, 5-diphenyl-tetrazolium bromide) assays were performed to assess cell viability. The absorbance at 570 nm was measured using a μQuant Universal Microplate Spectrophotometer (BioTek, Winooski, VT).

### Real-time PCR

To quantify the level of gene expression, 1 µl of cDNA was used as the template in each 20-µl reactionwith SYBR Premix ExTaq (TakaRa, Otsu, Shiga, Japan), the specific primer pairs were designed as follows: miR-23a forward: 5′ – TGCGGATCACATTGCCAGG – 3′; miR-23a reverse, 5′-CCAGTGCAGGGTCCGAGGT-3′;RSAD2-qPCR-S: 5′ CTGTCCGCTGGAAAGTG 3′; RSAD2-qPCR-AS: 5′ GCTTCTTCTACACCAACATCC 3′. Amplification was carried out in an iQ5 Real-Time PCR system (Bio-rad) as follows: 94°C for 3 min, followed by 40 cycles of 94°C for 30 s, 56°C for 30 s and 72°C for 30 s. 18S rRNA was used for normalization [29], and U6 was used as the internal control gene to detect the relative level of miRNA [30]. The quantitative real-time PCR results were analyzed and expressed as relative CT (cycle threshold) values [31].

To quantify the HSV-1 copies, extracted DNA was used as the template for quantitative real-time PCR, and the glycoprotein D (gD) gene of HSV-1 was amplified using specific primers [32].

### Western blot analysis

Transfection of HeLa cells and infection of HSV-1 were performed as described above. Cell lysates were obtained with RIPA lysis buffer, and proteins were separated on a 10% polyacrylamide-SDS gel. Following protein transfer to nitrocellulose membranes, the level of IRF1 expression was visualized by blotting with anti-IRF1 (Saier Biotech, Tianjin, China) and anti-FALG (Cell Signaling Technology, Beverly MA). The level of RSAD2 expression was visualized by blotting with anti-RSAD2 (ProteinTech Group, Chicago, IL, USA). The loading control, GAPDH, was evaluated using anti-GAPDH (Saier Biotech, Tianjin, China).

### Immunofluorescence assay

HeLa cells transfected with the relative vectors were infected with HSV-1 at 0.01 PFU/cell. At 24 h post-infection, cells were fixed with 4% paraformaldehyde in PBS at room temperature for 20 min and permeabilized for 5 min with 0.5% (v/v) Triton X-100. Samples were washed three times with 1×PBS and blocked for 30 min in 1×PBS containing 10% (w/v) donkey serum. Then the primary anti-HSV-1-glycoprotein monoclonal antibody (Saier Biotech, Tianjin, China) was added. Secondary antibody against mouse IgG was directly conjugated to FITC (Invitrogen, Carlsbad, CA). After the final wash, samples were counterstained with DAPI (4, 6-diamidino-2-phenylindole, Dojindo Molecular Technologies, Inc., Japan) to visualize nuclei with an imaging system (NIS Elements F 2.20 imaging software, Nikon, Tokyo, Japan).

### Statistical analysis

Statistics are reported as the mean and the standard error of the mean for each group, and the data are presented as the probability and test used for analysis. (***p<0.001, **p<0.01, *p<0.05, *ns* = not significant)

## Results

### miR-23a facilitates HSV-1 replication in HeLa cells

**Figure 1:**
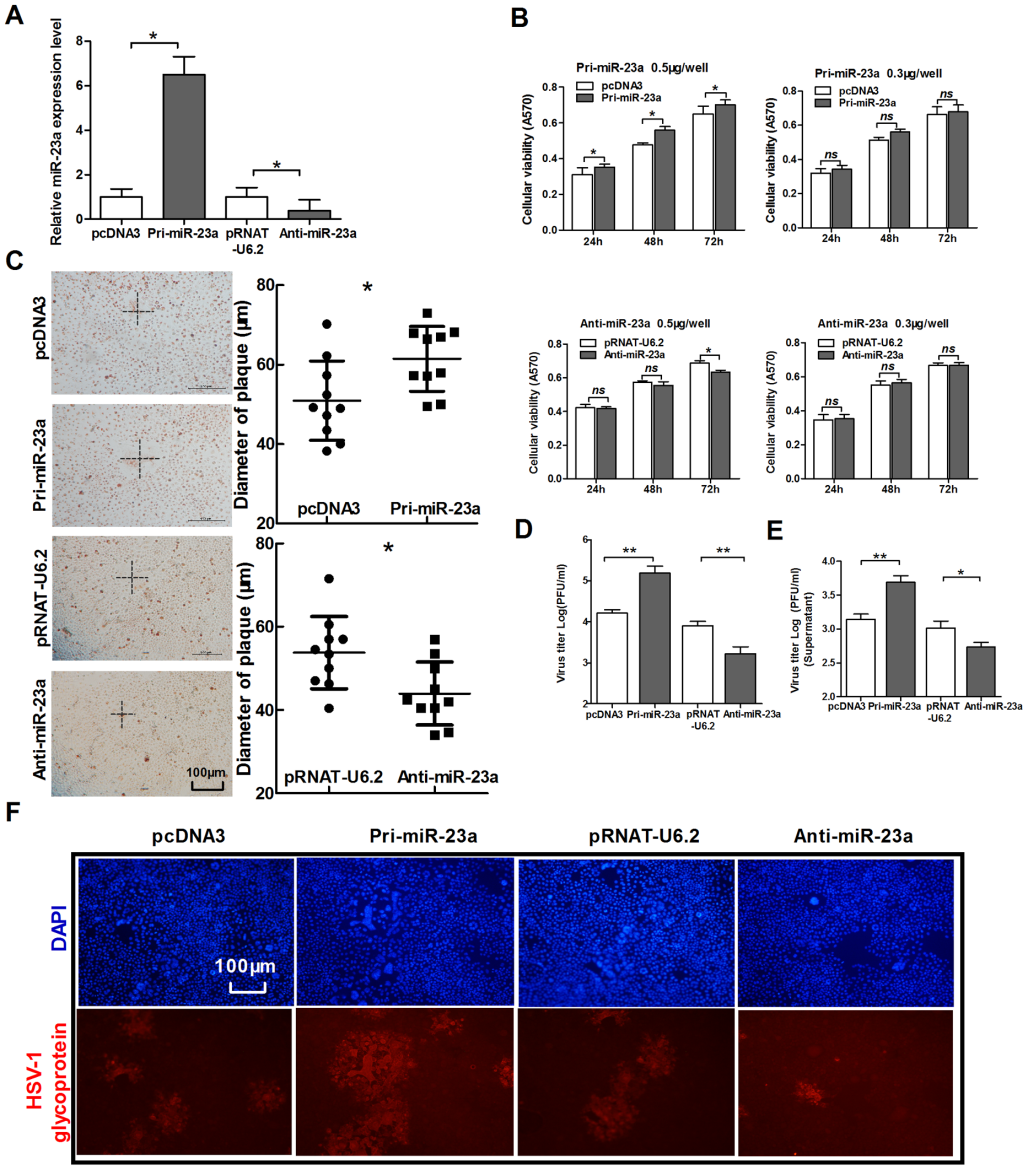
MiR-23a promotes the replication of HSV-1. (A) HeLa cells were transfected with Pri-miR-23a, pcDNA3, Anti-miR-23a and pRNAT-U6.2, respectively. At 24 h post-transfection, total RNA was extracted and analyzed for miR-23a expression by quantitative real-time PCR. (B) HeLa cells were transfected as indicated in (A). Cell viability was measured by MTT assay at 24 h, 48 h and 72 h post-transfection. To up-regulate miR-23a, two doses of vectors were used for transfection, 0.5 µg/well and 0.3 µg/well. Another group was transfected with Anti-miR-23a and its control vector in the same way. (C–F) HeLa cells were transfected as indicated in (A), 24 h post-transfection, cells were infected with HSV-1 at 0.01 PFU/cell. At 48 h post-infection, the radius of the cytopathic area was measured by neutral red staining. The scale bar represents 100 µm (C). Total viral yields (D) and yield of progeny virions from the culture supernatant (E) were determined by standard plaque assays. Level of glycoprotein expression was determined by immunofluorescence assay (F). All data represent the mean value ± SD of at least three independent experiments. *: p<0.05; **: p<0.01; ***: p<0.001; *ns*: No significant differences by Student's t test.

HeLa cells were transfected with pcDNA3, Pri-miR-23a, pRNAT-U6.2 or Anti-miR-23a. PCR was performed to validate the efficiency of the constructed plasmids (Fig. 1A). To exert the least influence on cell viability with abnormal expression of miR-23a, we first examined the viability of HeLa cells transfected with different doses of miR-23a expression vector (Pri-miR-23a) or anti-miR-23a expression vector (Anti-miR-23a) by MTT assay. As shown in Fig. 1B, HeLa cells transfected with Pri-miR-23a or Anti-miR-23a at 0.3 µg per well/48-well plate showed no obvious change in viability. Thus, this transfection procedure was used in future experiments.

To examine the strength of CPE induced by HSV-1, we first performed plaque-formation assays and measured the size (radius) of plaques through neutral-red staining. Over-expression of miR-23a by transiently transfected with Pri-miR-23a resulted in larger plaques compared to the control vector. Conversely, blocking miR-23a produced smaller plaques compared to the control vector (Fig. 1C). Then, we analyzed the concentration of viral progeny by a standard plaque assay. The results indicated that expression of miR-23a promoted viral replication, whereas down-regulation of miR-23a reduced or completely reversed the pro-virus effect (Fig. 1D). To further verify the role of miR-23a in virus replication, we quantified the concentration of infectious viruses in the supernatant. As in viral titers in cell, over-expression miR-23a showed the most viral replication (Fig. 1E). Thus, miR-23a may promote infection at the cellular level. miR-23a also increased the number of cells infected, as shown by the level of fluorescence, while blocking miR-23a had the opposite effect (Fig. 1F). These data indicate that miR-23a facilitates HSV-1 replication in host cells.

### miR-23a targets IRF1 directly and negatively regulates IRF1 expression in HeLa cells

To illustrate the possible mechanism underlying the above effect, it is necessary to identify the target genes of miR-23a. Our laboratory previously demonstrated that miR-23a directly targets IRF1 genes and negatively regulates their expression in gastric adenocarcinoma cells [26].

**Figure 2:**
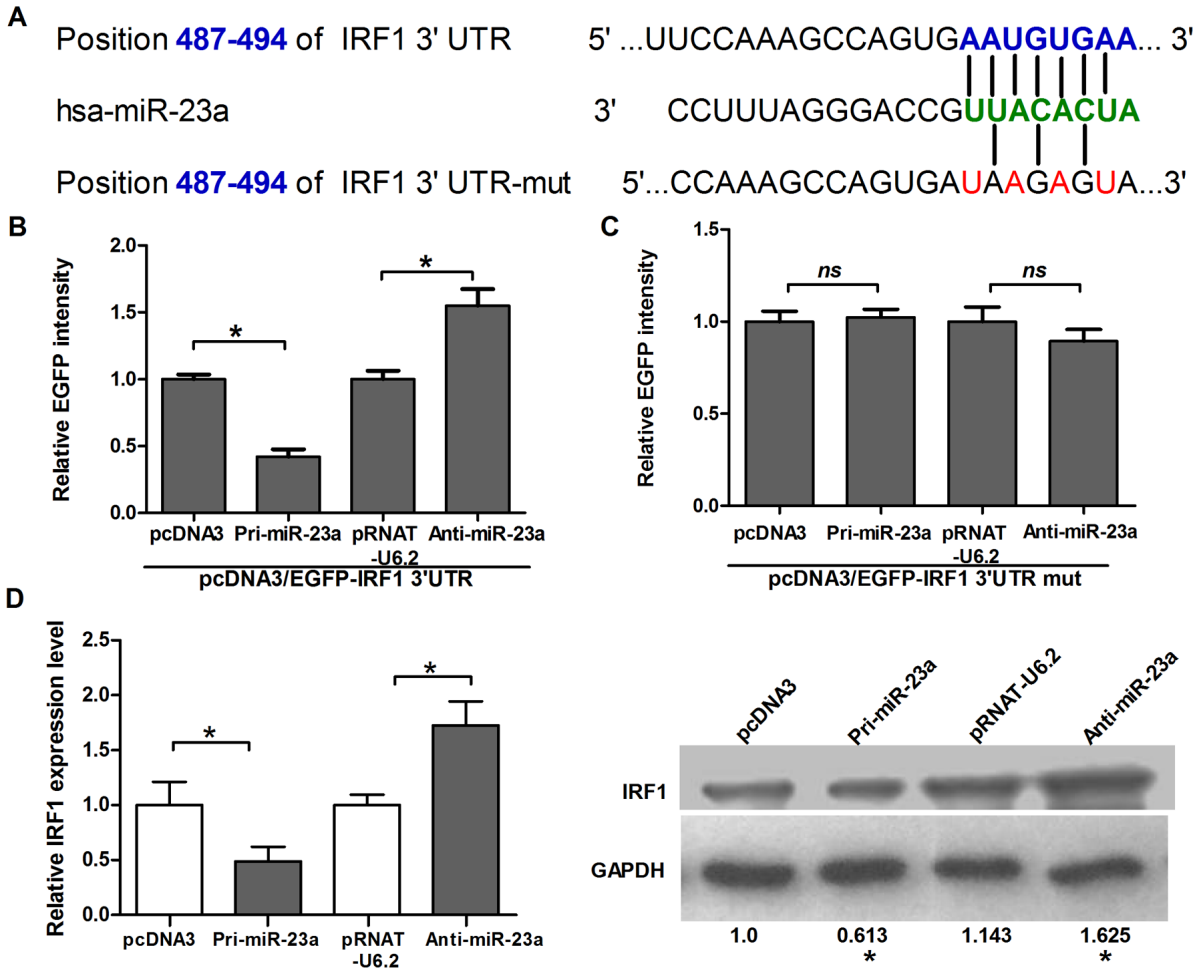
IRF1 is the direct target of miR-23a. (A) As predicted in the TargetScan database, the IRF1 3′UTR carries a miR-23a-binding site. The IRF1 3′UTR mutant, containing four mutated nucleotides within the miR-23a-binding site, is shown. The mutated nucleotides are marked in red. (B) HeLa cells were transfected with pcDNA3, Pri-miR-23a, pRNAT-U6.2 and Anti-miR-23a respectively, and co-transfected with pcDNA3/EGFP-IRF1-UTR reporter vector or pcDNA3/EGFP-IRF1-MUT mutant vector, as indicated. At 48 h post-transfection, the cell lysate was prepared to measure the EGFP intensity and the fluorescence value in the control group was set to 1. (C) HeLa cells were transfected with Pri-miR-23a, pcDNA3, pRNAT-U6.2 and Anti-miR-23a, respectively. At 48 h post-transfection, RNA was extracted from transfected HeLa cells and the IRF1 mRNA was quantified by real-time PCR. GAPDH mRNA was used as an internal control, and the group control was the level of IRF1 mRNA. The protein level of IRF1 was measured by Western blot. All data represent the mean value ± SD of at least three independent experiments. *p<0.05.

To confirm that miR-23a directly binds the IRF1 3′ UTR and regulates gene expression in HeLa cells, either a pcDNA3 vector expressing EGFP and carrying the 3′ UTR of IRF1 containing the predicted miR-23a-binding sites downstream or a control vector containing the mutational sites (Fig. 2A) was co-transfected with a vector expressing pre-miR-23a. As shown in Fig. 2B, the intensity of EGFP fluorescence in cells transfected with the wide type reporter vector was lower compared to the control group at 48 h post-transfection, suggesting that miR-23a may target IRF1 and specifically suppress its expression by binding to 3′ UTR. Conversely, knockdown of miR-23a by anti-miR-23a enhanced EGFP expression (Fig. 2B). However, when the miR-23a binding site in the EGFP-IRF1 3′ UTR reporter vector was mutated (EGFP-IRF1 3′ UTR mutant), neither overexpression nor blocking of miR-23a affect the intensity of EGFP fluorescence (Fig. 2C). The data from the real-time PCR and Western blot analysis further supported this inverse correlation (Fig. 2D).

### IRF1 gene confers an antiviral state to HeLa cells infected with HSV-1

**Figure 3:**
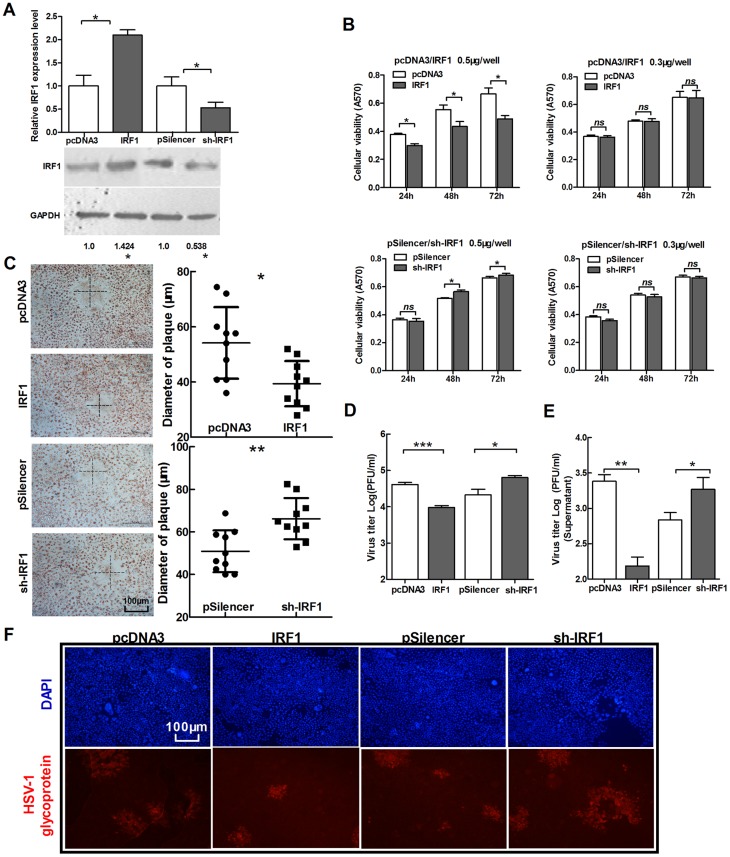
IRF1 suppresses the replication of HSV-1. (A) HeLa cells were transfected with IRF1, sh-IRF1 and control vectors, respectively. Total RNA was extracted and analyzed for IRF1 mRNA by quantitative real-time PCR. The cell lysate was extracted and analyzed for IRF1 expression by Western blot. (B) HeLa cells were transfected as indicated in (A), MTT assay of cell viability was conducted at 24 h, 48 h and 72 h post-transfection. To up-regulate IRF1, two doses of vectors were used for transfection, 0.5 µg/well and 0.3 µg/well. Another group was transfected with sh-IRF1 and its control vector in the same way. (C–F) HeLa cells were transfected as indicated in (A), 24 h post-transfection, cells were infected with HSV-1 at 0.01 PFU/cell. At 48 h post-infection, cells were stained with neutral red. The mean radius of the cytopathic area was measured. The scale bar represents 100 µm (C). Total viral yields (D) and Yield of progeny virions from the culture supernatant (E) were determined by standard plaque assays. Level of glycoprotein expression was determined by immunofluorescence assay (F). All data represent the mean value ± SD of at least three independent experiments. *: p<0.05; **: p<0.01; ***: p<0.001; *ns*: No significant differences by Student's t test.

Like miR-23a, IRF1 also possesses essential functions in modulating cell growth and apoptosis [33], [34]. First we confirmed the efficiency of plasmids IRF1 and sh-IRF1 (Fig. 3A). Indicating by MTT assay, 0.3 µg per well/48-well plate was indicated as an appropriate dose for transfection to observe no obvious effect on cell viability (Fig. 3B). And next, expression of IRF1 suppressed HSV-1 replication in HeLa cells, while opposite response was observed in cells transfected with knock-down-IRF expression vector (sh-IRF1) (Fig. 3C, D, E and F).

### Ectopic expression of IRF1 counteracts the viral replication induced by miR-23a

**Figure 4:**
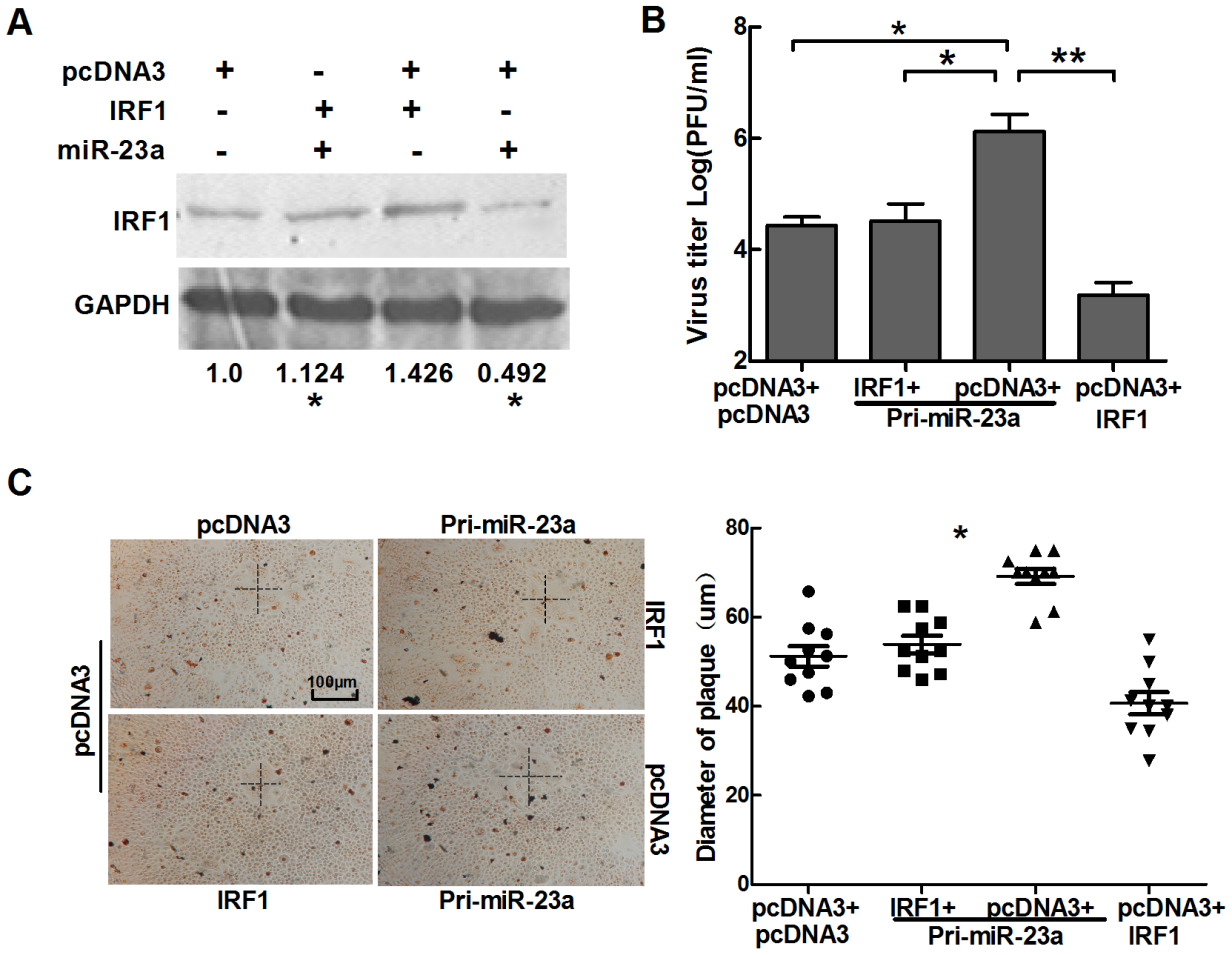
Transfection with IRF1 cDNA lacking a 3′UTR counteracts the effects of miR-23a on HSV-1 replication. (A) HeLa cells were co-transfected with two of pcDNA3, Pri-miR-23a and IRF1. At 72 h post-transfection, Western blot was used to detect the expression level of IRF1. (B) and (C), HeLa cells were transfected with either IRF1 or control vector, along with Pri-miR-23a or control vector, as indicated. At 24 h post-transfection, cells were infected with HSV-1 at 0.01 PFU/cell. Plaques were stained with neutral red, and viral yields were determined by standard plaque assays. Scale bar represents 100 µm. All data represent the mean value ± SD of at least three independent experiments. * p<0.05.

As miR-23a directly targets the 3′ UTR of IRF1 and down-regulates its expression, an expression vector containing only the open reading frame (ORF) of IRF1 should rescue the enhancement of viral replication induced by ectopic expression of miR-23a. Western-blot assay showed that IRF1 expression was significantly increased in HeLa cells co-transfected with IRF1 and miR-23a compared to those transfected with miR-23a and pcDNA3 (Fig. 4 A). As expected, similar results were found in viral titers and neutral-red staining (Fig. 4B, C). These data further confirm that miR-23a and IRF1 are inversely correlated not only in regulation but also in function.

### Endogenous miR-23a and IRF1 levels are affected by HSV-1 infection

**Figure 5:**
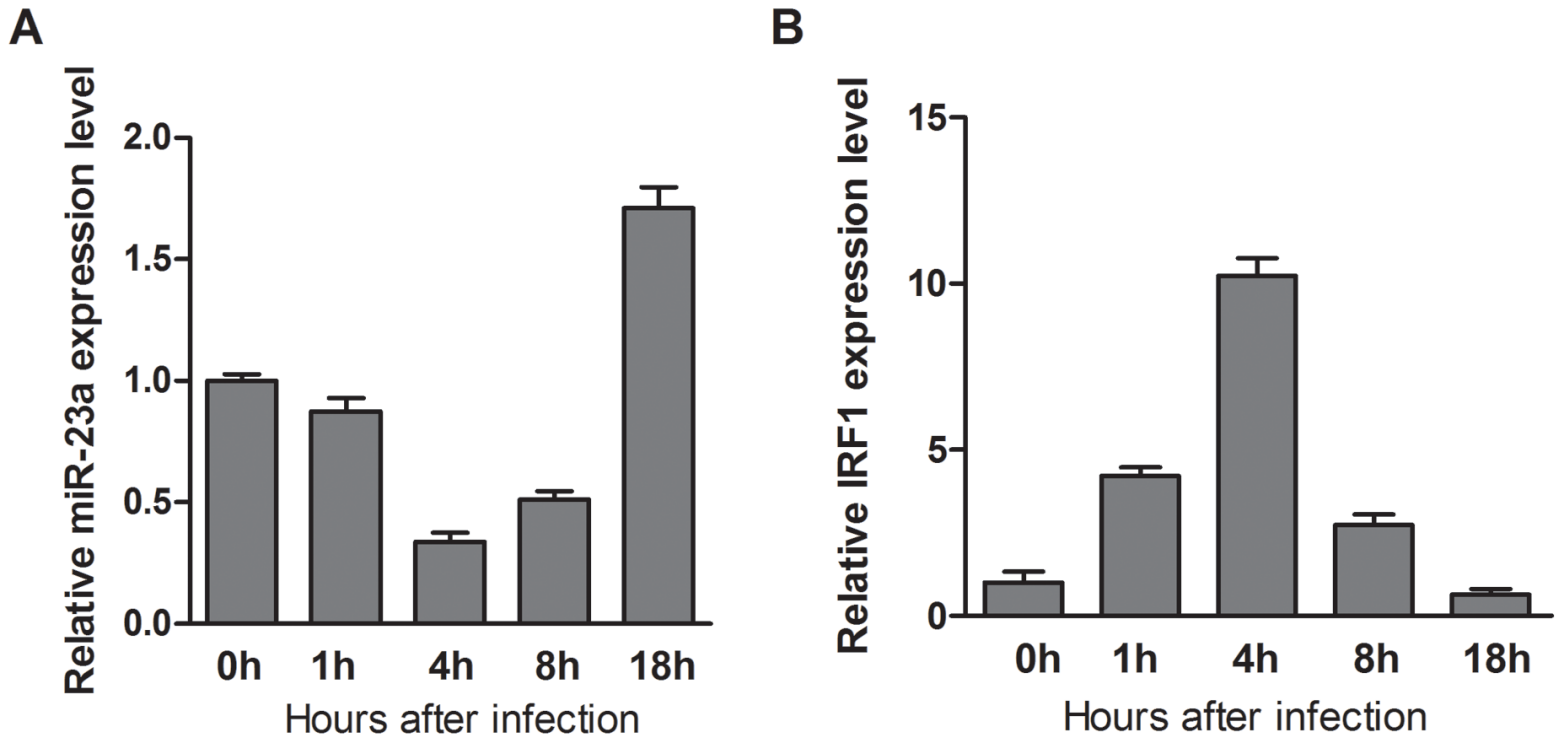
Time course of miR-23a and IRF1 in HeLa cells infected with HSV-1. (A) miR-23a expression was determined by quantitative real-time PCR at indicated time. Fold-increase is shown compared with HeLa cells at each time. (B) Time course of IRF1 expression in HeLa cells infected with HSV-1. Fold-increase is shown compared with HeLa cells at each time.

The initial functional result was confirmed that miR-23a facilitated HSV-1 replication. A detailed time-course experiment further showed that miR-23a was not steadily increased or decreased in HSV-1-infected HeLa cells, reaching its peak expression as late as 18 h post-infection (Fig. 5A). This suggests that miR-23a induction could be the result of viral gene expression rather than viral binding. A similar time-course experiment showed that IRF1 was up-regulated within 1 h after exposure of HeLa cells to HSV-1, reaching its maximum expression at 4 h post-infection (Fig. 5B).

### IRF-1 inhibits virus replication partly through induction of RSAD2 expression

**Figure 6:**
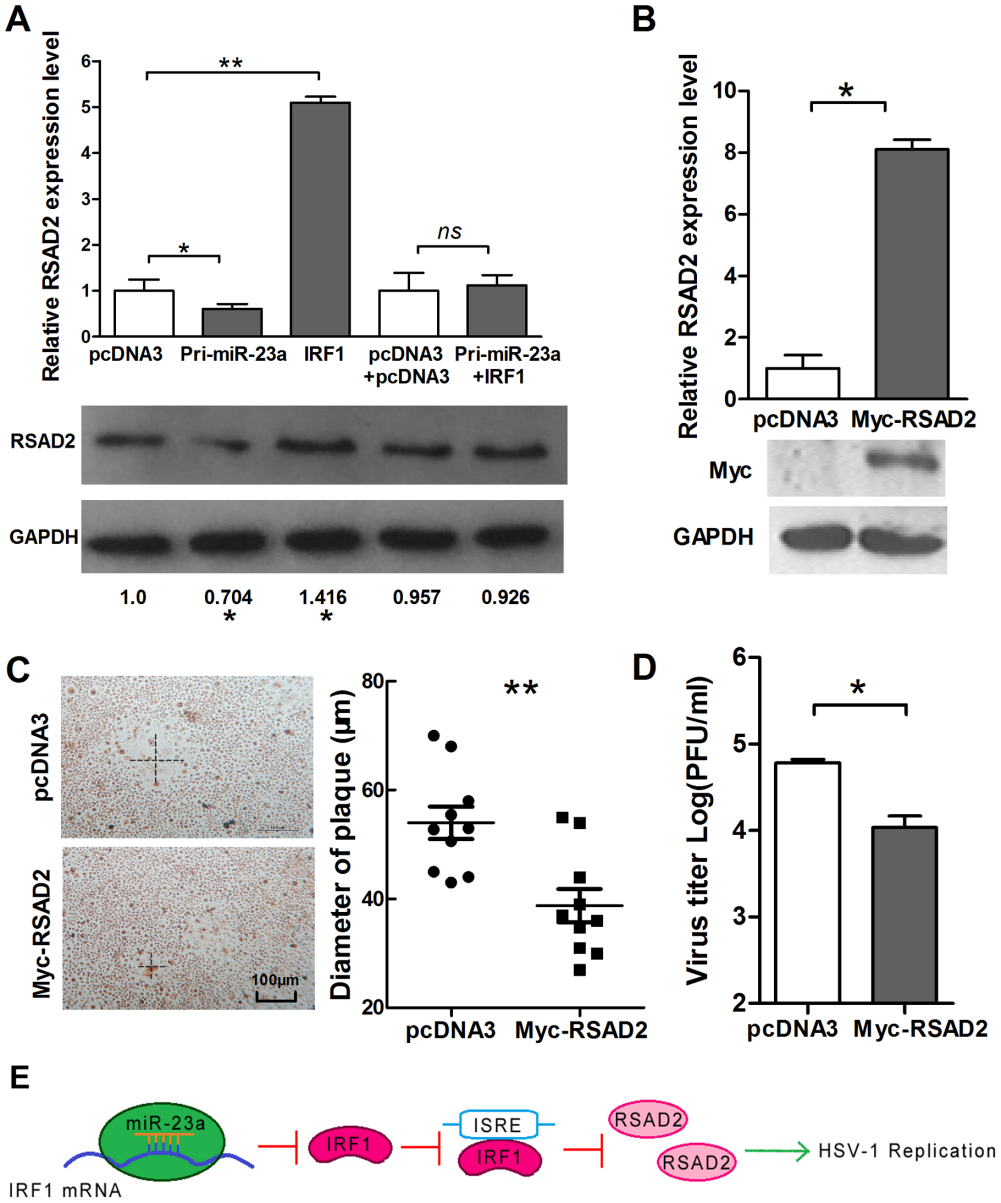
IRF1 suppresses the replication of HSV-1 partially by up-regulation of RSAD2. (A) HeLa cells were transfected with IRF1 and pcDNA3 or co-transfected with IRF1 and Pri-miR-23a and control vector, as indicated. Total RNA was extracted, and RSAD2 mRNA was quantified by quantitative real-time PCR. (B) HeLa cells were transfected with Myc-RSAD2. At 48-h post-transfection, quantitative real-time PCR was used to detect the level of RSAD2 mRNA, and at 72 h post-transfection, a Western blot was used to detect the expression level of RSAD2. (C) HeLa cells were transfected with Myc-RSAD2 or pcDNA3. Cells were infected with HSV-1 at 0.01 PFU/cell and stained with neutral red at 36 h post-infection. The mean radius of the cytopathic area was measured. The scale bar represents 100 µm. (D) HeLa cells were transfected with Myc-RSAD2 or pcDNA3. Viral yields were determined by standard plaque assays at 48 h post-infection with HSV-1. (E) Model of miR-23a regulation in HSV-1 replication. Increased levels of miR-23a in HeLa cells led to decrease levels of IRF1 mRNA and RSAD2 mRNA, with a consequent increase in HSV-1 replication. All data represent the mean value ± SD of at least three independent experiments. *: p<0.05; **: p<0.01; *ns*: No significant differences by Student's t test.

In a recent study, IRF1 suppressed VSV replication through radical S-adenosyl methionine domain containing 2 (RSAD2) induction, leading to the expression of viperin protein, which is involved in innate immune responses [35]. To determine whether IRF-1 suppresses HSV-1 replication via a similar pathway, we first determined RSAD2 mRNA levels in HeLa cells transiently transfected with IRF-1 expressing vector. Fig. 6A showed that IRF1 significantly enhanced RSAD2 expression at both mRNA and protein levels. In contrast, ectopic expression of miR-23a caused the amount of RSAD2 mRNA and protein to decrease by about 40% and 30%, respectively (Fig. 6A). Next, we first constructed a RSAD2 expression vector (Myc-RSAD2), and verified the efficiency of the vector by Western blot (Fig. 6B). Plaque-formation assay and viral-titer assay to further explore the role of RSAD2 in HSV-1 replication was positive. (Fig. 6C, D). Most likely, by targeting IRF1, miR-23a indirectly suppresses RSAD2 expression to facilitate HSV-1 replication (Fig. 6E).

## Discussion

Viruses often exploit cellular pathways to promote their life cycle. Because miRNAs are efficient regulators of gene expression that are both small and non-antigenic, they seem to be ideal tools to favor virus replication. Two classical examples of cellular miRNAs are the liver-specific miR-122 and miR-132 [36], [37]. Here, we examined the role of a host-encoded miR-23a in the promotion of viral replication.

Some studies suggest that miR-23a acts as an oncogene by regulating cell growth and apoptosis [15], [16], but few studies have examined its role in viral diseases. In our study, the neutral-red staining and standard plaque assay indicate strongly that miR-23a is involved in HSV-1 replication and mediates the promotion of viral replication (Fig. 1C, 1D). And the viral titer of supernatant further confirms a role for miR-23a in promoting HSV-1 replication (Fig. 1E).

IRF1 is a transcription activator with an important role in host–virus interaction [38]. Based on miR-23a served pro-virus function, IRF1 is supported it is to be a candidate target of miR-23a. Fluorescent-report assay indeed revealed it to be a target gene of miR-23a in HeLa cells. Initially, some studies showed that IRF-1 enables the activation of IFN-β transcription in cell culture [39], [40], but other experiments suggested that activation of IRF-1 also regulates genes that directly limit the replication of several viruses independent of IFN production [41]–[43]. Here, we demonstrated that the protection of host cells from HSV-1 infections by IRF-1 may partially depends on the enhancement of RASD2 expression, which is required for the innate immune response [44]. Although the miR-23a targets predicted by Targetsscan 6.2 suggest that miR-23a cannot directly target the RSAD2 UTR, we need to go further confirmed.

Time course of endogenous miR-23a and IRF1 expression are affected by HSV-1 infection (Fig. 5). However, the mechanism of miR-23a and IRF1 induction during HSV-1 infection remains largely unknown. During early HSV infection, the down-regulated miR-23a may be due to the host stress response which will initiate the antiviral system or suppress the virus-promoting system to prevent the virus infection. But during late infection, the virus antagonizing the host's defense, and the virus antigen expression and replication may both induce miR-23a expression and other virus-promoting system to benefit its own infection. The specific mechanism is under investigation. And it is unclear whether IRF1 as a transcription factor would regulates miR-23a level.

Furthermore, recent studies have shown that IRF1 is involved in regulation of apoptosis. For example, IRF1-dependent transcriptional activation of caspase 8 regulates the apoptotic pathway [45], and up-regulation of miR-23a permits anti-caspase-dependent apoptosis in several types of human cells [46], [47]. Apoptosis, or programmed cell death, occurs in response to various stimuli, including virus infection. Viruses can modulate apoptotic pathways to enhance survival of the infected cell. For HSV-1, apoptosis is triggered by the transcription of immediate-early genes, such as ICP0 during infection [48]. And miRNA-dependent regulation generally involves a complex network. These suggest that miR-23a facilitates virus replication by down-regulating IRF1 mRNA to suppress RSAD2 expression and apoptosis.

But the mechanism responsible for the IRF1 suppressing HSV-1 replication is unclear. It is well known that IRF1 can stimulate both IFN I and IFN III system [49], [50]. Moreover, IRFI can activate many ISGs in an IFN-independent manner [51]. So both IFN system and IFN-independent pathway may be involved in anti-viral effect of IRF1 against HSV-1. Next, we choose RSAD2 or viperin for its recently reported effect on VSV. And as one of ISGs, it can be induced by both IFN-dependent pathway and directly by IRF1 [35]. Compared the result of fig. 3E and Fig. 6D, we can see that RSAD2 may partially account for the suppressing effect on HSV-1 by IRF1, although the anti-viral role of RSAD2 in IRF1 suppressing HSV-1 needs further investigation. Surprisingly, our finding was inconsistent with a recent study which showed that ectopically expressed RSAD2 could not inhibit the replication of wild type HSV-1 in HEK293T cells [52]. This may be due to different MOI used and different detection time, and more importantly, the replication cycle of HSV-1 in HeLa cells may be not the same as in HEK293T cells. The specific reason was under investigation. For the regulation of RSAD2 expression by IRF1, both IFN system and IFN-independent pathway may be involved, which needs further validation. Thus, IRF1 may suppress HSV replication partially by up-regulation of RSAD2 in both IFN-dependent and IFN-independent manner. We will explore the specific mechanism in the future.

In conclusion, we found that the influence of miR-23a on virus replication is mediated by IRF-1 and proposed the model depicted in Fig. 6E. This model shows the probable pathways by which miR-23a can promote viral replication, which is involved in the down-regulation of RSAD2, an anti-viral gene. However, whether HSV-1 infection could induce miR-23a expression and miR-23a has a similar function during infection with other viruses remain a subject for future study.
