# Expression of Concern: MiR-23a Facilitates the Replication of HSV-1 through the Suppression of Interferon Regulatory Factor 1

**DOI:** 10.1371/journal.pone.0234092

**Published:** 2020-05-29

**Authors:** The PLOS ONE Editors

Following publication of this article [1], concerns were raised that the following panels appear to show overlapping data:

- Fig 1F, HSV-1 glycoprotein and DAPI panels for pcDNA3 and pRNAT-U6.2
- Fig 1F, HSV-1 glycoprotein and DAPI panels for Anti-miR-23a (left portion) in Fig 1F and pSilencer (right portion) in Fig 3F

**Fig 1:**
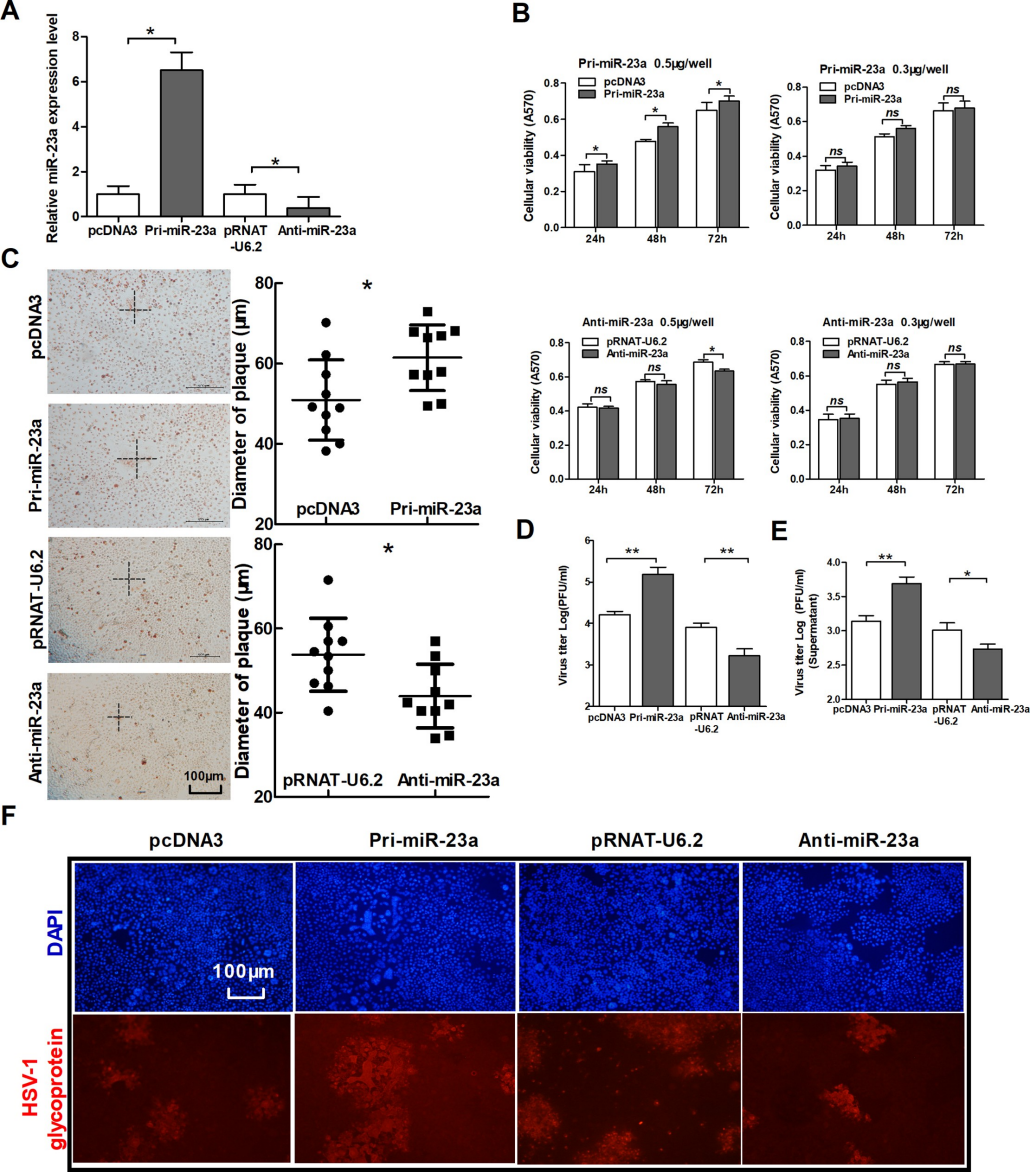
MiR-23a promotes the replication of HSV-1. (A) HeLa cells were transfected with Pri-miR-23a, pcDNA3, Anti-miR-23a and pRNAT-U6.2, respectively. At 24 h post-transfection, total RNA was extracted and analyzed for miR-23a expression by quantitative real-time PCR. (B) HeLa cells were transfected as indicated in (A). Cell viability was measured by MTT assay at 24 h, 48 h and 72 h post-transfection. To up-regulate miR-23a, two doses of vectors were used for transfection, 0.5 μg/well and 0.3 μg/well. Another group was transfected with Anti-miR-23a and its control vector in the same way. (C–F) HeLa cells were transfected as indicated in (A), 24 h post-transfection, cells were infected with HSV-1 at 0.01 PFU/cell. At 48 h post-infection, the radius of the cytopathic area was measured by neutral red staining. The scale bar represents 100 μm (C). Total viral yields (D) and yield of progeny virions from the culture supernatant (E) were determined by standard plaque assays. Level of glycoprotein expression was determined by immunofluorescence assay (F). All data represent the mean value ± SD of at least three independent experiments. *: p<0.05; **: p<0.01; ***: p<0.001; ns: No significant differences by Student's t test.

The authors noted that incorrect images are reported in Fig 1F for pRNAT-U6.2 and Anti-miR-23a, and that the correct image data are reported in Fig 3F. They provided an updated Fig 1 using the correct images from the original experiment, along with supporting image files for Figs 1F and 3F in S1 and S2 Files.

In addition, the Data Availability statement for this article reads “All relevant data are within the paper.” However, the primary data are not included with the article and the authors noted that except for the data in S1 and S2 Files the original data for this study are no longer available. As such, the article does not comply with *PLOS ONE*’s Data Availability policy that was in place at the time of the article’s submission.

In light of the above concerns, the *PLOS ONE* Editors issue this Expression of Concern.

The Data Availability statement is updated to: Image data for Figs 1F and 3F are in the Supporting Information files of this notice [1]. The original data underlying other results reported in the article are no longer available.

## Supporting information

S1 File Original image files supporting Fig 1F. Level of glycoprotein expression was determined by immunofluorescence assay for the group of Pri-miR-23a and its control (pcDNA3), and the group of Anti-miR-23a and its control (pRNAT-U6.2). (ZIP)

S2 File Original image files supporting Fig 3F. Level of glycoprotein expression was determined by immunofluorescence assay for the group of IRF1 and its control (pcDNA3), and the group of sh-IRF1 and its control (pSilencer). (ZIP)
