# Correction: Expression of Concern: MiR-23a Facilitates the Replication of HSV-1 through the Suppression of Interferon Regulatory Factor 1

**DOI:** 10.1371/journal.pone.0265925

**Published:** 2022-03-17

**Authors:** The PLOS ONE Editors

After publication of this article [1] and Expression of Concern [2], concerns were raised that additional panels appear to show overlapping data that were not previously identified. Specifically, in Fig 3F the DAPI and HSV-1 glycoprotein panels for IRF1 appear to partially overlap with the DAPI and HSV-1 glycoprotein panels for sh-IRF1.

**Fig 3:**
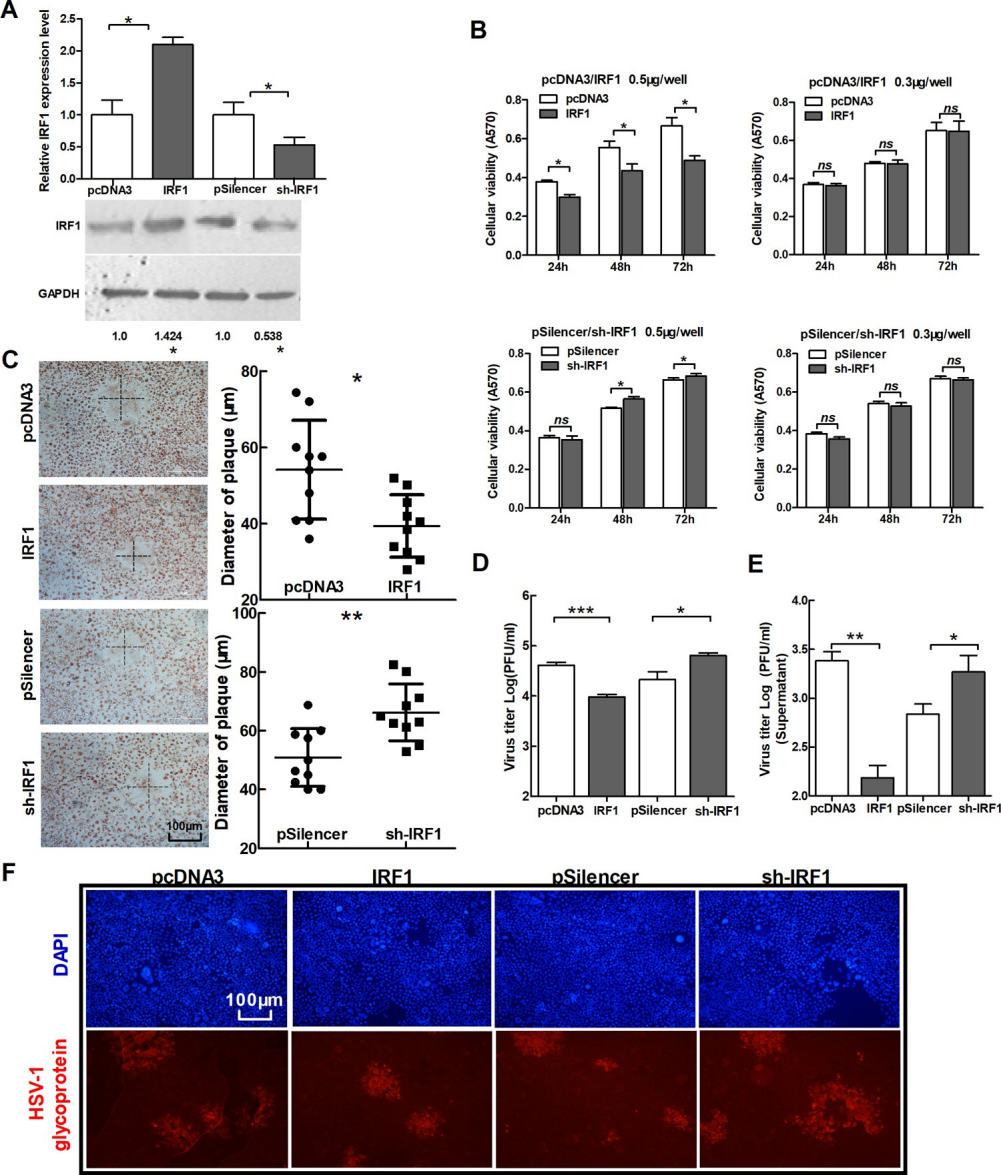
IRF1 suppresses the replication of HSV-1. (A) HeLa cells were transfected with IRF1, sh-IRF1 and control vectors, respectively. Total RNA was extracted and analyzed for IRF1 mRNA by quantitative real-time PCR. The cell lysate was extracted and analyzed for IRF1 expression by Western blot. (B) HeLa cells were transfected as indicated in (A), MTT assay of cell viability was conducted at 24 h, 48 h and 72 h post-transfection. To up-regulate IRF1, two doses of vectors were used for transfection, 0.5 μg/well and 0.3 μg/well. Another group was transfected with sh-IRF1 and its control vector in the same way. (C–F) HeLa cells were transfected as indicated in (A), 24 h post-transfection, cells were infected with HSV-1 at 0.01 PFU/cell. At 48 h post-infection, cells were stained with neutral red. The mean radius of the cytopathic area was measured. The scale bar represents 100 μm (C). Total viral yields (D) and Yield of progeny virions from the culture supernatant (E) were determined by standard plaque assays. Level of glycoprotein expression was determined by immunofluorescence assay (F). All data represent the mean value ± SD of at least three independent experiments. *: p<0.05; **: p<0.01; ***: p<0.001; ns: No significant differences by Student’s t test.

The authors stated that this overlap was due to an error in figure preparation, and that the IRF1 panels are incorrect. They provide here an updated version of Fig 3F in which the DAPI and HSV-1 glycoprotein panels for IRF1 are replaced. They also provide an updated version of S2 File from [2] which they say contains the correct primary data supporting Fig 3F and replicate samples from the same experiment.

The *PLOS ONE* Editors apologize that this issue was not identified prior to publication of the Expression of Concern [2].

## Supporting information

S2 File Original image files supporting Fig 3F. Level of glycoprotein expression was determined by immunofluorescence assay for the group of IRF1 and its control (pcDNA3), and the group of sh-IRF1 and its control (pSilencer).
(ZIP)
