# Supplementary figures and images for: Expression of Concern: MiR-23a Facilitates the Replication of HSV-1 through the Suppression of Interferon Regulatory Factor 1
Source: PLoS One. 2020 May 29;15(5):e0234092. doi: 10.1371/journal.pone.0234092 (PMC7259501; doi:10.1371/journal.pone.0234092)

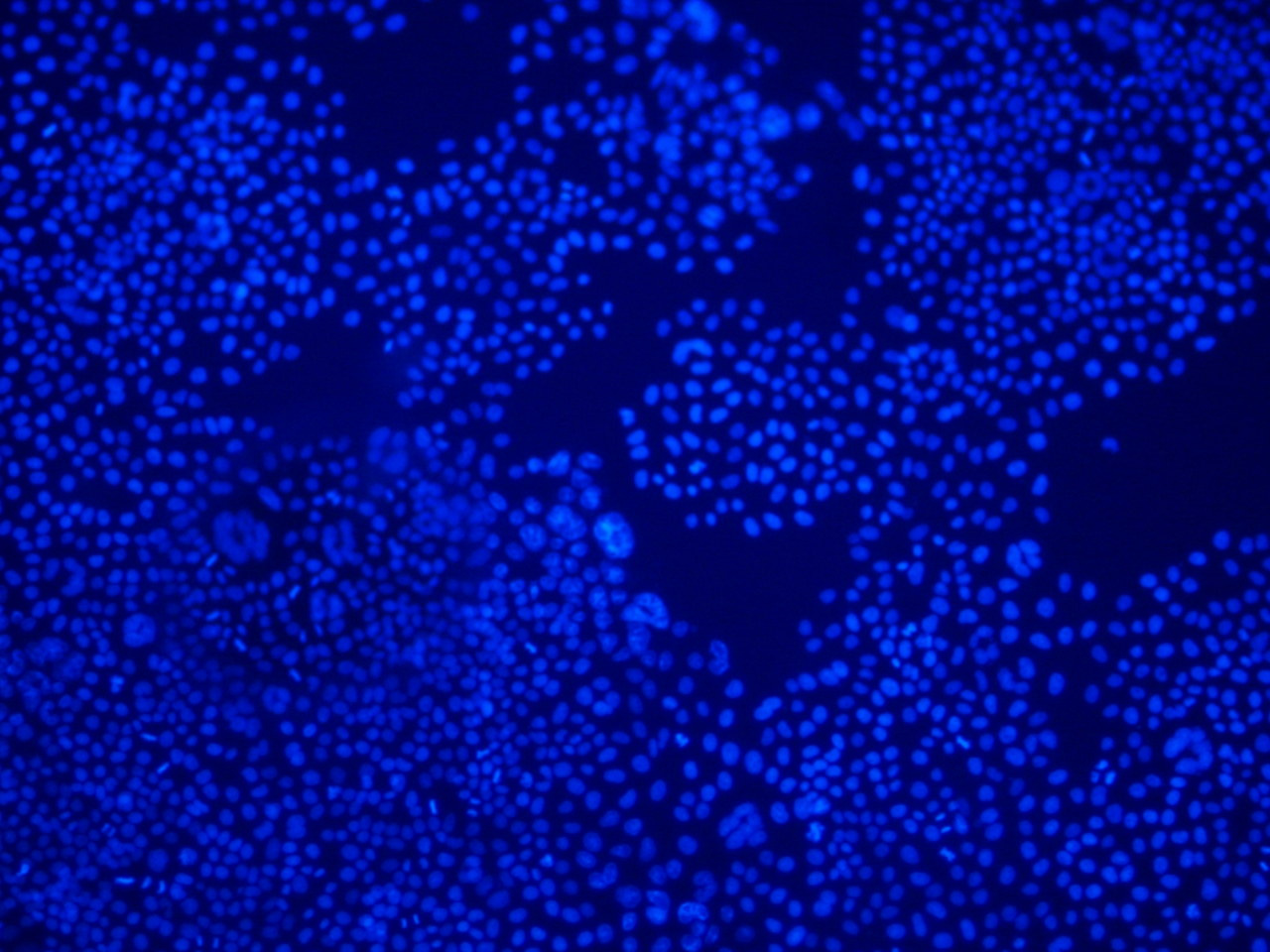

Supplement: S1 File — (ZIP) [file pone.0234092.s001.zip › S1 File/DAPI for Anti-miR-23a(updated figure).tif]

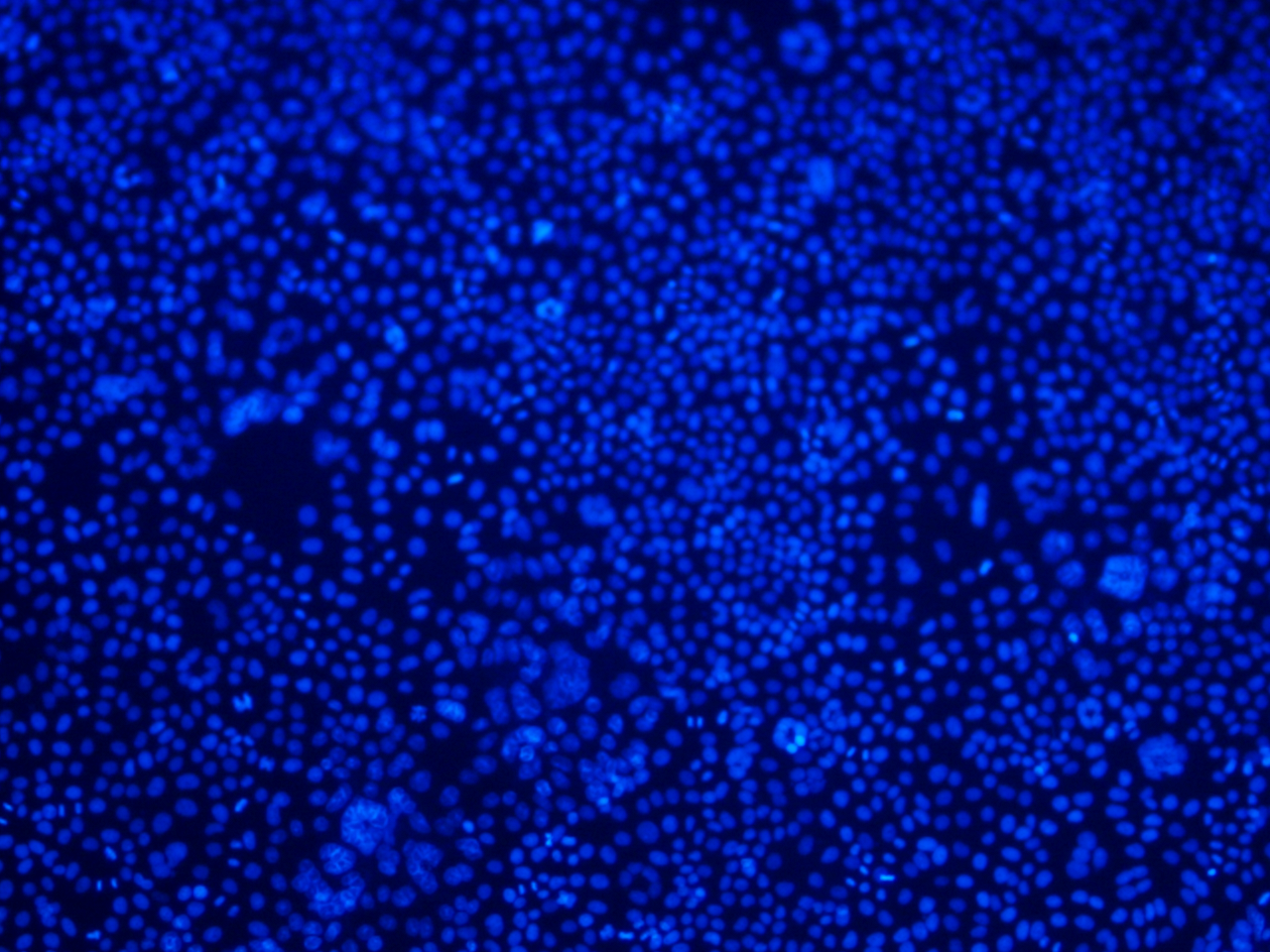

Supplement: S1 File — (ZIP) [file pone.0234092.s001.zip › S1 File/DAPI for pcDNA3(published figure).tif]

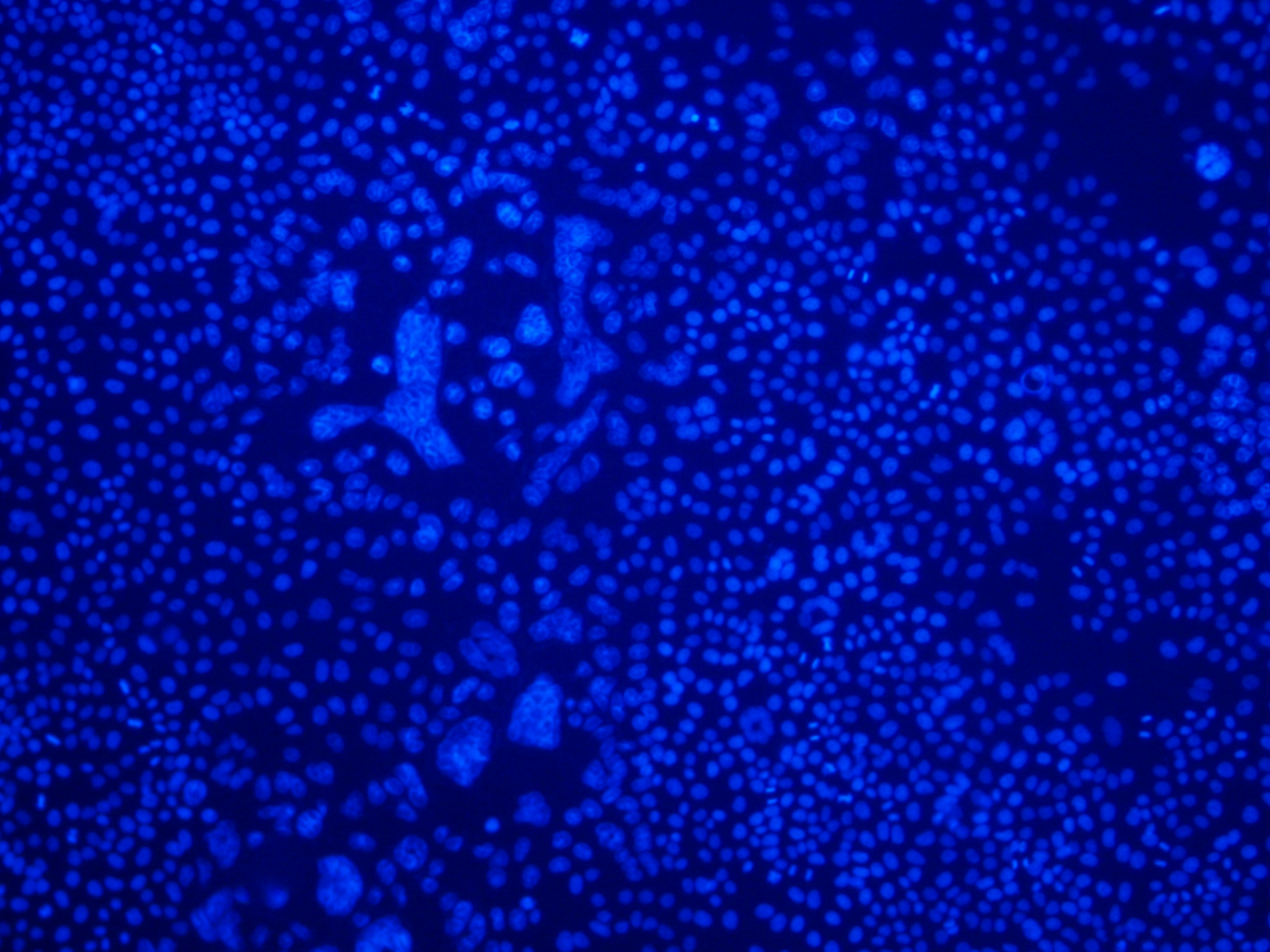

Supplement: S1 File — (ZIP) [file pone.0234092.s001.zip › S1 File/DAPI for Pri-miR-23a(published figure).tif]

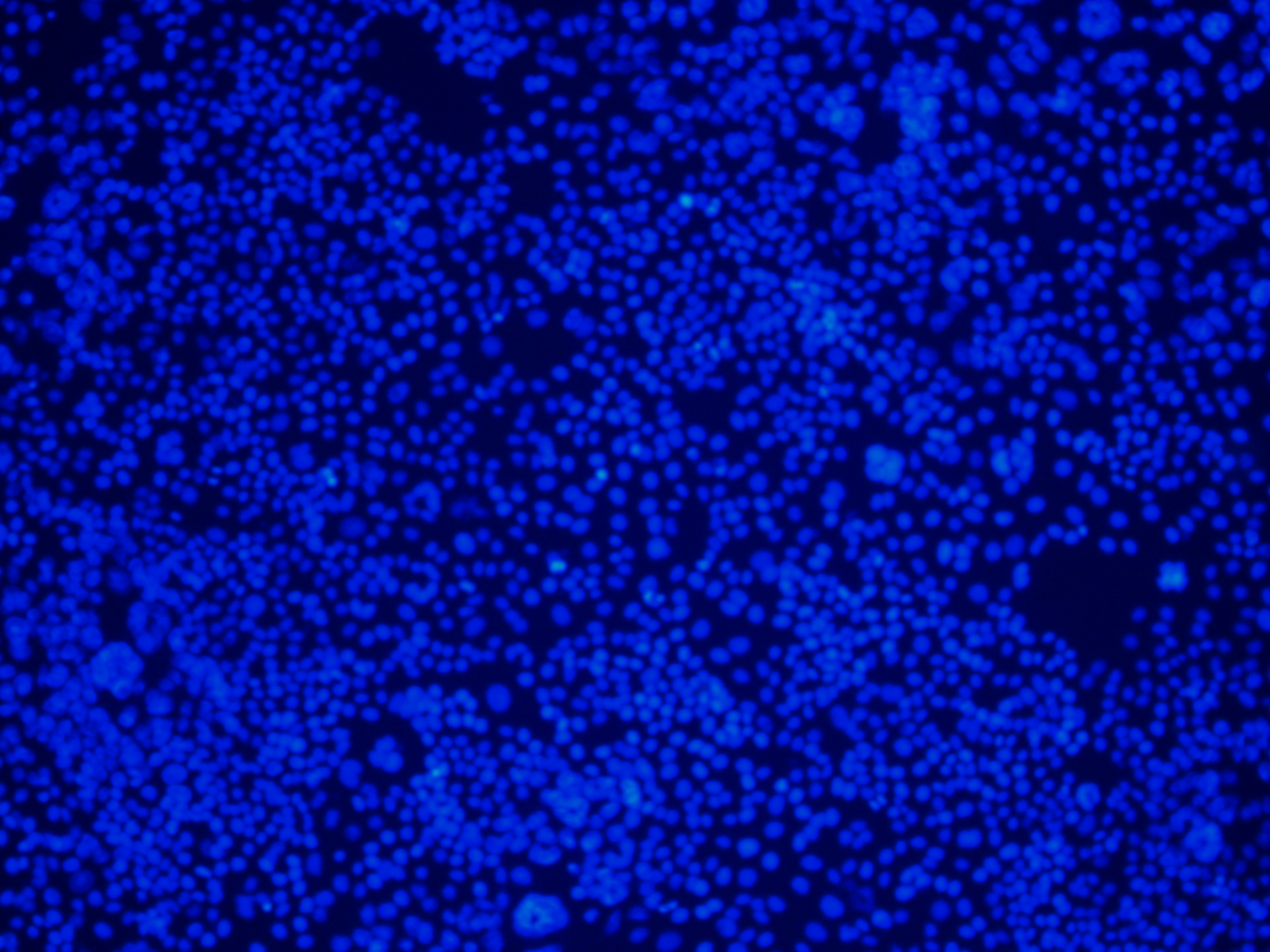

Supplement: S1 File — (ZIP) [file pone.0234092.s001.zip › S1 File/DAPI for pRNAT-U6.2(updated figure).tif]

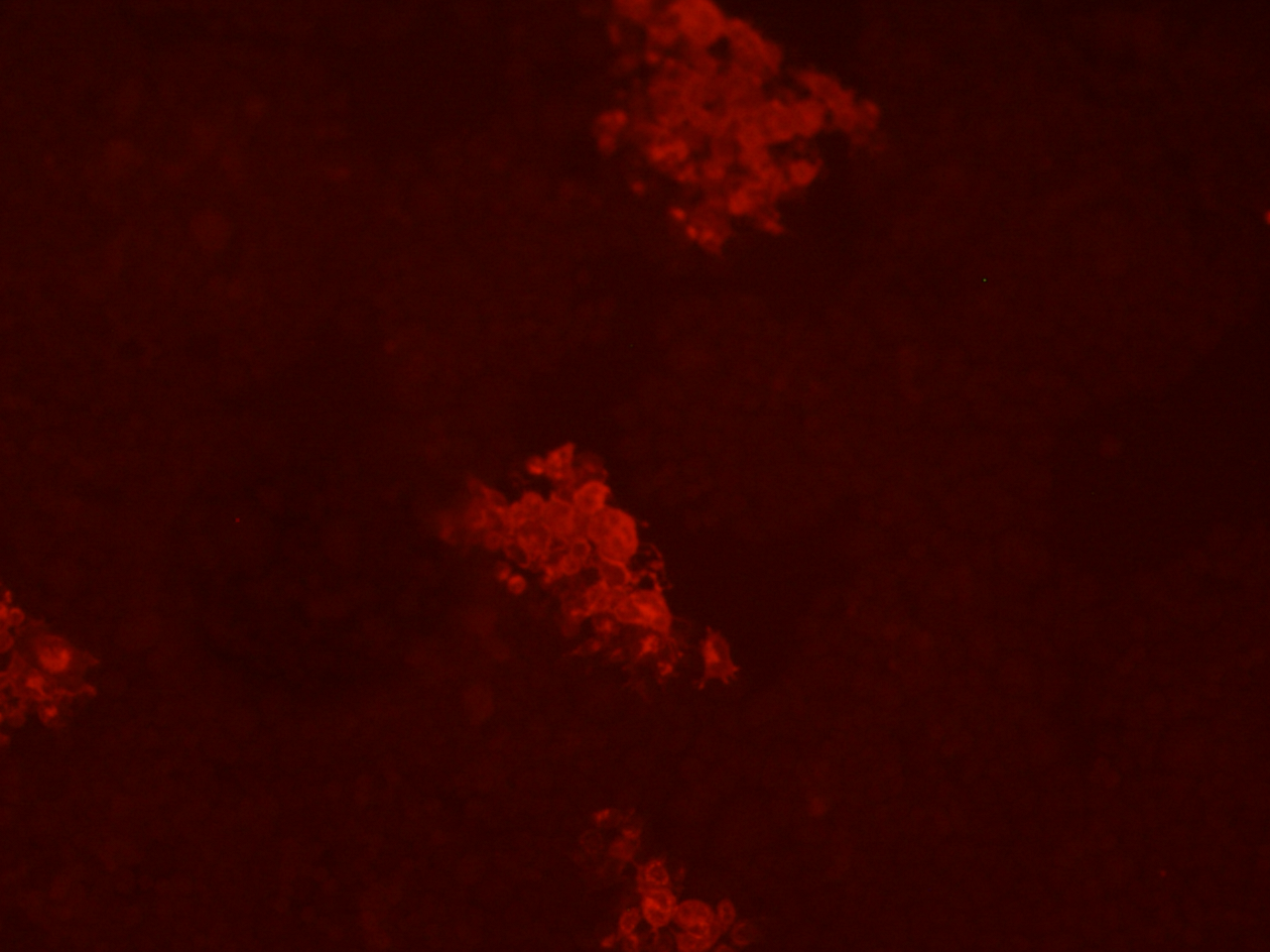

Supplement: S1 File — (ZIP) [file pone.0234092.s001.zip › S1 File/HSV-1 glycoprotein for Anti-miR-23a(updated figure).tif]

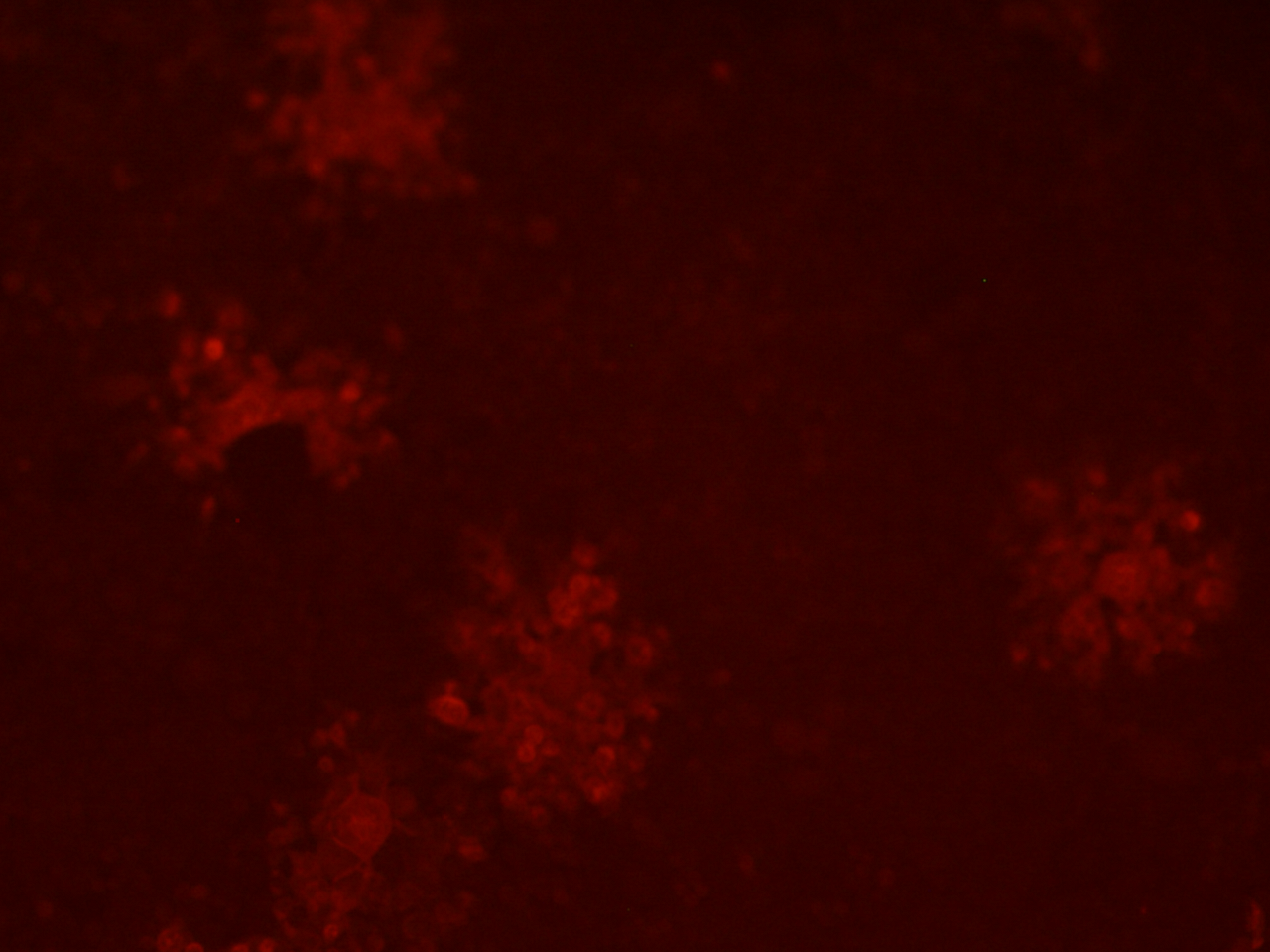

Supplement: S1 File — (ZIP) [file pone.0234092.s001.zip › S1 File/HSV-1 glycoprotein for pcDNA3( published figure).tif]

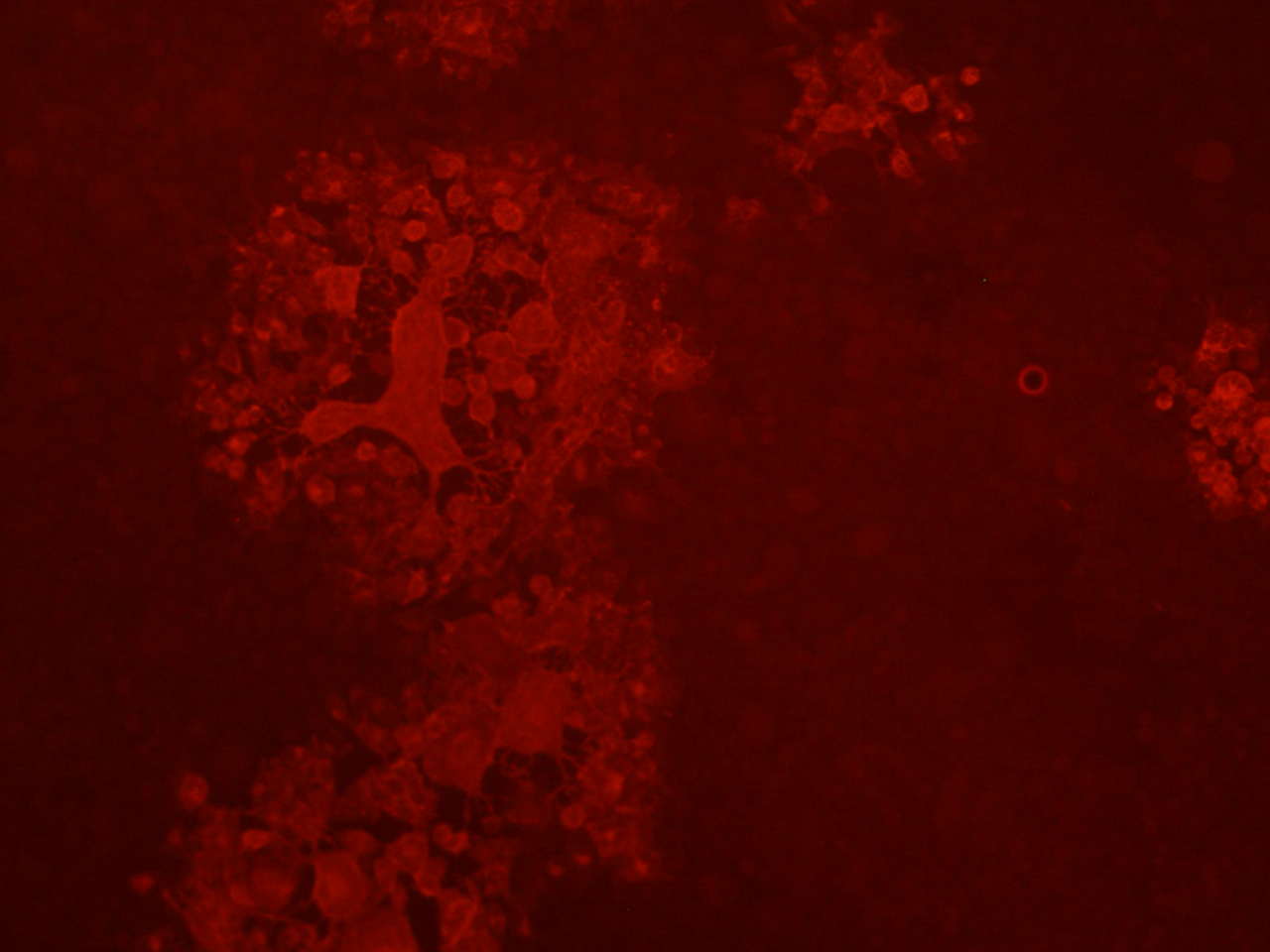

Supplement: S1 File — (ZIP) [file pone.0234092.s001.zip › S1 File/HSV-1 glycoprotein for Pri-miR-23a( published figure).tif]

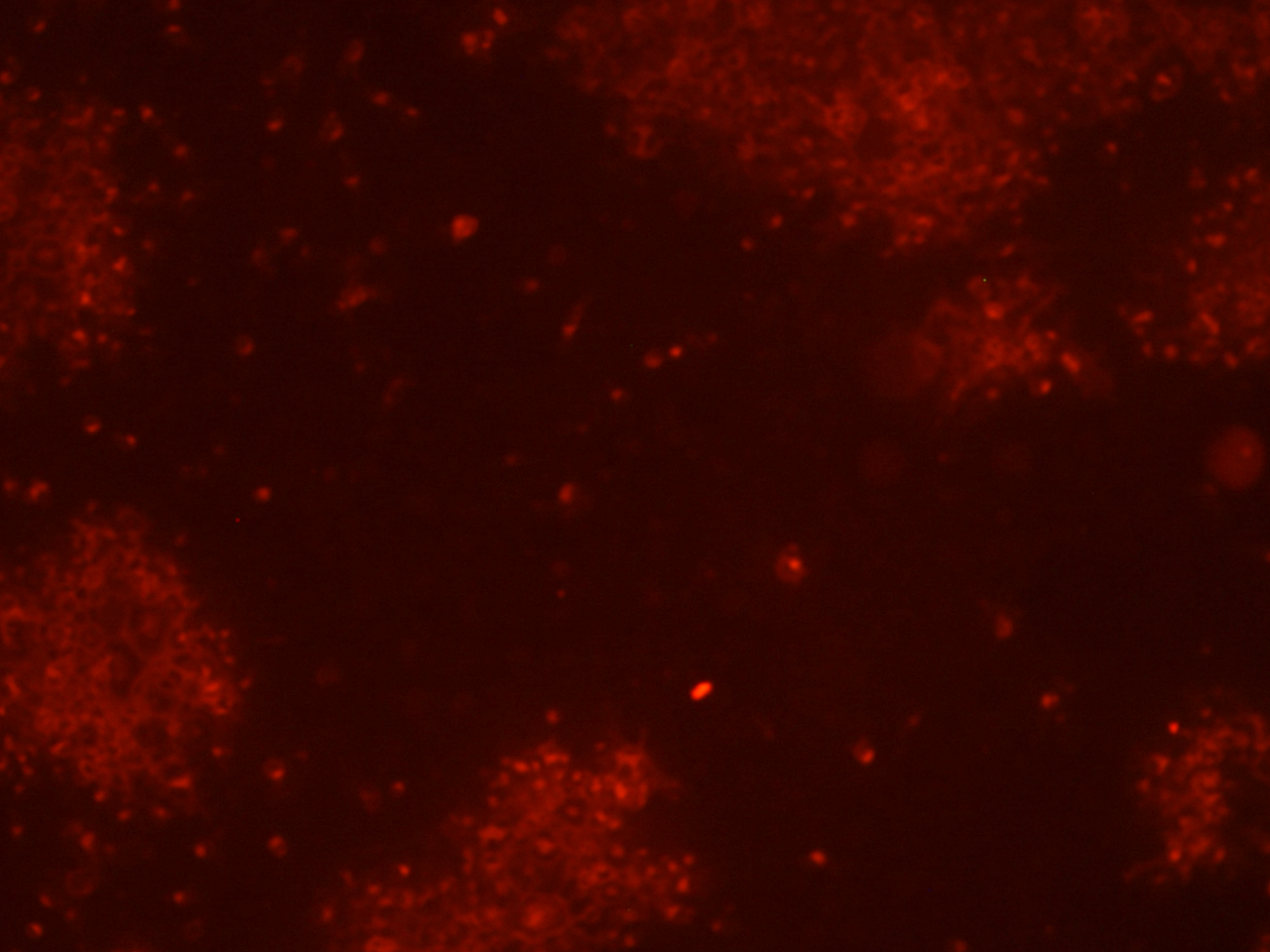

Supplement: S1 File — (ZIP) [file pone.0234092.s001.zip › S1 File/HSV-1 glycoprotein for pRNAT-U6-2(updated figure).tif]

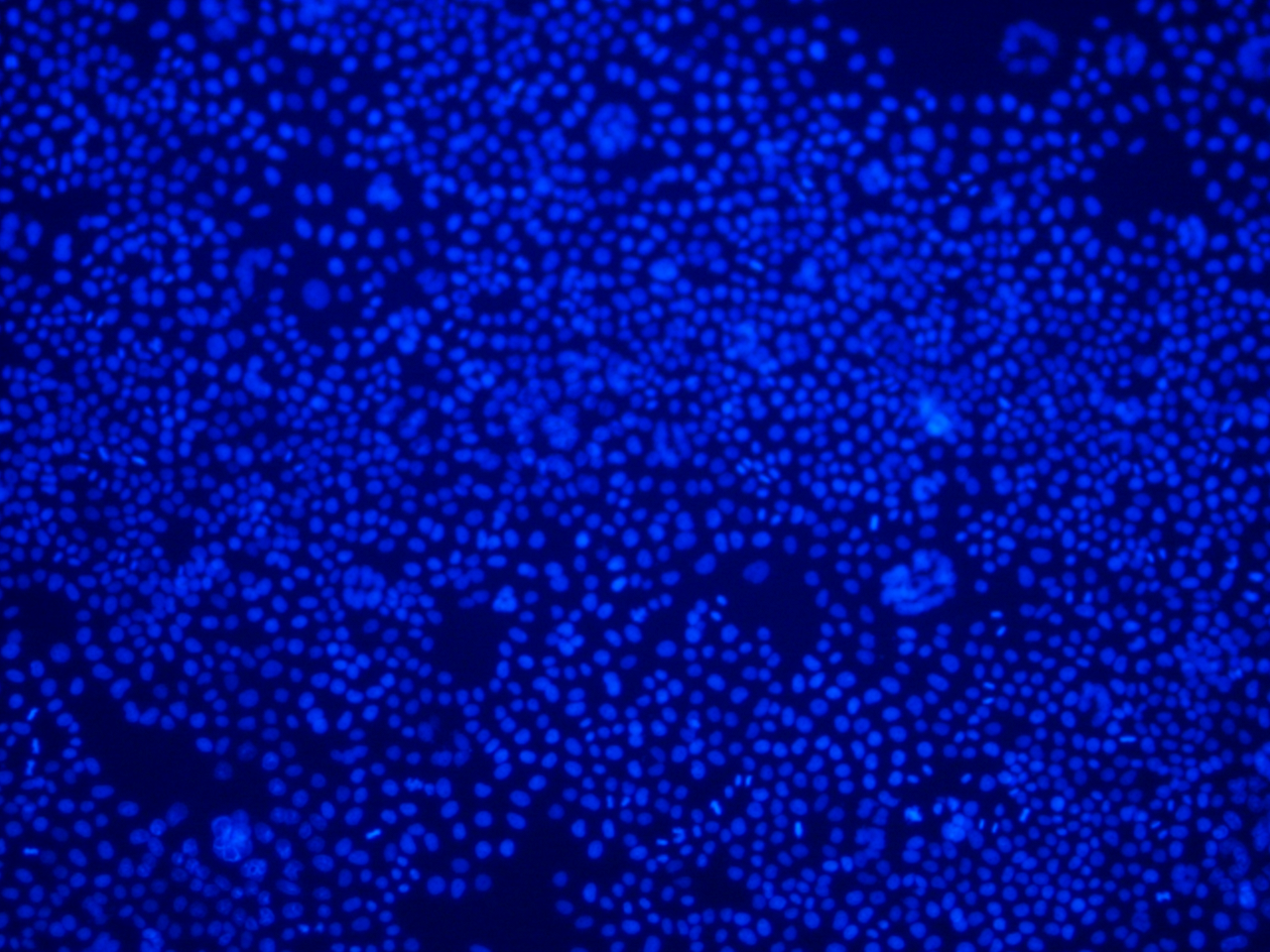

Supplement: S2 File — (ZIP) [file pone.0234092.s002.zip › S2 File/DAPI for IRF1(published figure).tif]

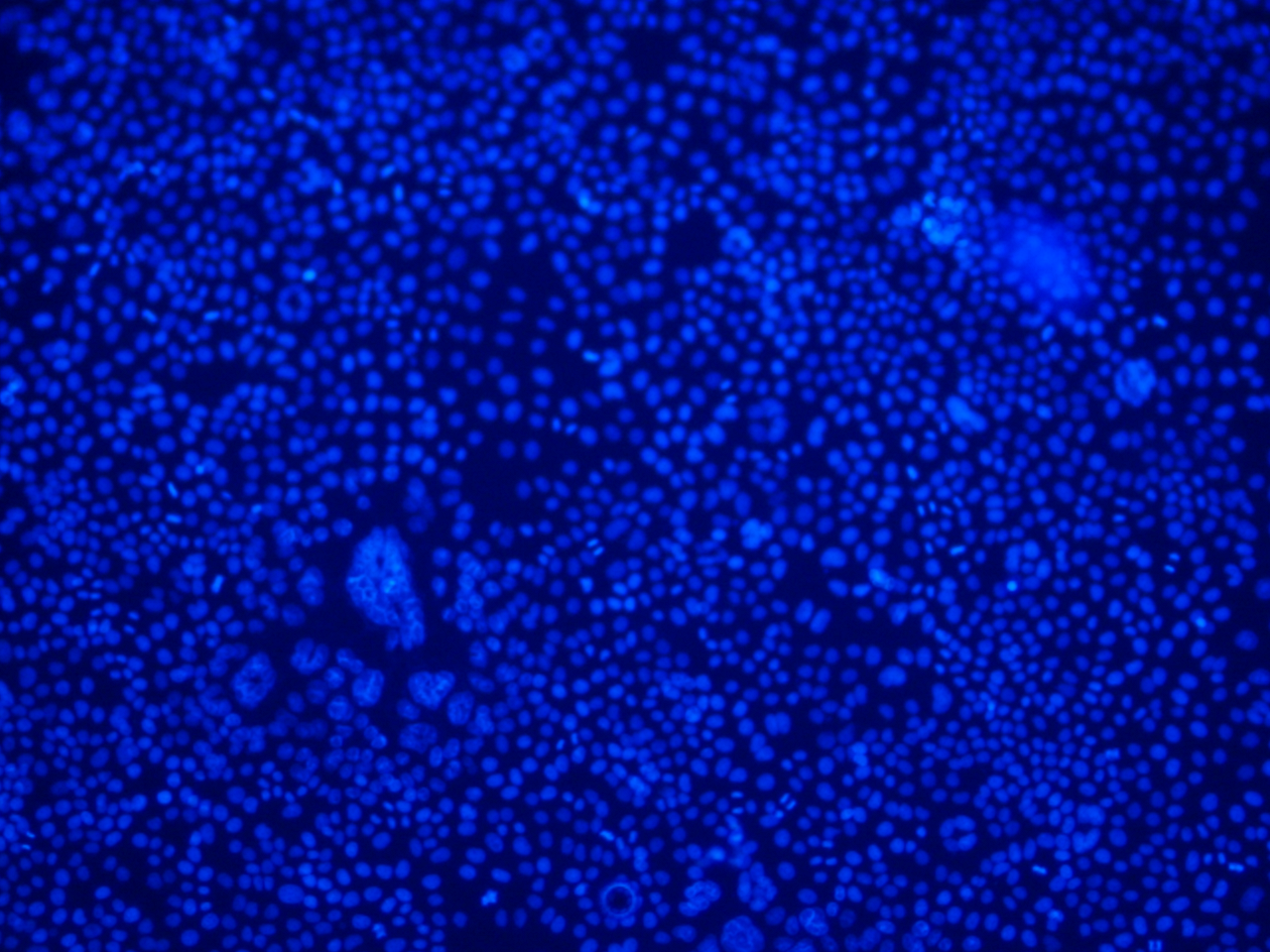

Supplement: S2 File — (ZIP) [file pone.0234092.s002.zip › S2 File/DAPI for pcDNA3(published figure).tif]

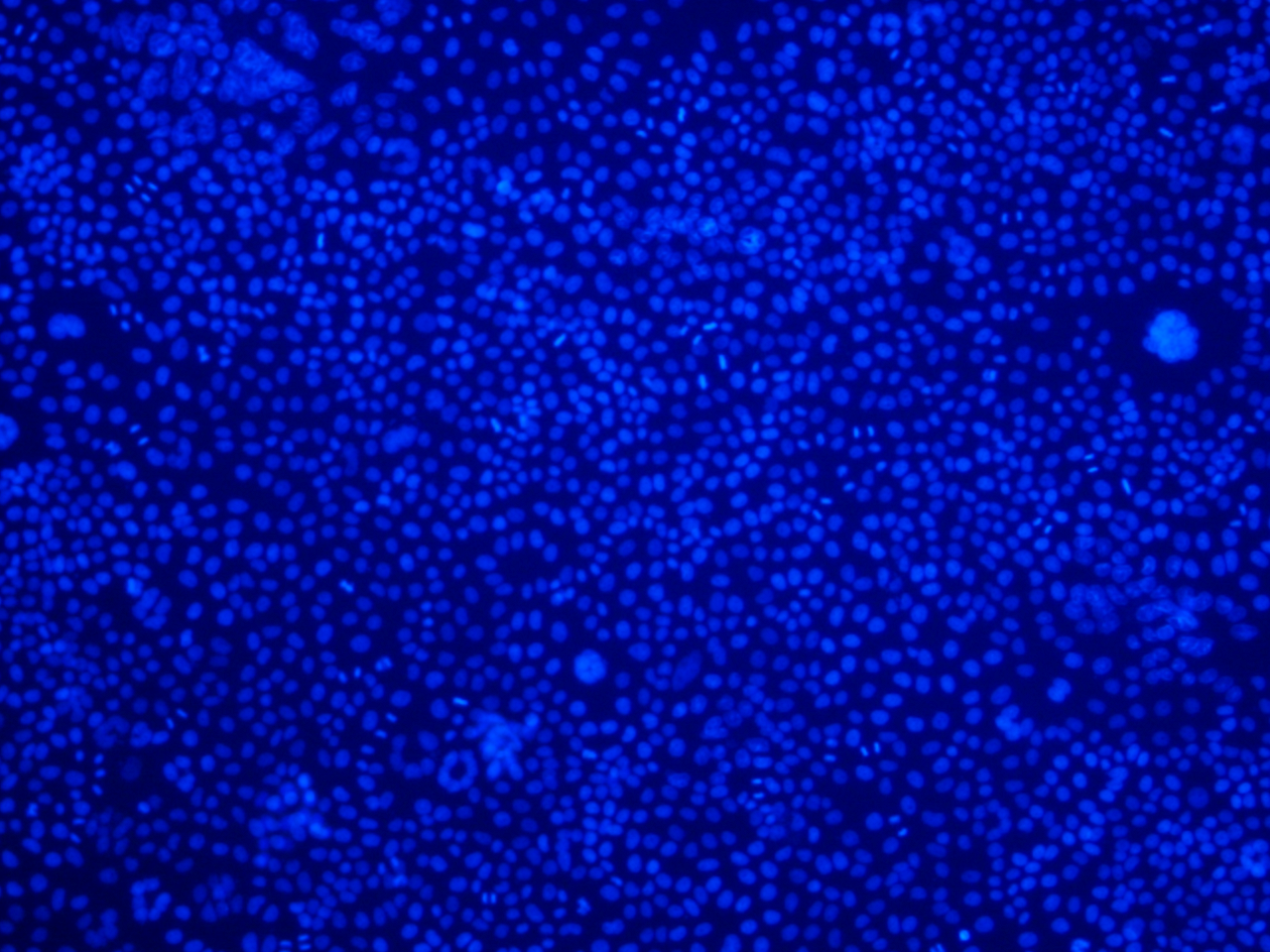

Supplement: S2 File — (ZIP) [file pone.0234092.s002.zip › S2 File/DAPI for pSilencer(published figure).tif]

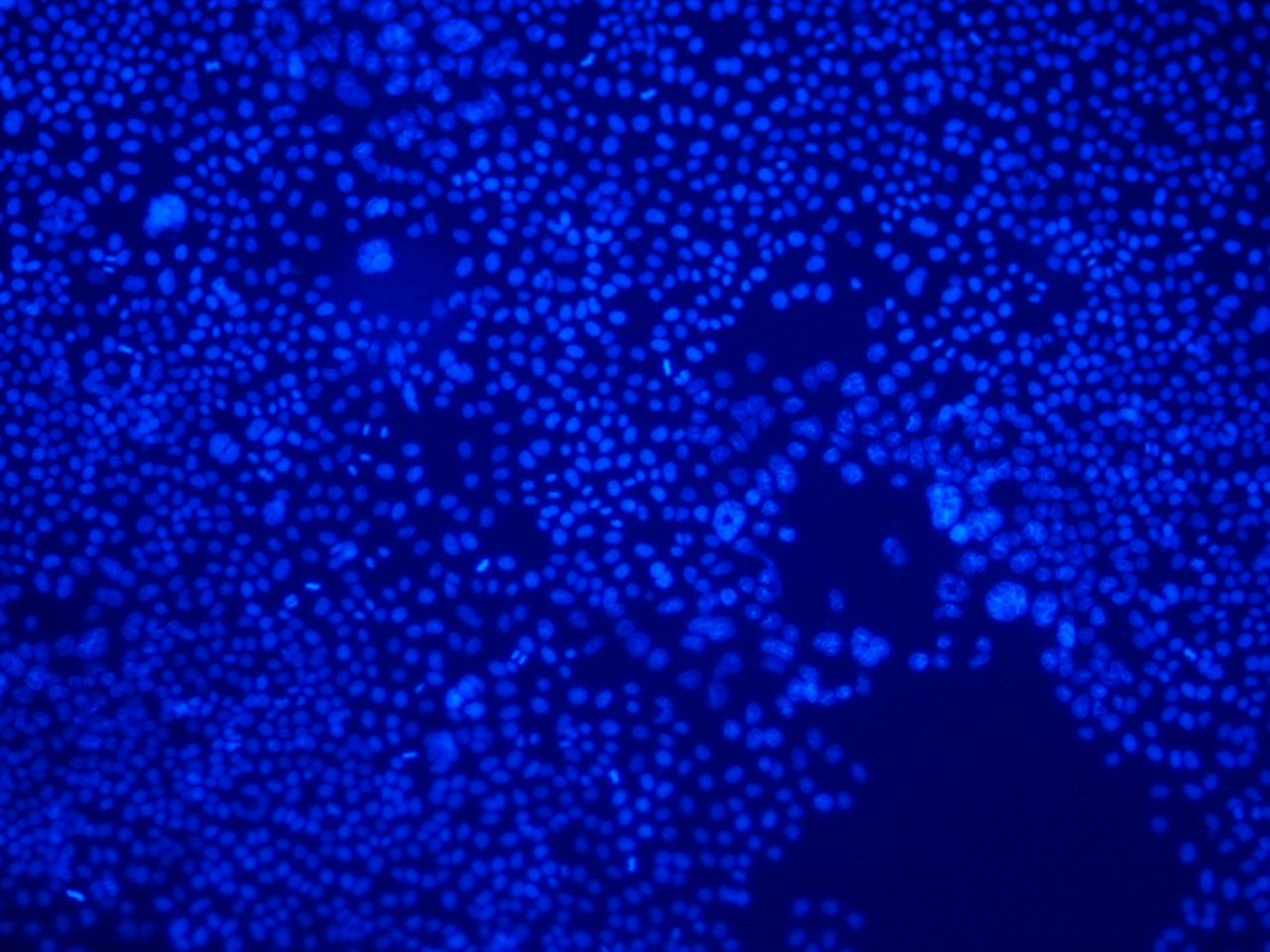

Supplement: S2 File — (ZIP) [file pone.0234092.s002.zip › S2 File/DAPI for sh-IRF1(published figure).tif]

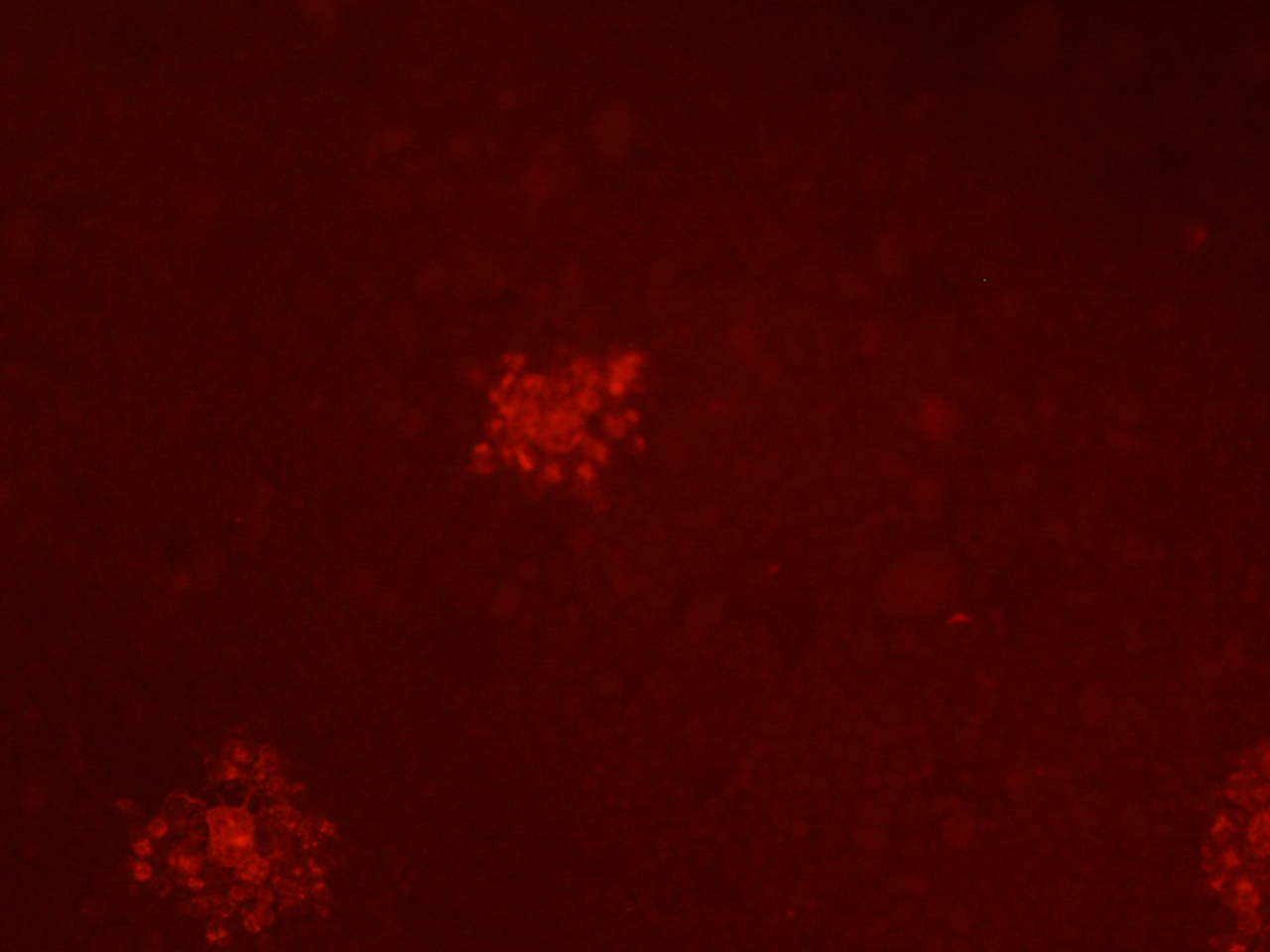

Supplement: S2 File — (ZIP) [file pone.0234092.s002.zip › S2 File/HSV-1 glycoprotein for IRF1(published figure).tif]

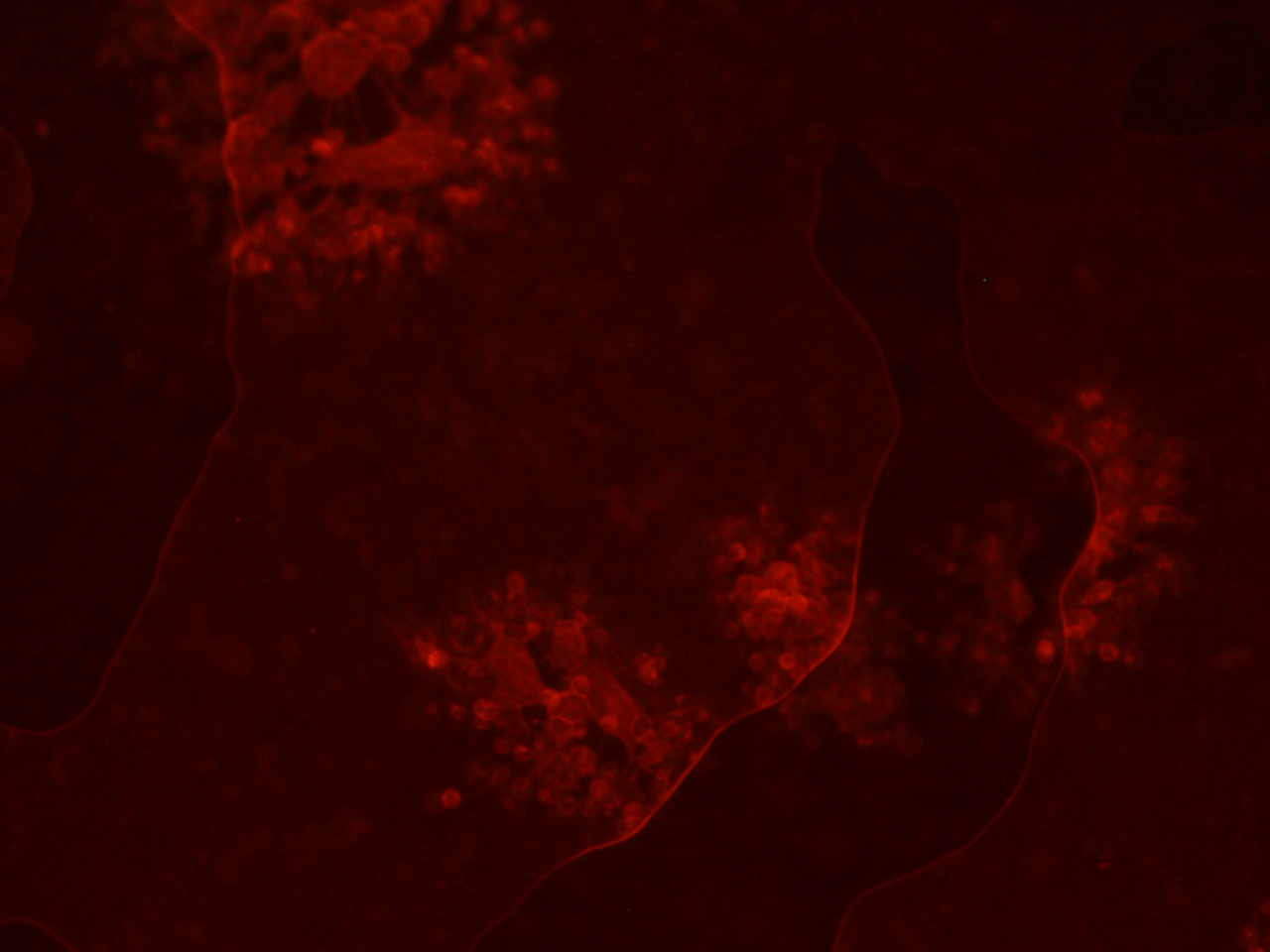

Supplement: S2 File — (ZIP) [file pone.0234092.s002.zip › S2 File/HSV-1 glycoprotein for pcDNA3(published figure).tif]

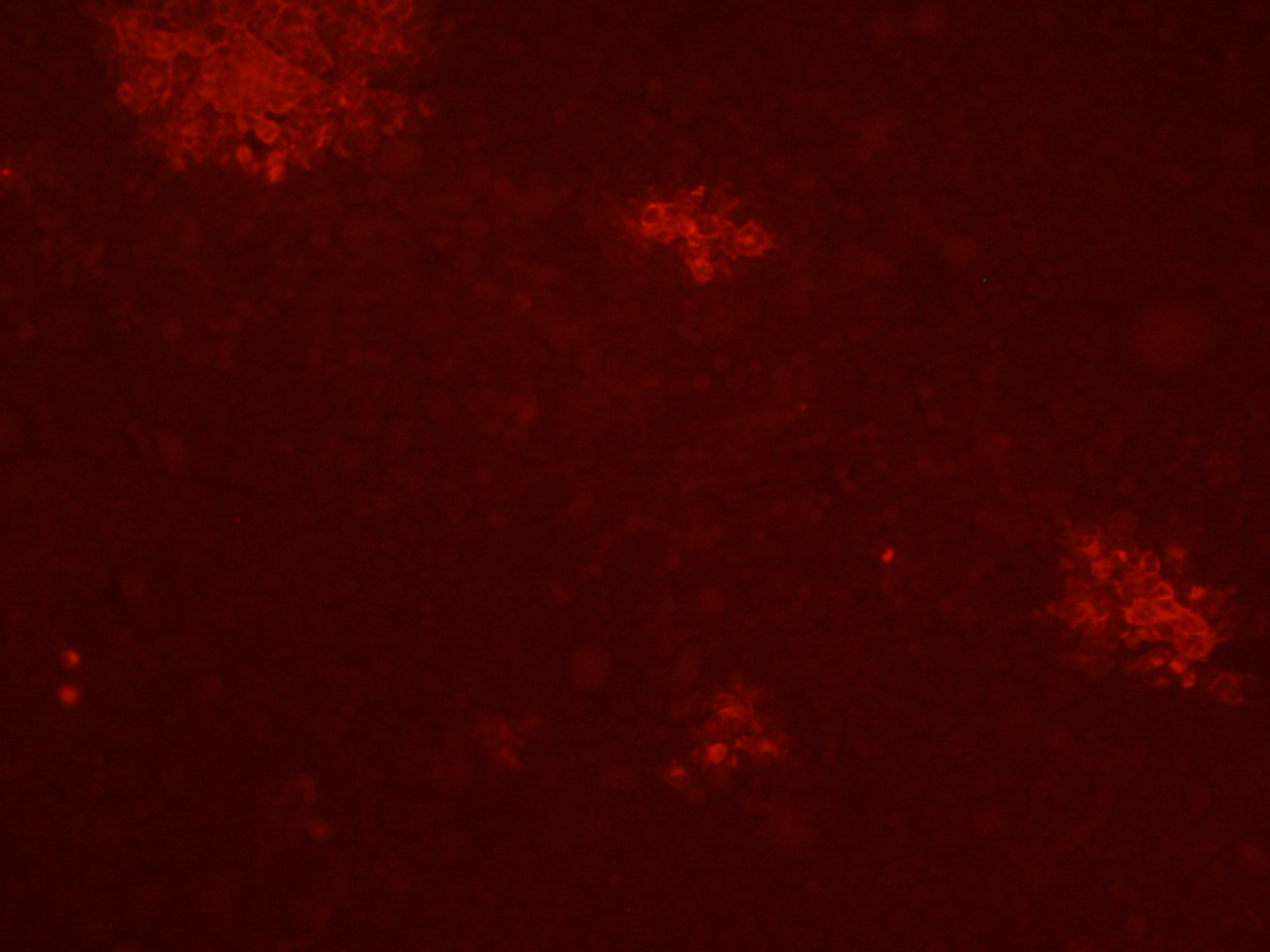

Supplement: S2 File — (ZIP) [file pone.0234092.s002.zip › S2 File/HSV-1 glycoprotein for pSilencer(published figure).tif]

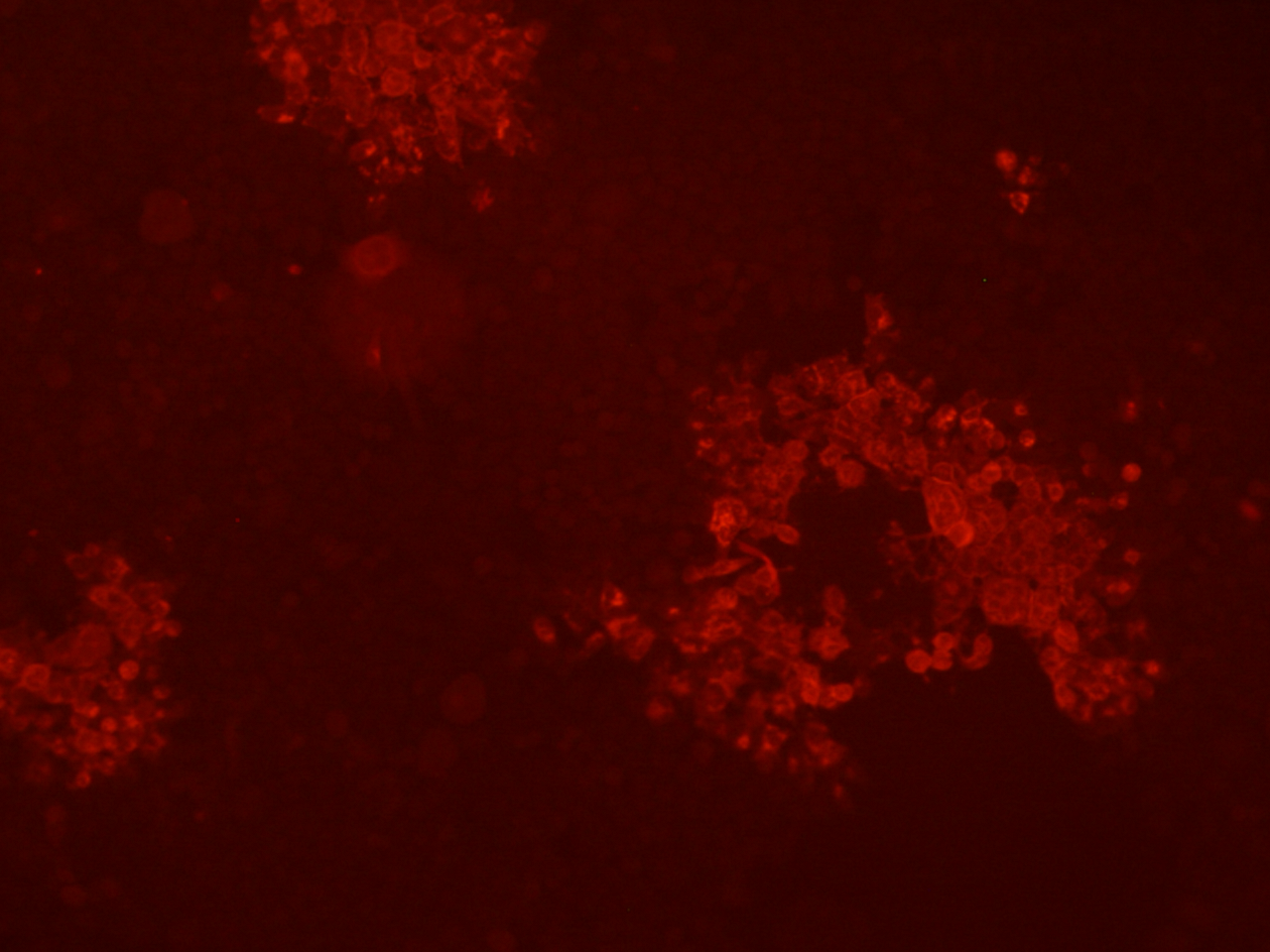

Supplement: S2 File — (ZIP) [file pone.0234092.s002.zip › S2 File/HSV-1 glycoprotein for sh-IRF1(published figure).tif]
