# Supplementary figures and images for: Correction: Expression of Concern: MiR-23a Facilitates the Replication of HSV-1 through the Suppression of Interferon Regulatory Factor 1
Source: PLoS One. 2022 Mar 17;17(3):e0265925. doi: 10.1371/journal.pone.0265925 (PMC8929627; doi:10.1371/journal.pone.0265925)

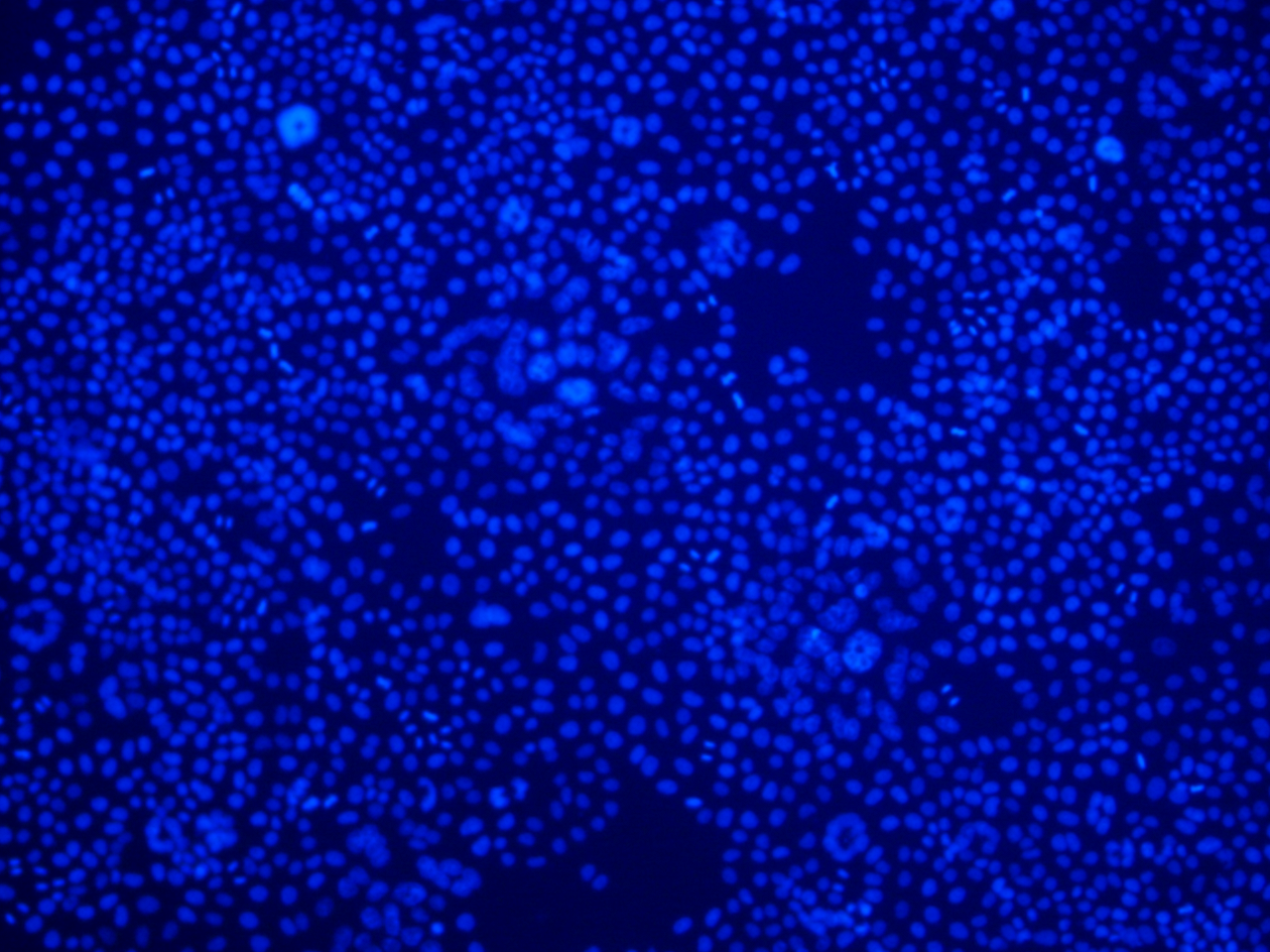

Supplement: S2 File — Level of glycoprotein expression was determined by immunofluorescence assay for the group of IRF1 and its control (pcDNA3), and the group of sh-IRF1 and its control (pSilencer). (ZIP) [file pone.0265925.s001.zip › Repeated experiment Fig 3F_pone.0114021/IRF1/IRF1-1-DAPI in Fig 3F.tif]

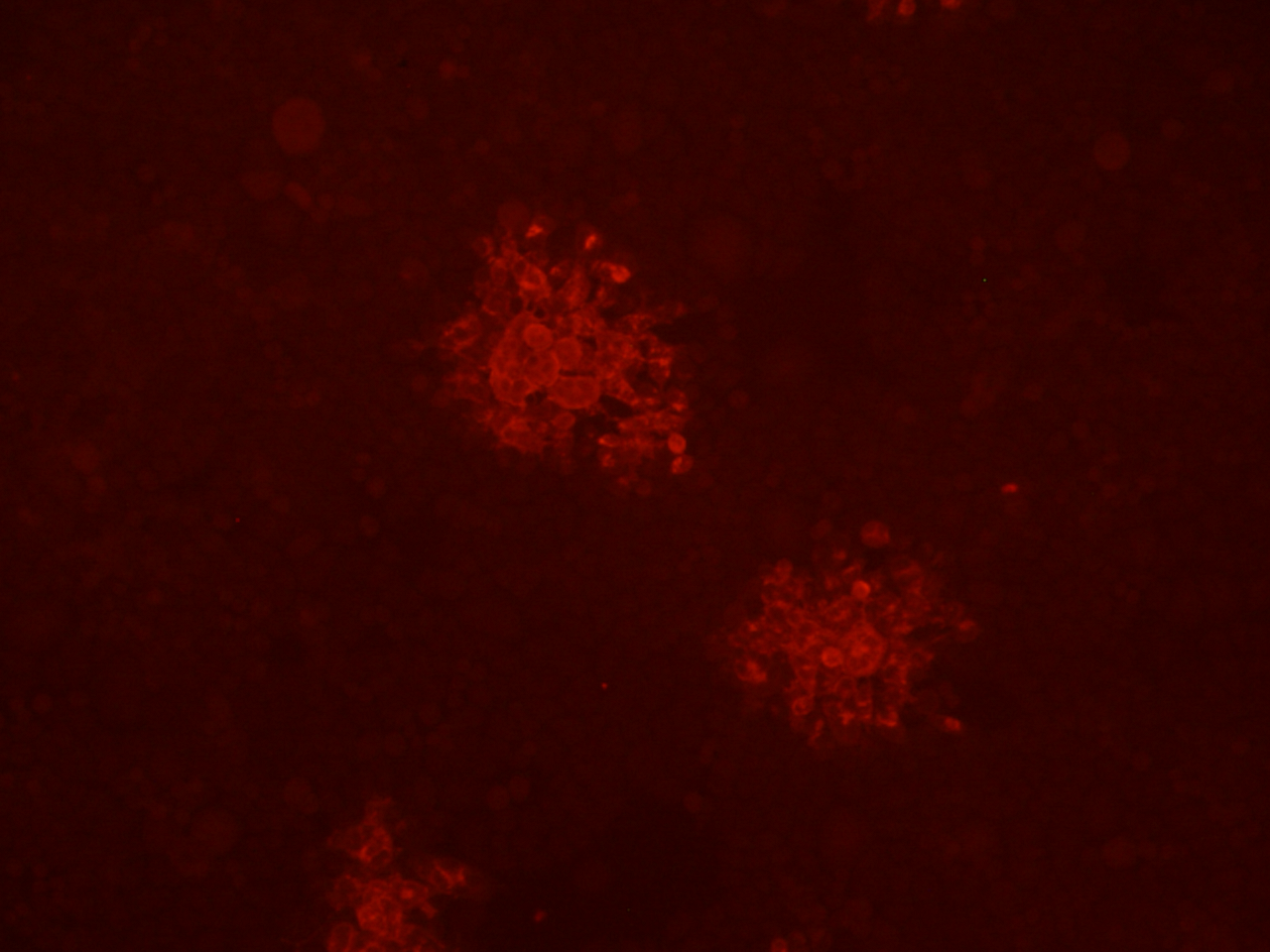

Supplement: S2 File — Level of glycoprotein expression was determined by immunofluorescence assay for the group of IRF1 and its control (pcDNA3), and the group of sh-IRF1 and its control (pSilencer). (ZIP) [file pone.0265925.s001.zip › Repeated experiment Fig 3F_pone.0114021/IRF1/IRF1-1-HSV1 glycoprotein in Fig 3F.tif]

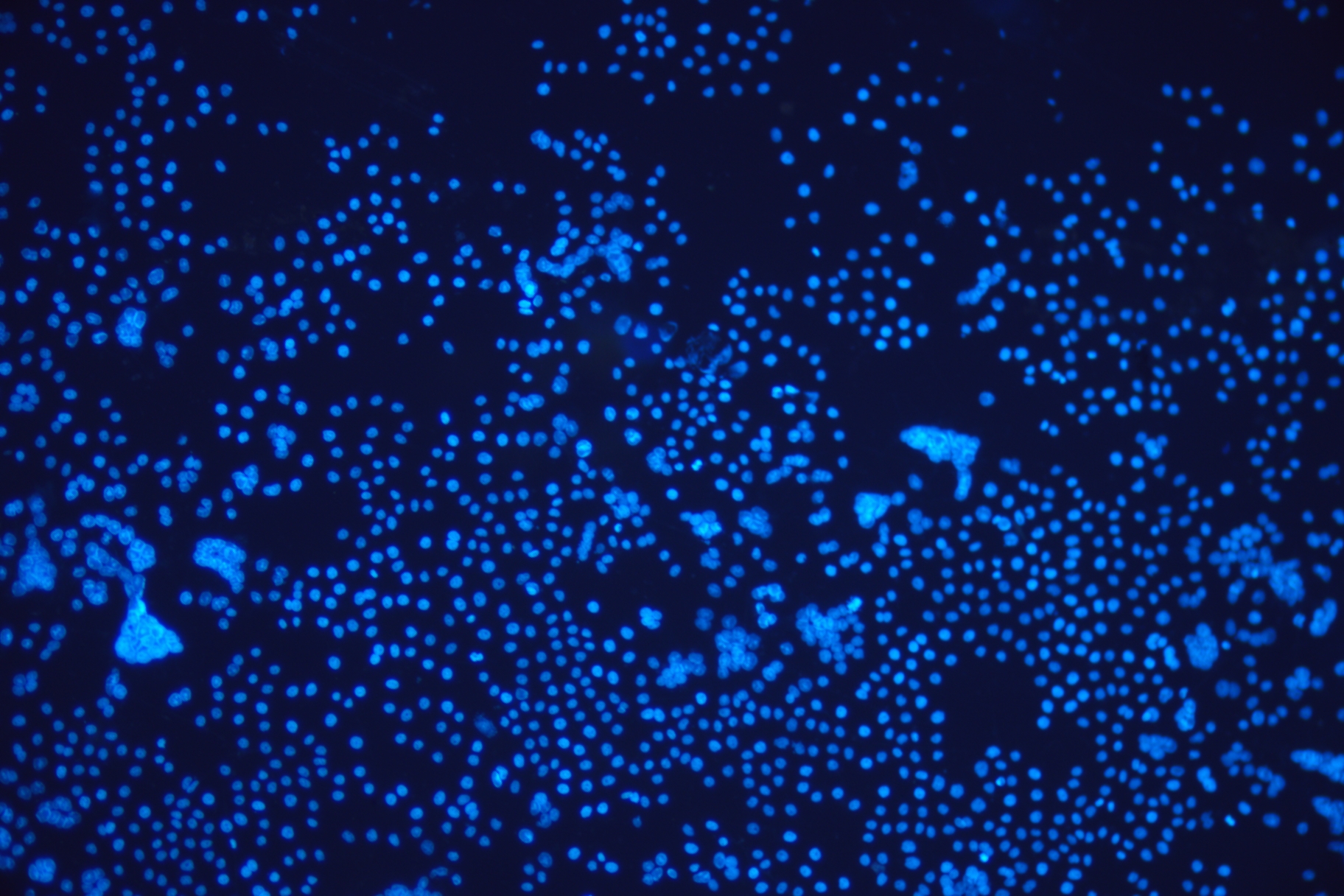

Supplement: S2 File — Level of glycoprotein expression was determined by immunofluorescence assay for the group of IRF1 and its control (pcDNA3), and the group of sh-IRF1 and its control (pSilencer). (ZIP) [file pone.0265925.s001.zip › Repeated experiment Fig 3F_pone.0114021/IRF1/IRF1-2-DAPI.jpg]

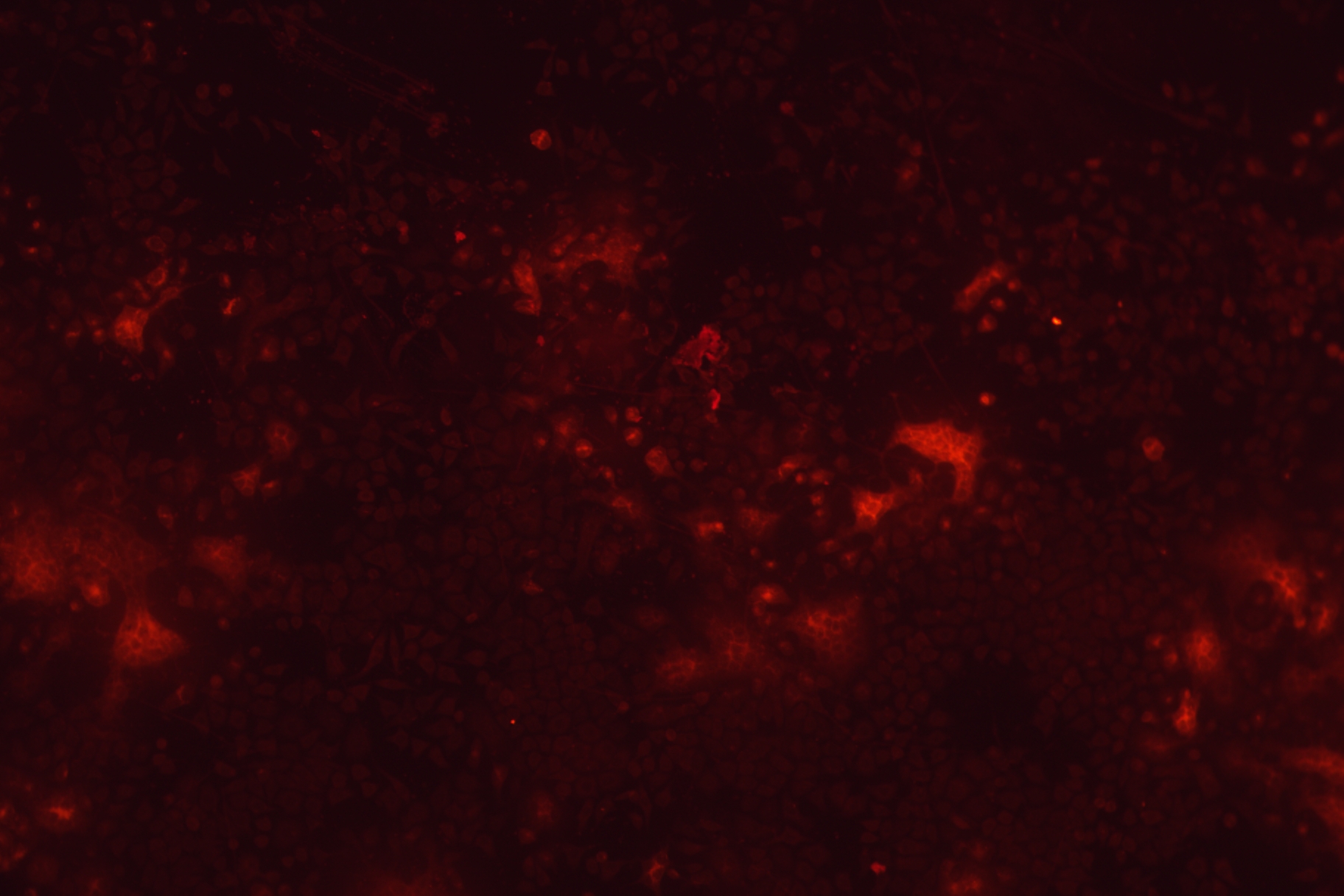

Supplement: S2 File — Level of glycoprotein expression was determined by immunofluorescence assay for the group of IRF1 and its control (pcDNA3), and the group of sh-IRF1 and its control (pSilencer). (ZIP) [file pone.0265925.s001.zip › Repeated experiment Fig 3F_pone.0114021/IRF1/IRF1-2-HSV1 glycoprotein.jpg]

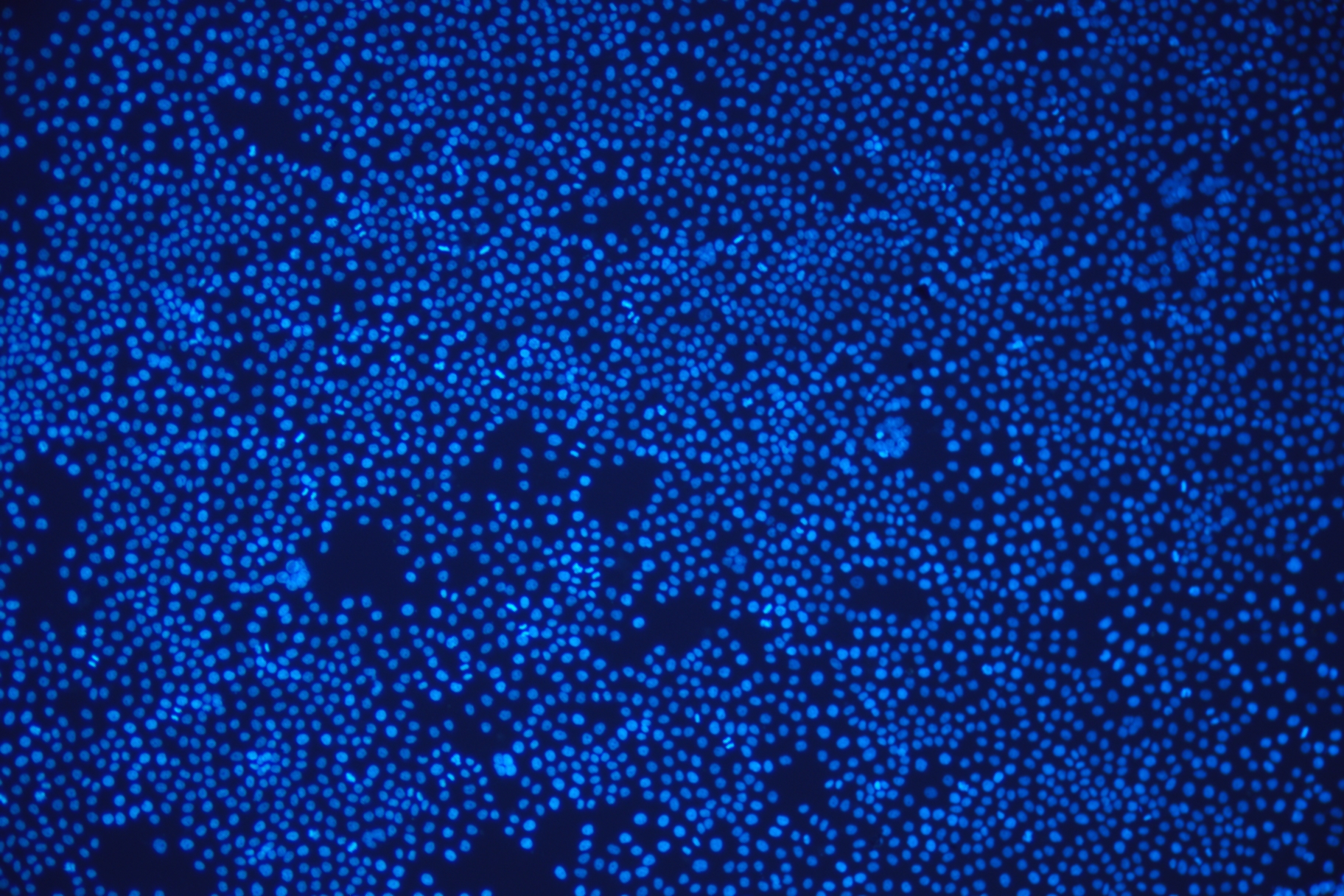

Supplement: S2 File — Level of glycoprotein expression was determined by immunofluorescence assay for the group of IRF1 and its control (pcDNA3), and the group of sh-IRF1 and its control (pSilencer). (ZIP) [file pone.0265925.s001.zip › Repeated experiment Fig 3F_pone.0114021/IRF1/IRF1-3-DAPI.jpg]

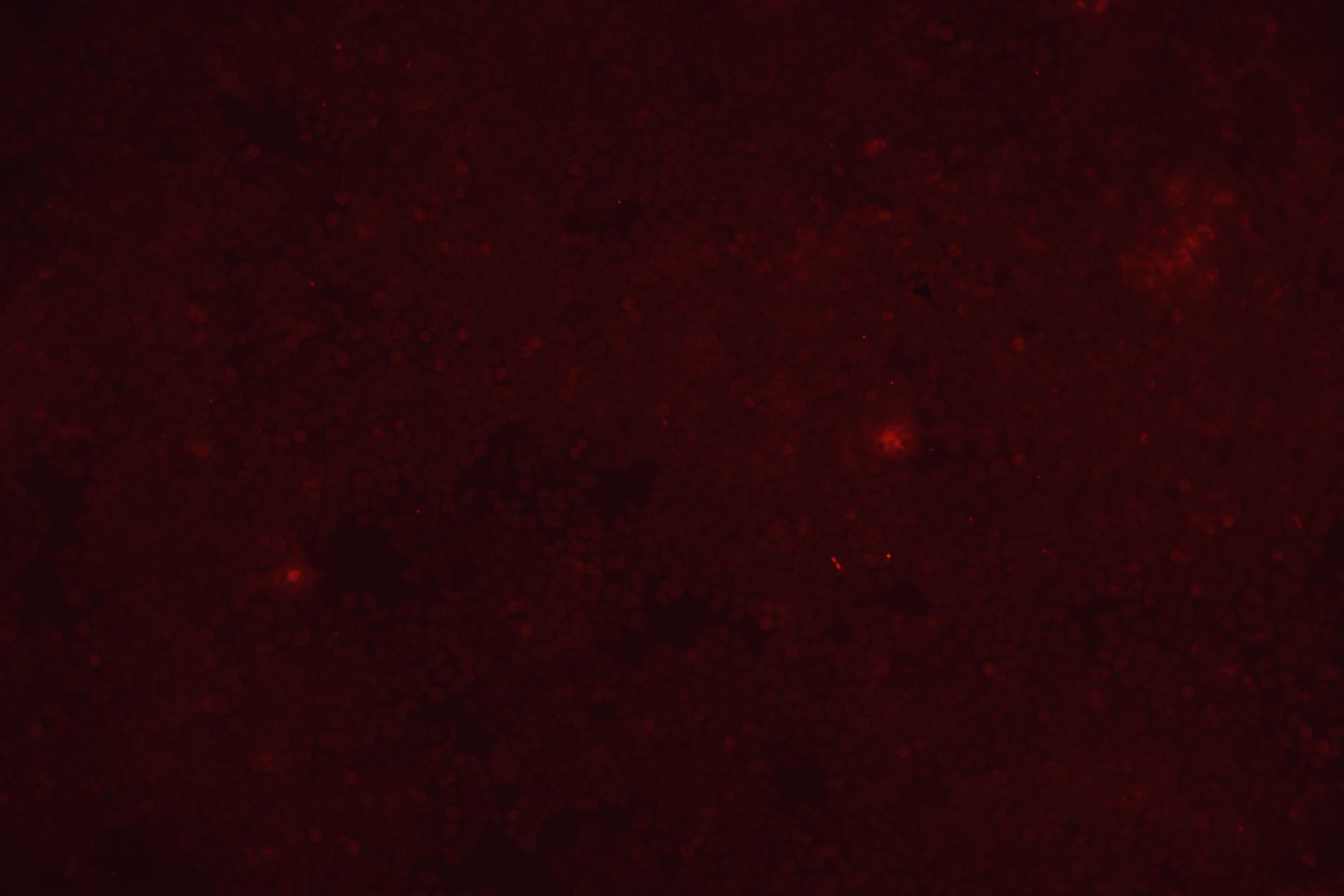

Supplement: S2 File — Level of glycoprotein expression was determined by immunofluorescence assay for the group of IRF1 and its control (pcDNA3), and the group of sh-IRF1 and its control (pSilencer). (ZIP) [file pone.0265925.s001.zip › Repeated experiment Fig 3F_pone.0114021/IRF1/IRF1-3-HSV1 glycoprotein.jpg]

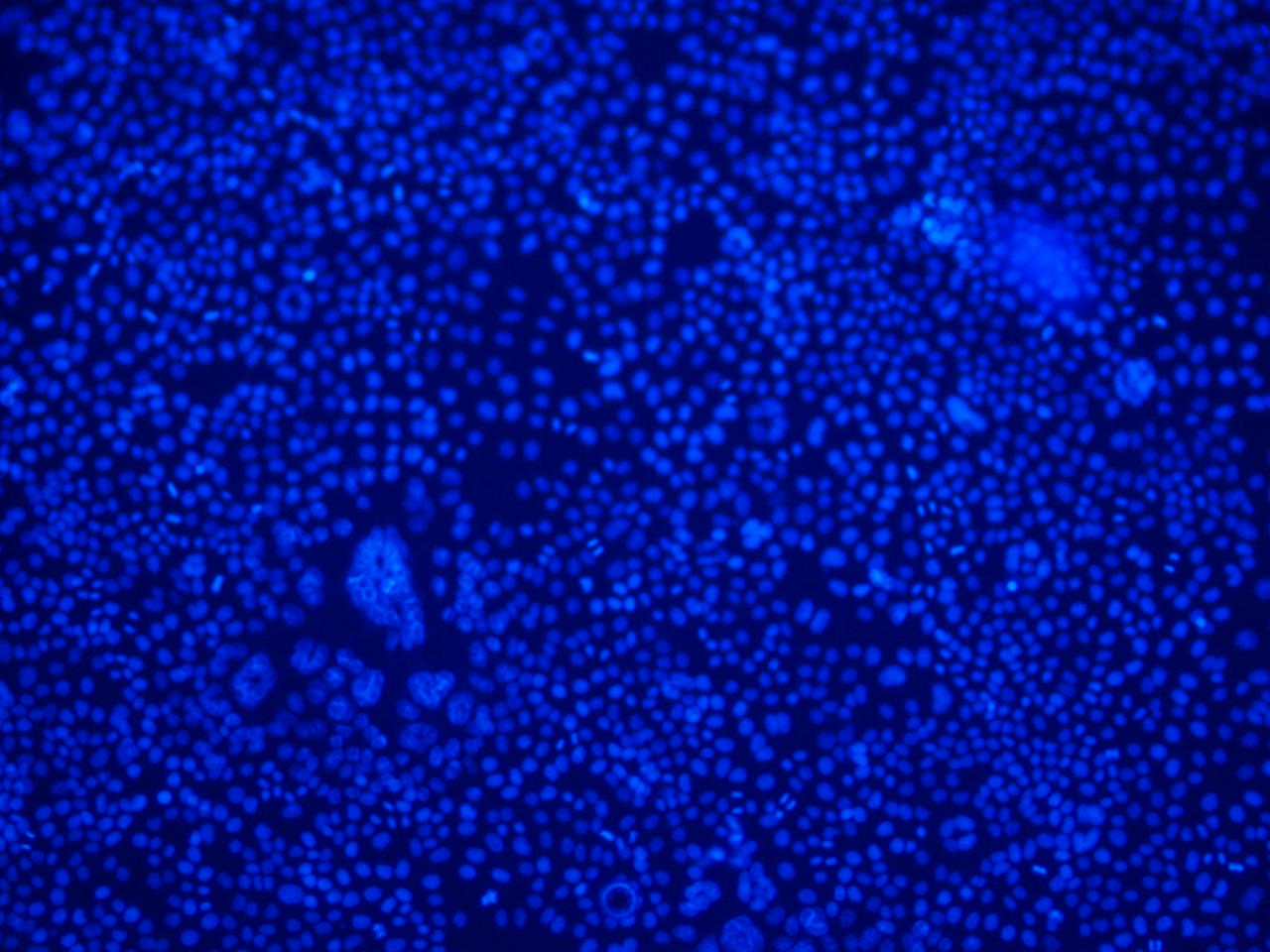

Supplement: S2 File — Level of glycoprotein expression was determined by immunofluorescence assay for the group of IRF1 and its control (pcDNA3), and the group of sh-IRF1 and its control (pSilencer). (ZIP) [file pone.0265925.s001.zip › Repeated experiment Fig 3F_pone.0114021/pcDNA3/pcDNA3-1-DAPI in Fig 3F.tif]

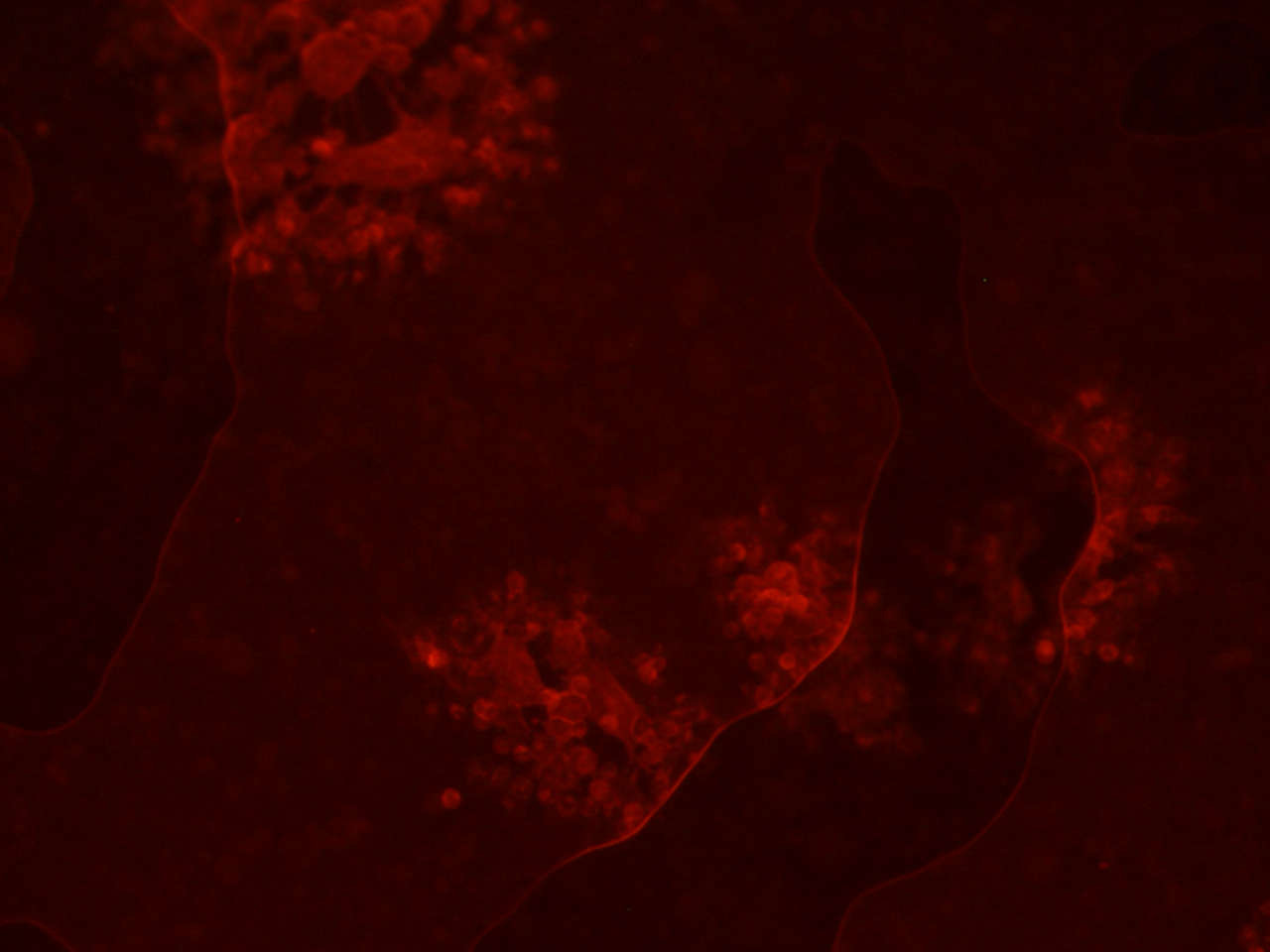

Supplement: S2 File — Level of glycoprotein expression was determined by immunofluorescence assay for the group of IRF1 and its control (pcDNA3), and the group of sh-IRF1 and its control (pSilencer). (ZIP) [file pone.0265925.s001.zip › Repeated experiment Fig 3F_pone.0114021/pcDNA3/pcDNA3-1-HSV1 glycoprotein in Fig 3F.tif]

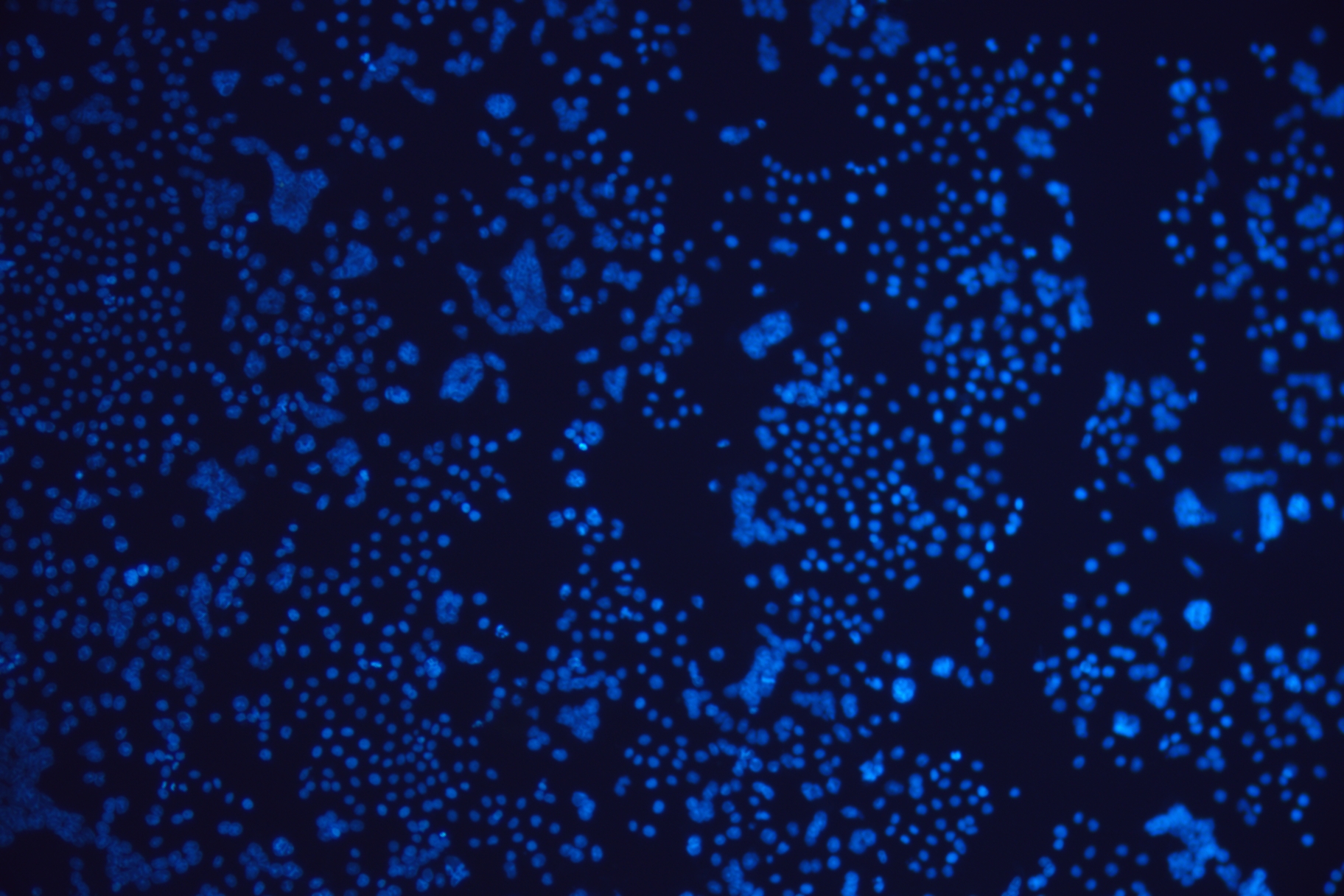

Supplement: S2 File — Level of glycoprotein expression was determined by immunofluorescence assay for the group of IRF1 and its control (pcDNA3), and the group of sh-IRF1 and its control (pSilencer). (ZIP) [file pone.0265925.s001.zip › Repeated experiment Fig 3F_pone.0114021/pcDNA3/pcDNA3-2-DAPI.jpg]

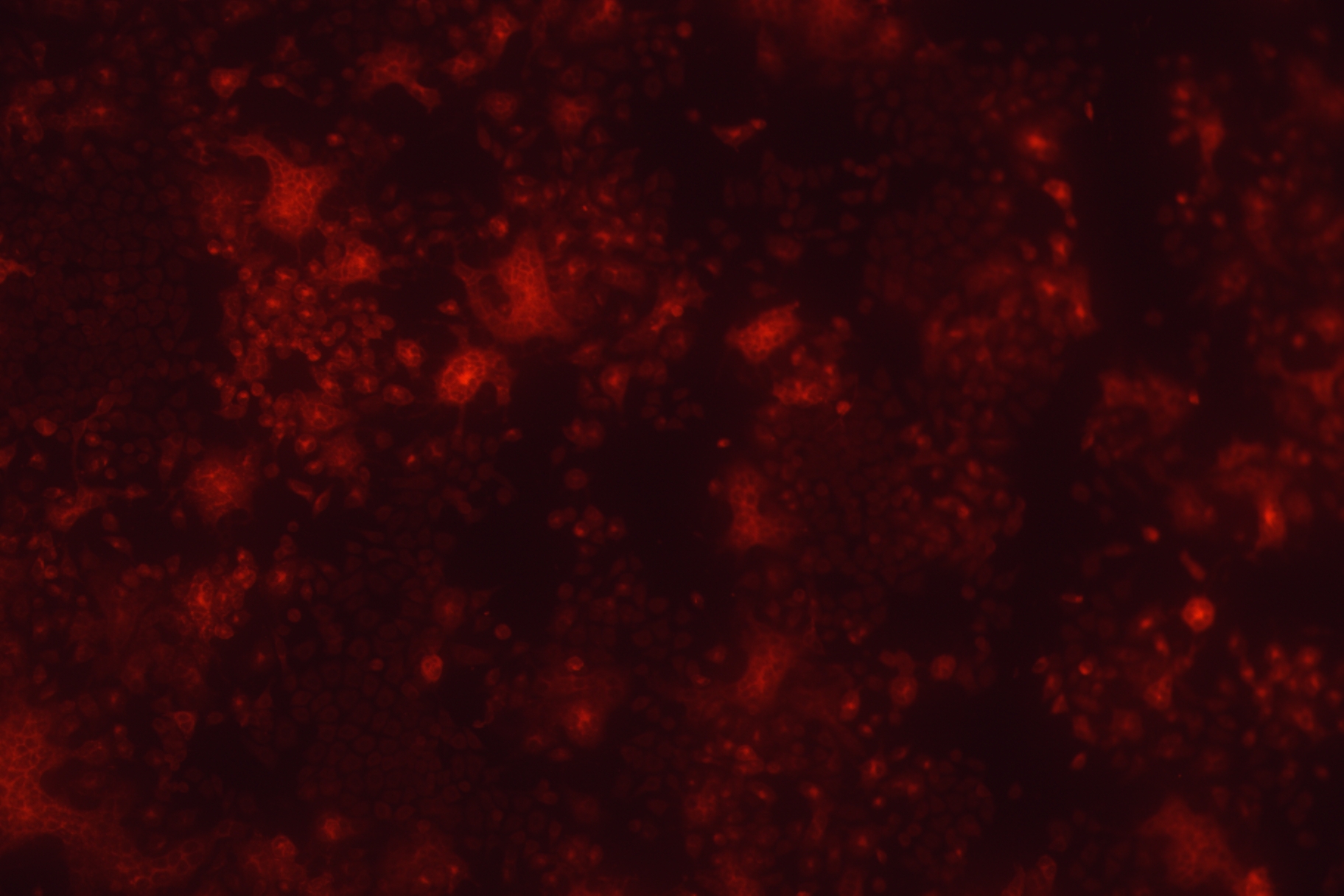

Supplement: S2 File — Level of glycoprotein expression was determined by immunofluorescence assay for the group of IRF1 and its control (pcDNA3), and the group of sh-IRF1 and its control (pSilencer). (ZIP) [file pone.0265925.s001.zip › Repeated experiment Fig 3F_pone.0114021/pcDNA3/pcDNA3-2-HSV1 glycoprotein.jpg]

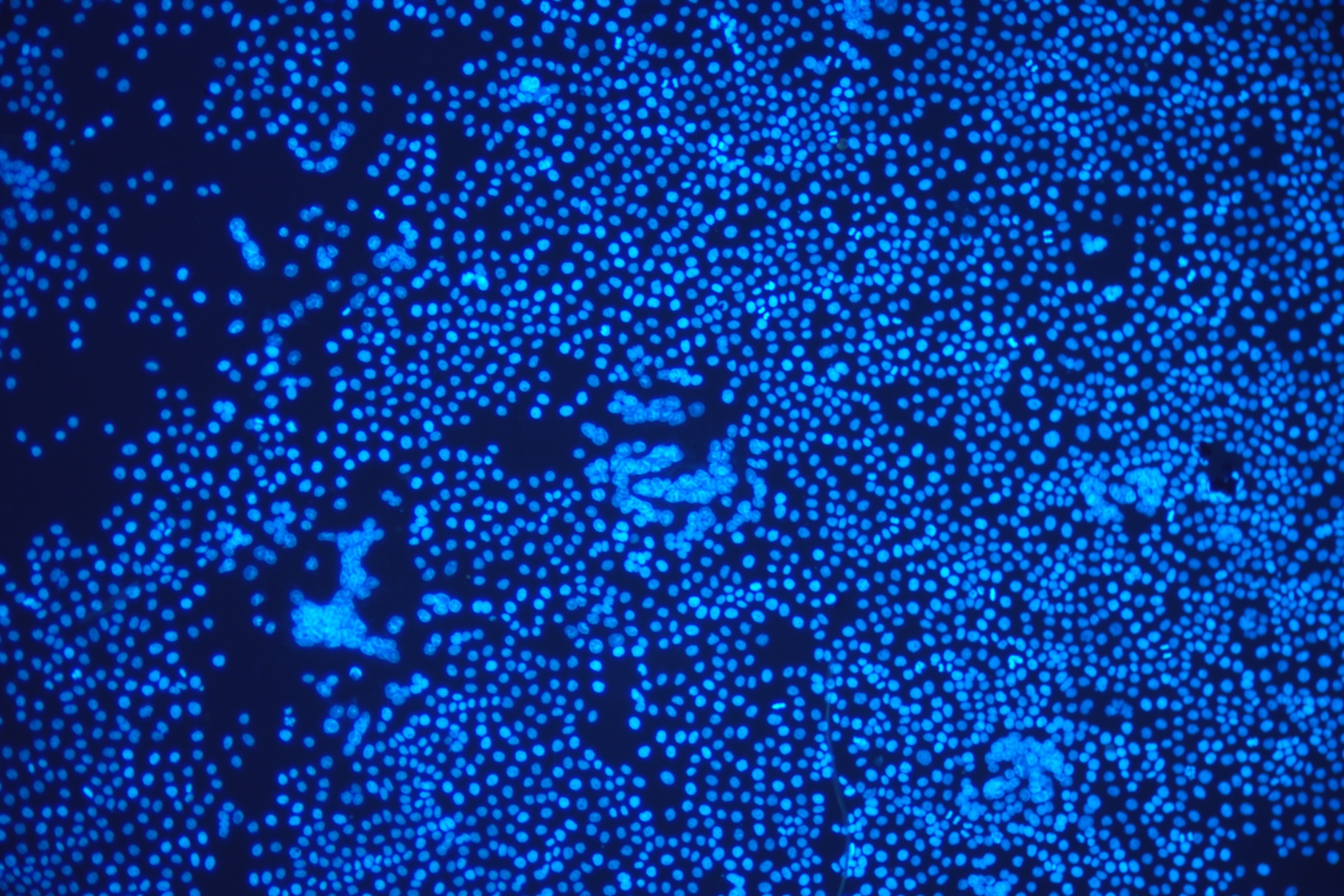

Supplement: S2 File — Level of glycoprotein expression was determined by immunofluorescence assay for the group of IRF1 and its control (pcDNA3), and the group of sh-IRF1 and its control (pSilencer). (ZIP) [file pone.0265925.s001.zip › Repeated experiment Fig 3F_pone.0114021/pcDNA3/pcDNA3-3-DAPI.jpg]

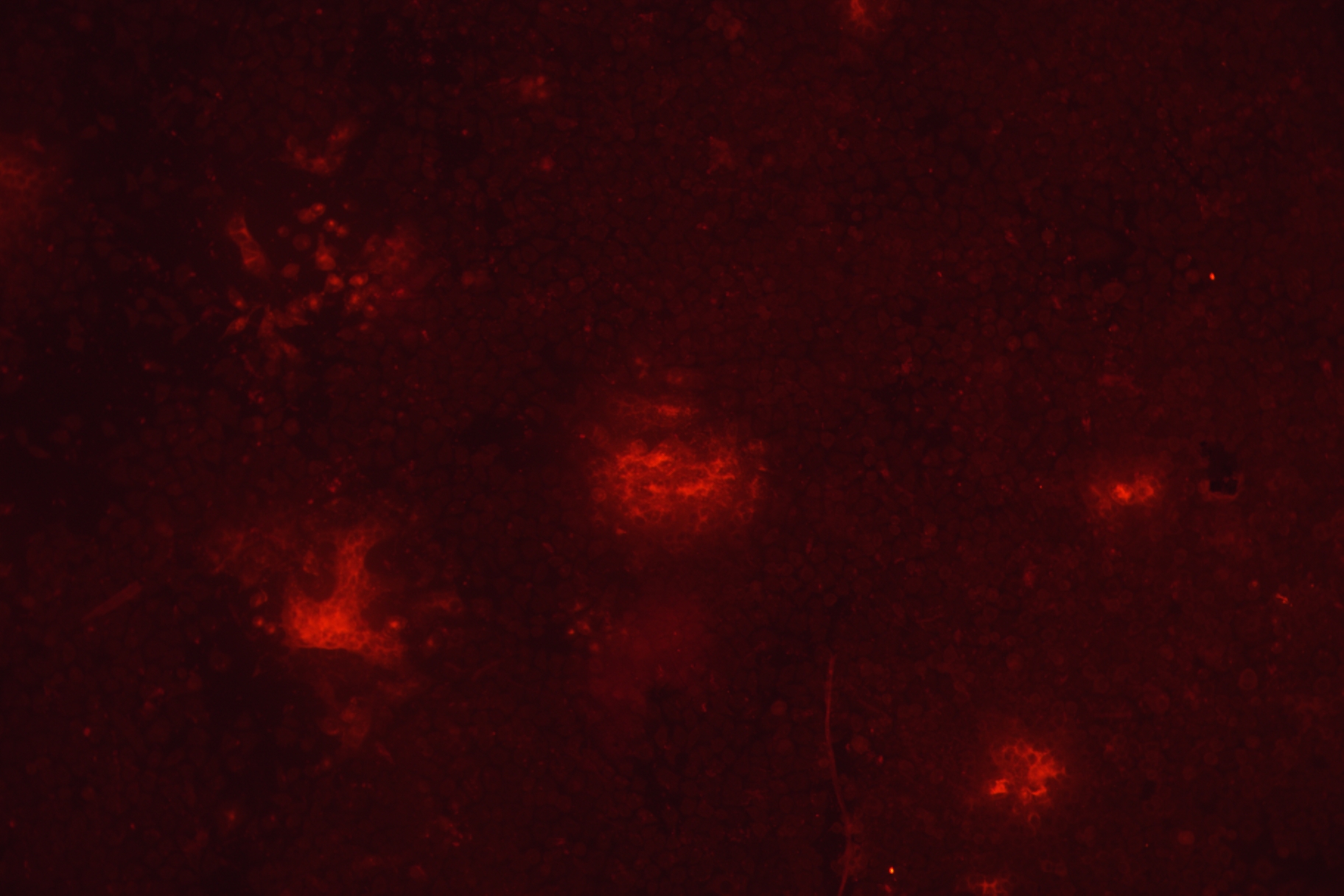

Supplement: S2 File — Level of glycoprotein expression was determined by immunofluorescence assay for the group of IRF1 and its control (pcDNA3), and the group of sh-IRF1 and its control (pSilencer). (ZIP) [file pone.0265925.s001.zip › Repeated experiment Fig 3F_pone.0114021/pcDNA3/pcDNA3-3-HSV1 glycoprotein.jpg]

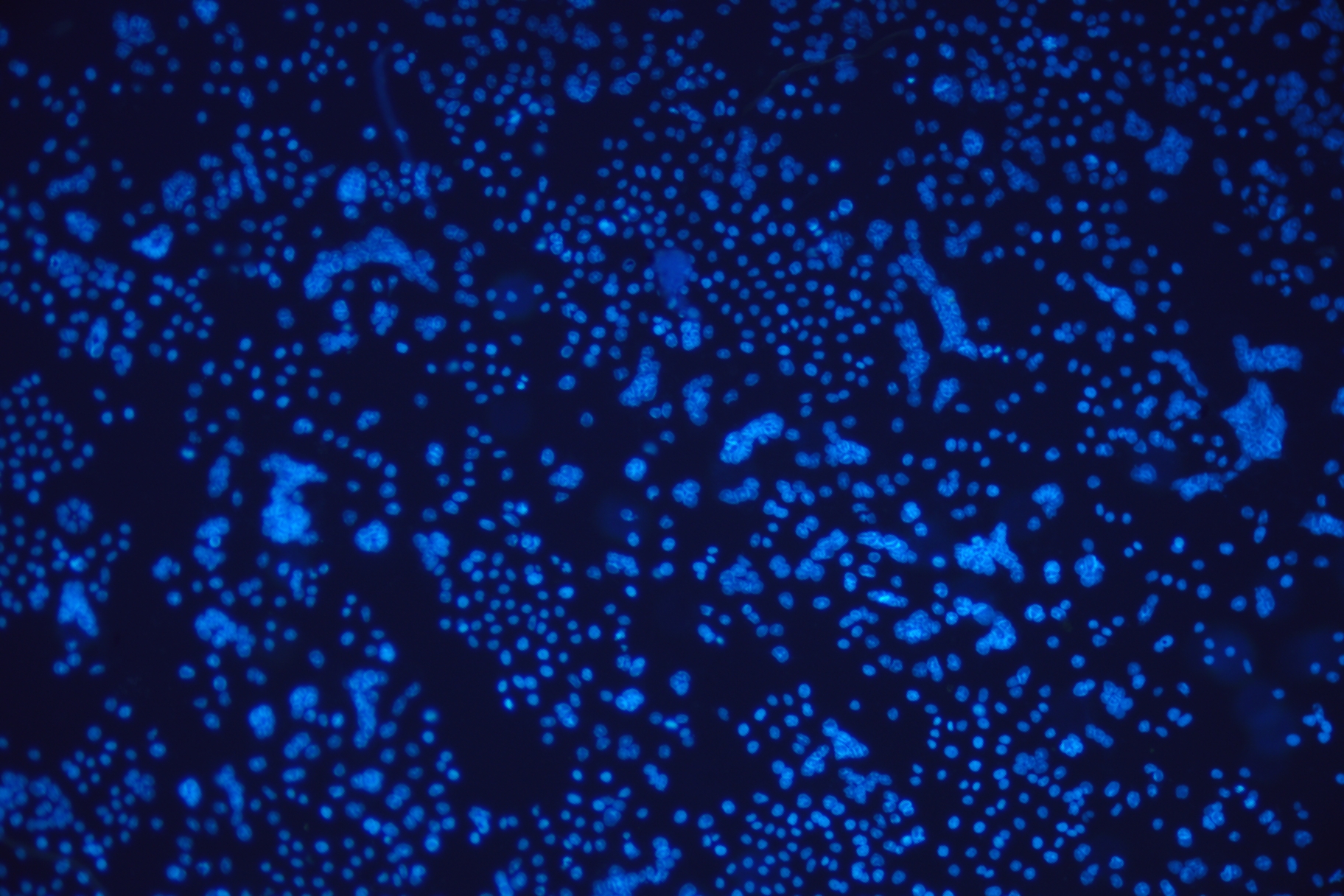

Supplement: S2 File — Level of glycoprotein expression was determined by immunofluorescence assay for the group of IRF1 and its control (pcDNA3), and the group of sh-IRF1 and its control (pSilencer). (ZIP) [file pone.0265925.s001.zip › Repeated experiment Fig 3F_pone.0114021/psilencer/pSilencer-2-DAPI.jpg]

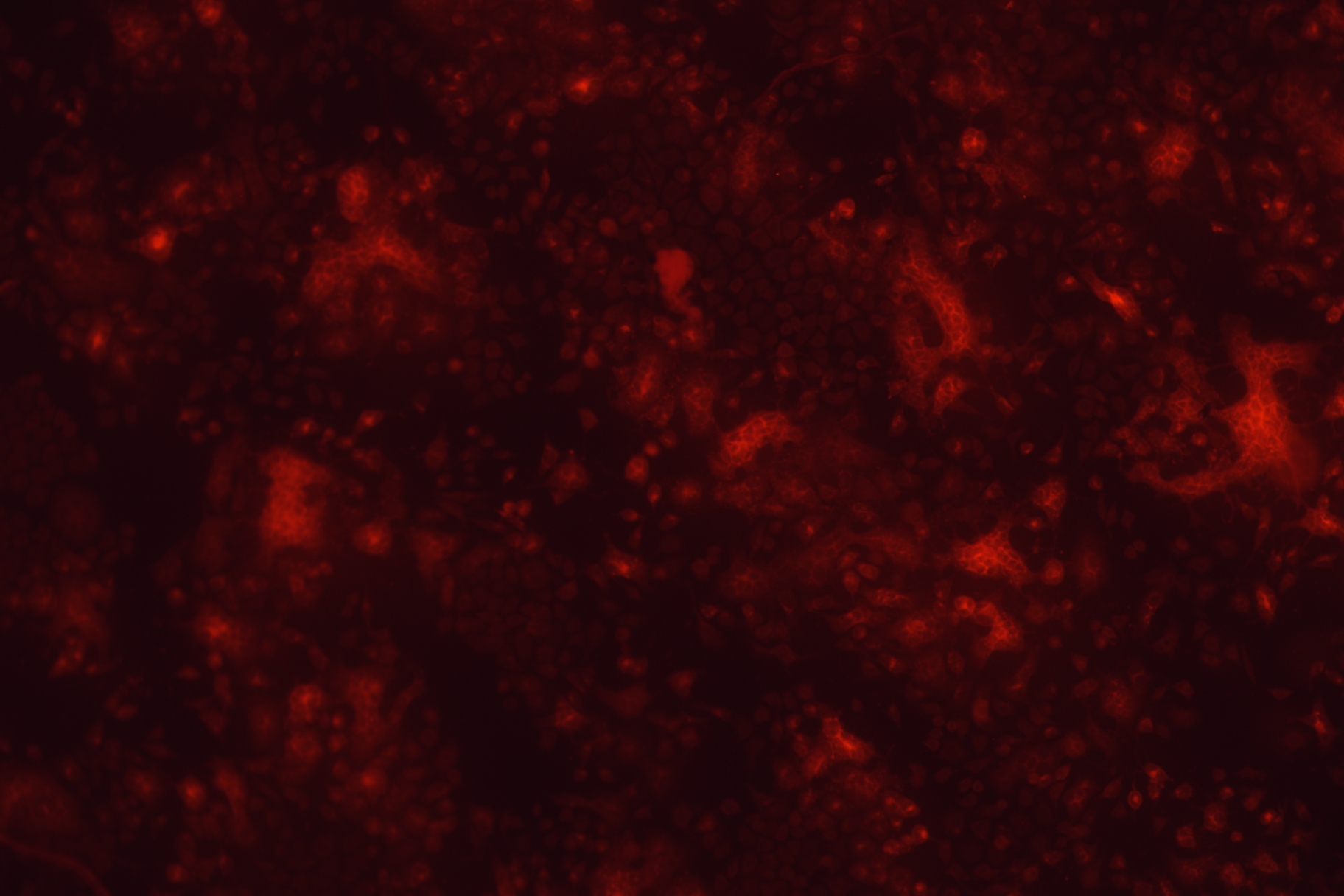

Supplement: S2 File — Level of glycoprotein expression was determined by immunofluorescence assay for the group of IRF1 and its control (pcDNA3), and the group of sh-IRF1 and its control (pSilencer). (ZIP) [file pone.0265925.s001.zip › Repeated experiment Fig 3F_pone.0114021/psilencer/pSilencer-2-HSV1 glycoprotein.jpg]

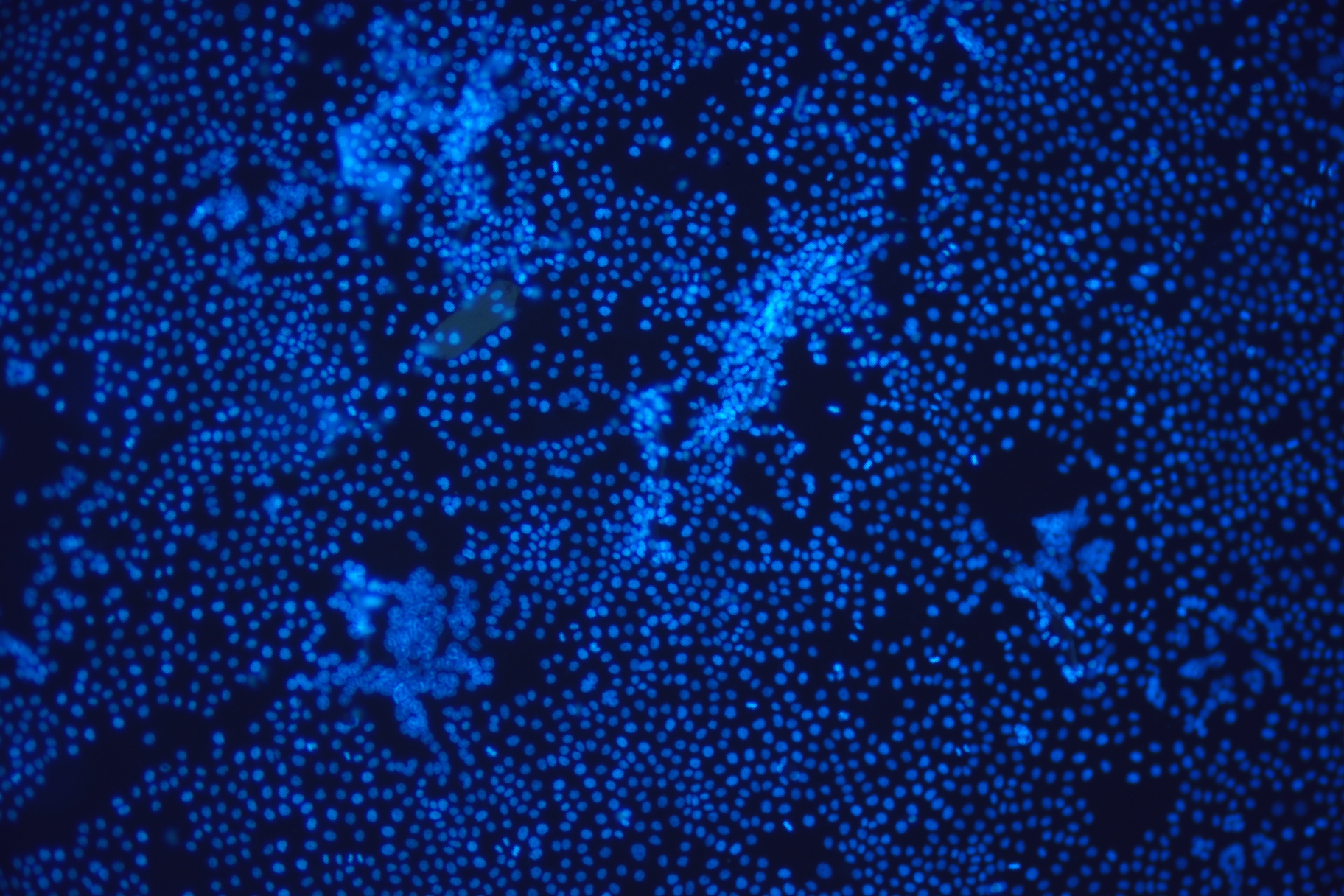

Supplement: S2 File — Level of glycoprotein expression was determined by immunofluorescence assay for the group of IRF1 and its control (pcDNA3), and the group of sh-IRF1 and its control (pSilencer). (ZIP) [file pone.0265925.s001.zip › Repeated experiment Fig 3F_pone.0114021/psilencer/pSilencer-3-DAPI.jpg]

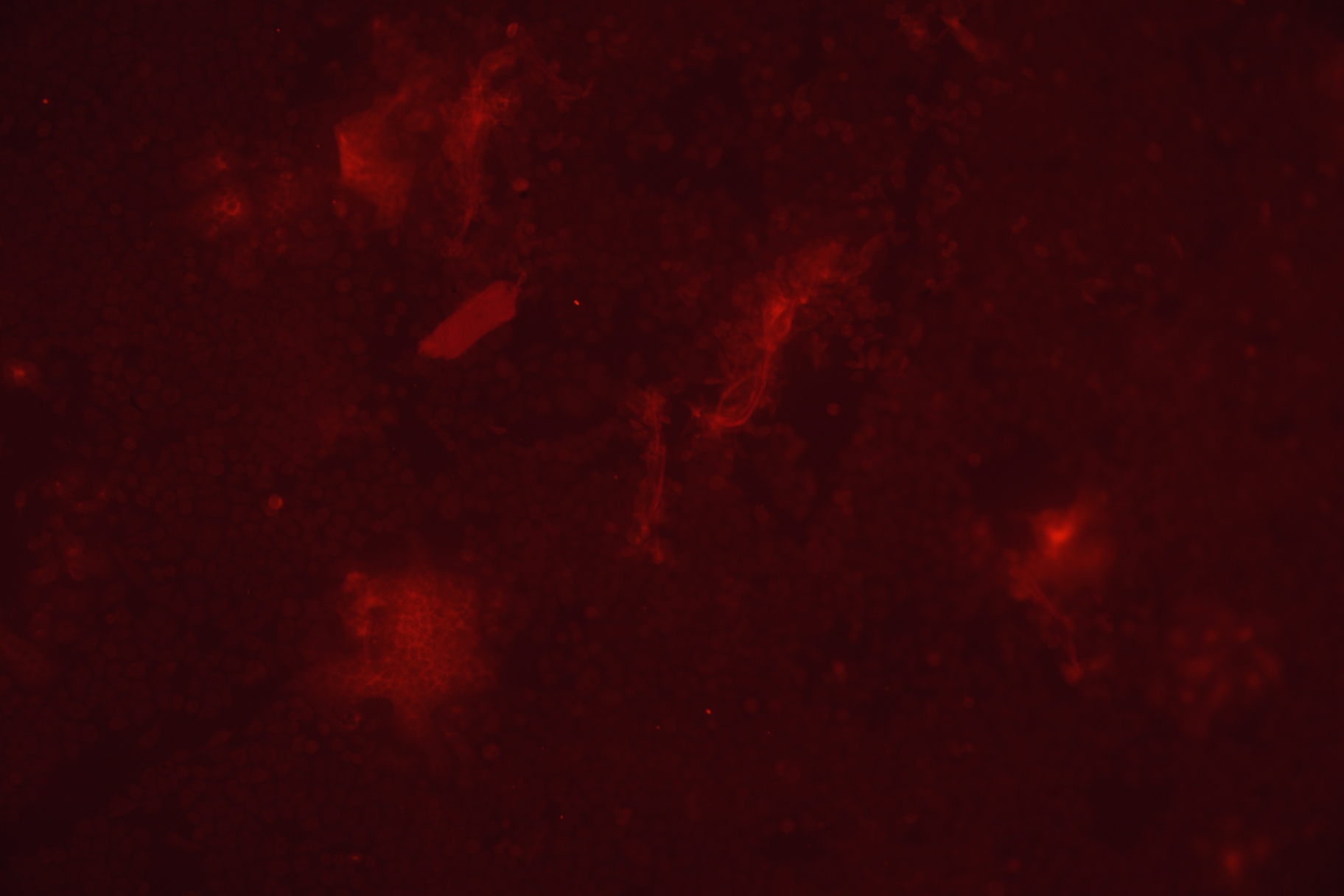

Supplement: S2 File — Level of glycoprotein expression was determined by immunofluorescence assay for the group of IRF1 and its control (pcDNA3), and the group of sh-IRF1 and its control (pSilencer). (ZIP) [file pone.0265925.s001.zip › Repeated experiment Fig 3F_pone.0114021/psilencer/pSilencer-3-HSV1 glycoprotein.jpg]

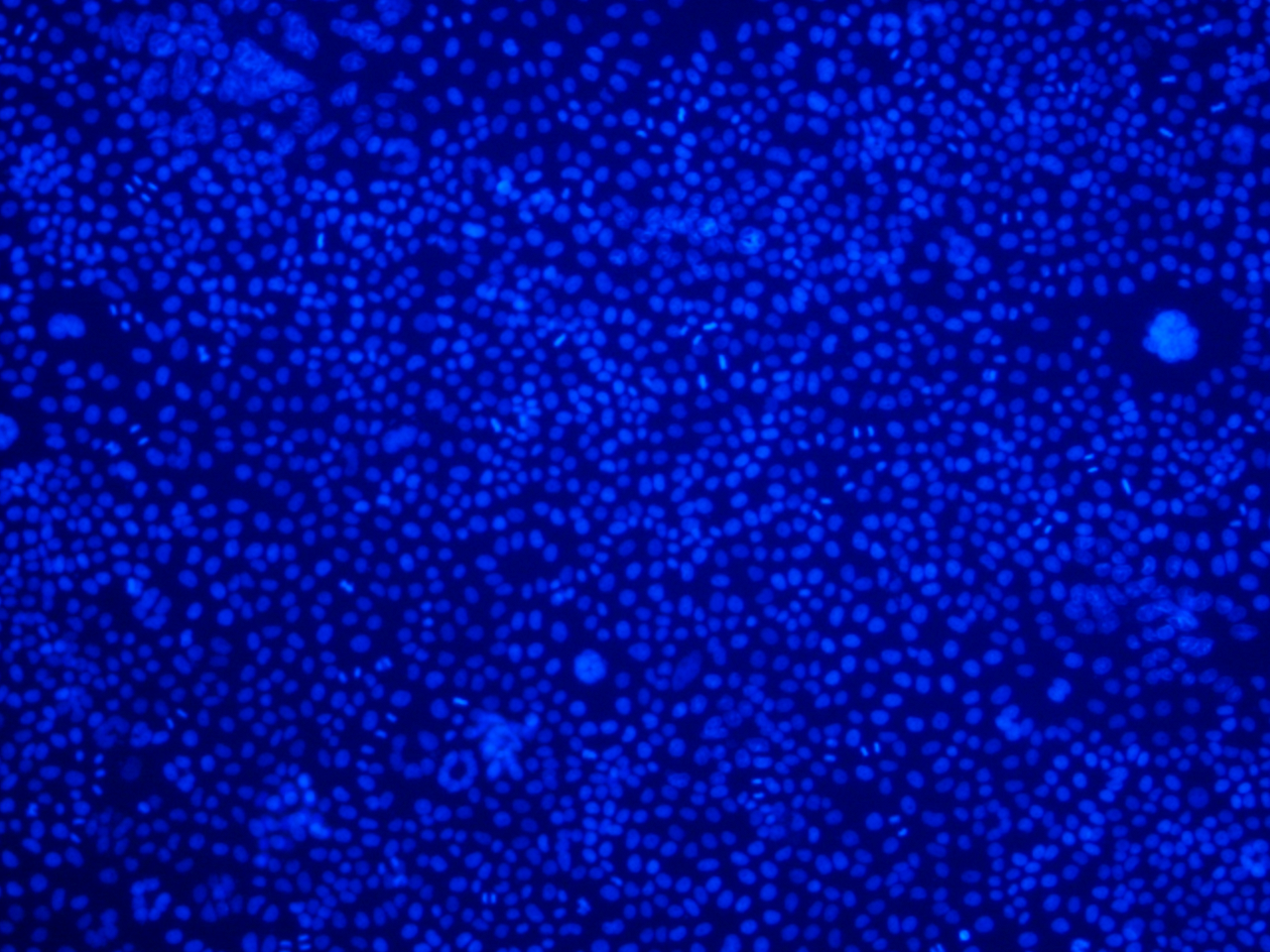

Supplement: S2 File — Level of glycoprotein expression was determined by immunofluorescence assay for the group of IRF1 and its control (pcDNA3), and the group of sh-IRF1 and its control (pSilencer). (ZIP) [file pone.0265925.s001.zip › Repeated experiment Fig 3F_pone.0114021/psilencer/psilencer-1-DAPI in Fig 3F.tif]

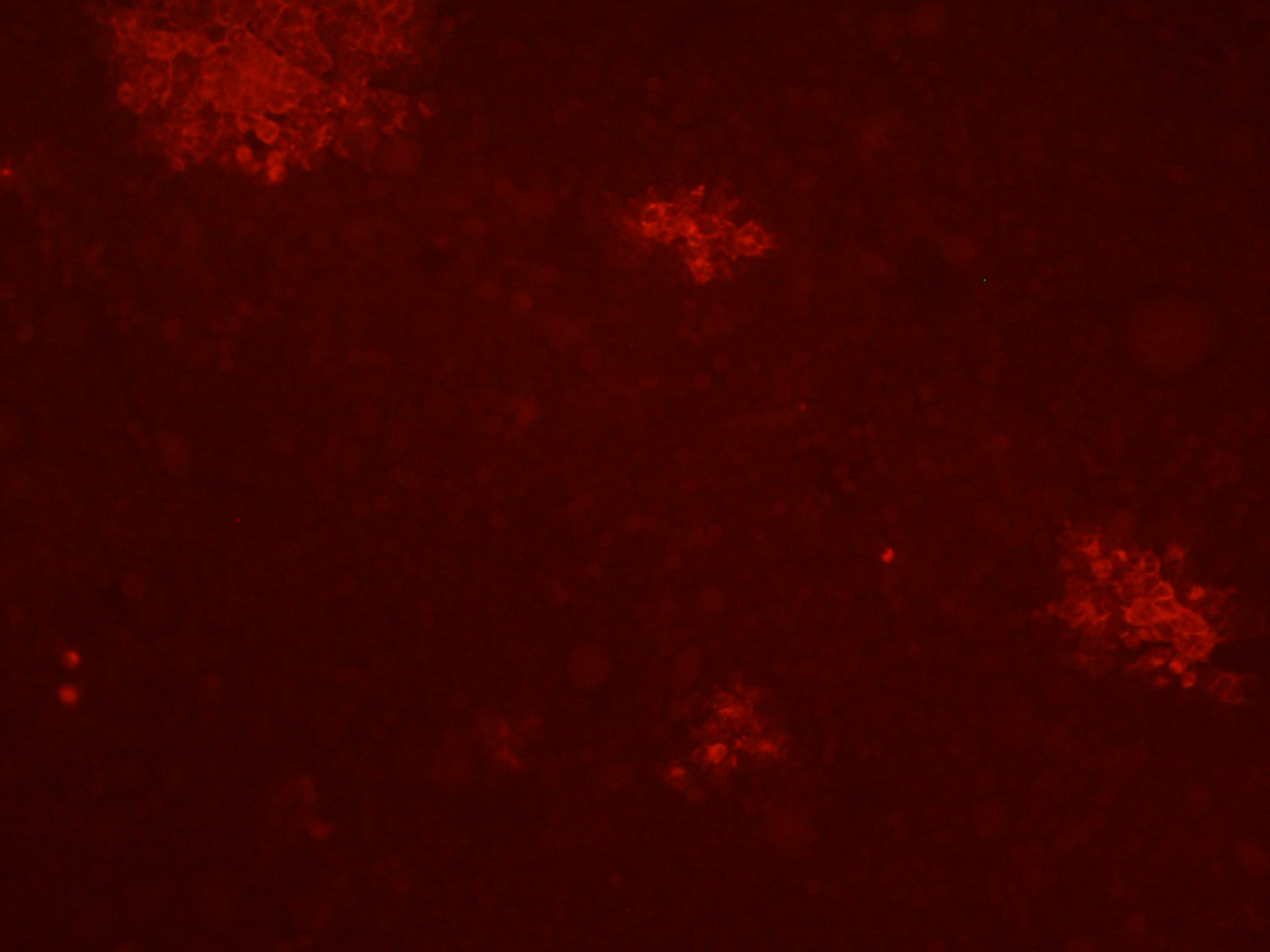

Supplement: S2 File — Level of glycoprotein expression was determined by immunofluorescence assay for the group of IRF1 and its control (pcDNA3), and the group of sh-IRF1 and its control (pSilencer). (ZIP) [file pone.0265925.s001.zip › Repeated experiment Fig 3F_pone.0114021/psilencer/psilencer-1-HSV1 in Fig 3Fglycoprotein.tif]

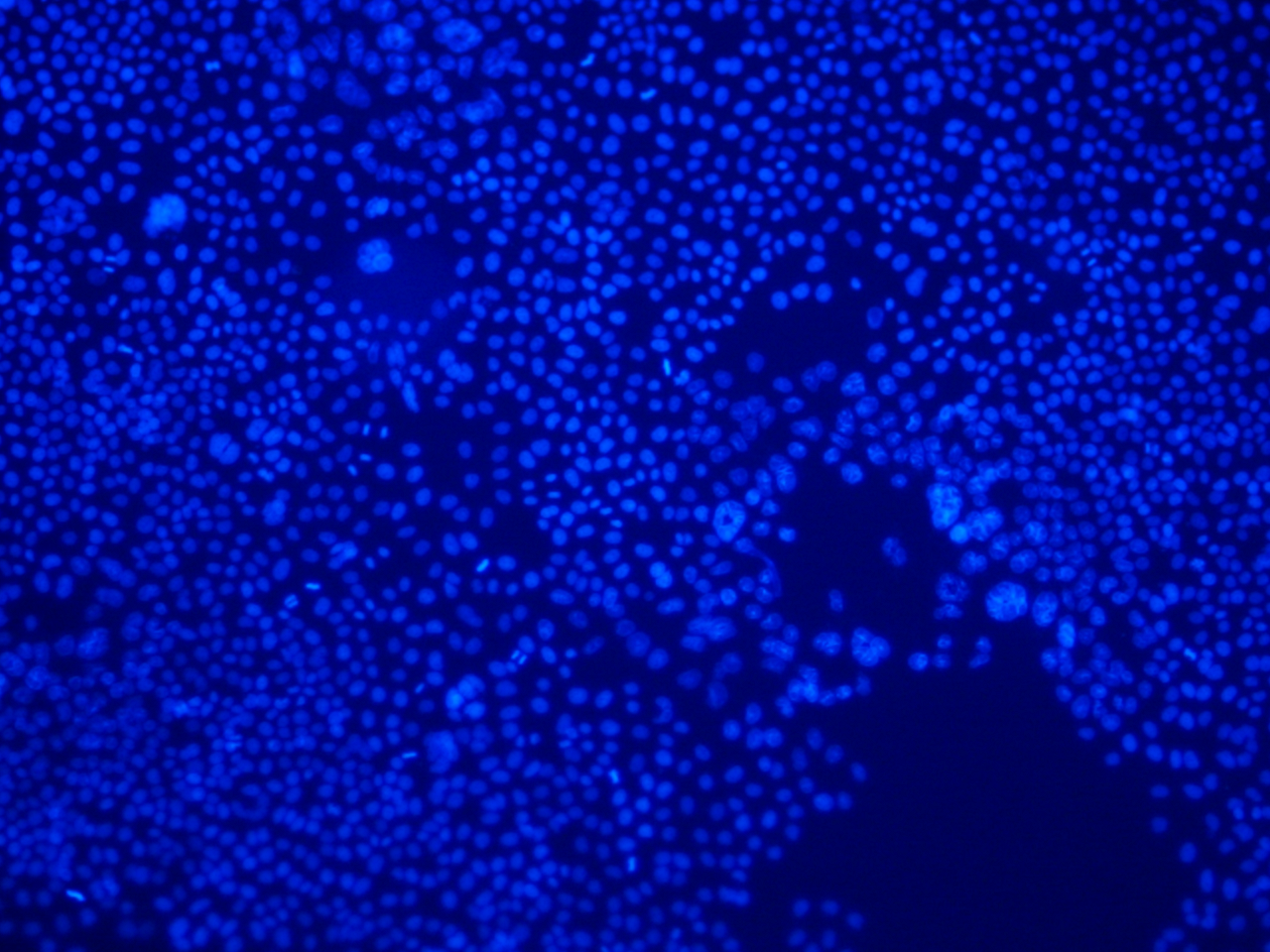

Supplement: S2 File — Level of glycoprotein expression was determined by immunofluorescence assay for the group of IRF1 and its control (pcDNA3), and the group of sh-IRF1 and its control (pSilencer). (ZIP) [file pone.0265925.s001.zip › Repeated experiment Fig 3F_pone.0114021/sh-IRF1/shIRF1-1-DAPI in Fig 3F.tif]

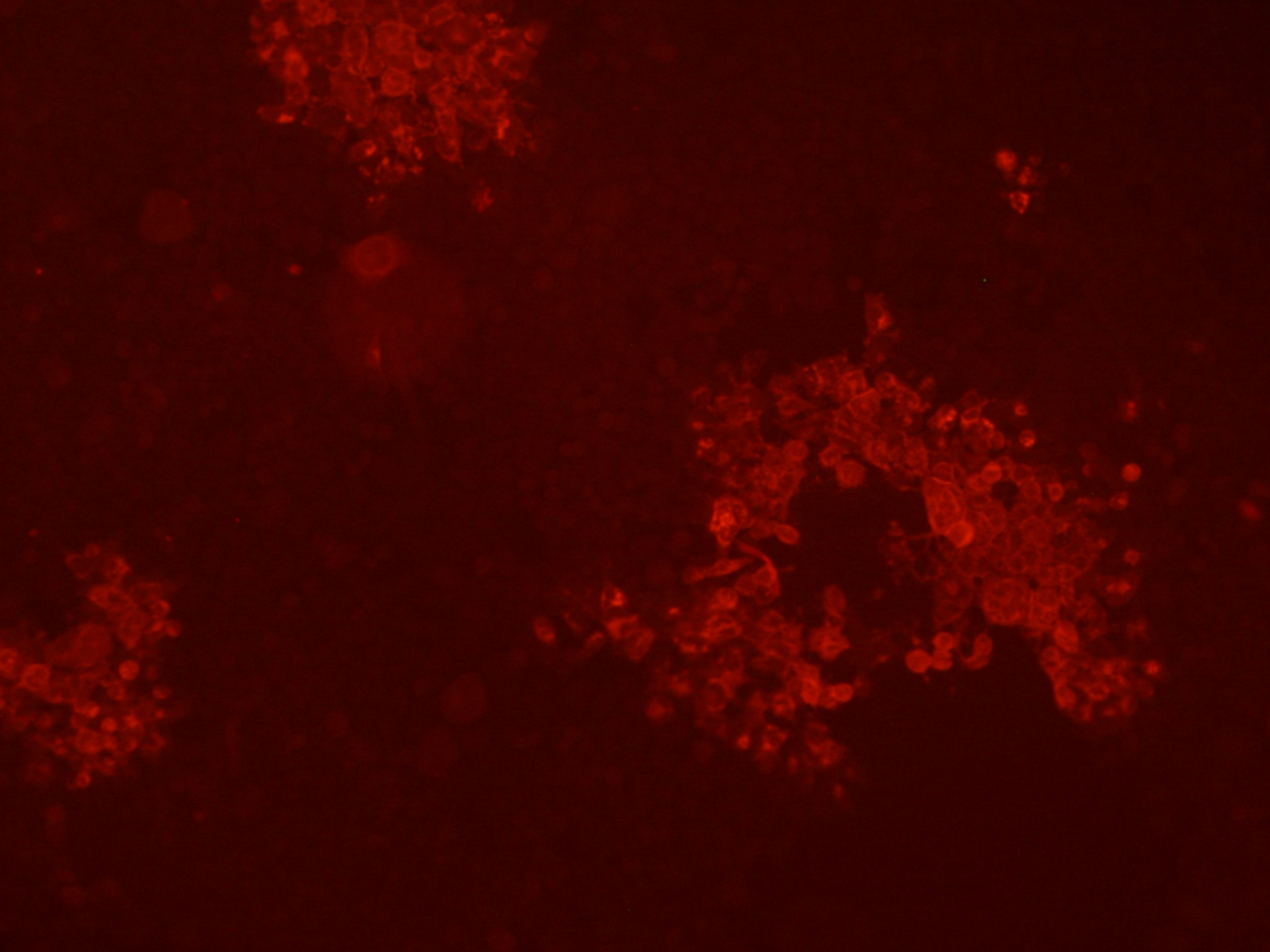

Supplement: S2 File — Level of glycoprotein expression was determined by immunofluorescence assay for the group of IRF1 and its control (pcDNA3), and the group of sh-IRF1 and its control (pSilencer). (ZIP) [file pone.0265925.s001.zip › Repeated experiment Fig 3F_pone.0114021/sh-IRF1/shIRF1-1-HSV1 glycoprotein in Fig 3F.tif]

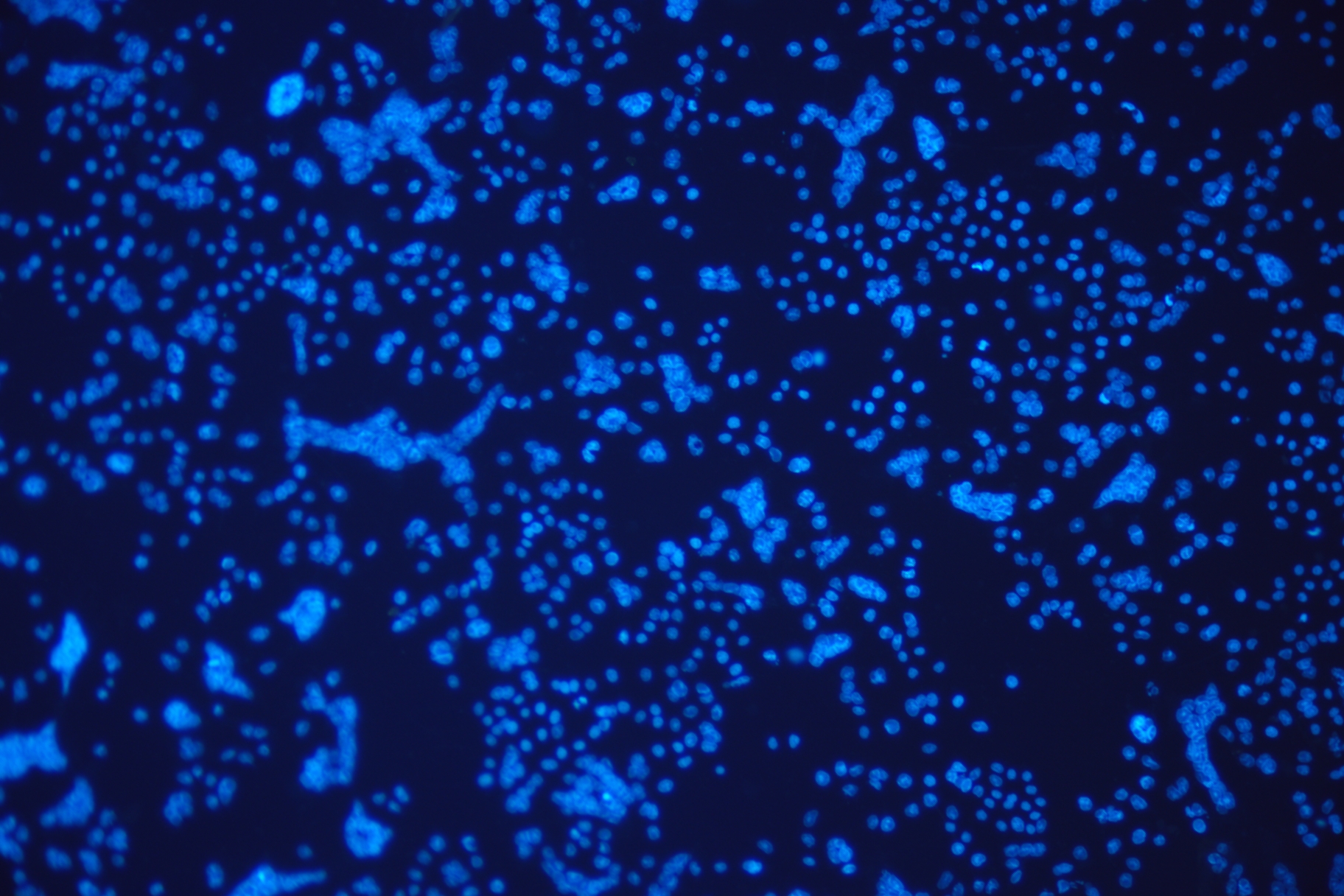

Supplement: S2 File — Level of glycoprotein expression was determined by immunofluorescence assay for the group of IRF1 and its control (pcDNA3), and the group of sh-IRF1 and its control (pSilencer). (ZIP) [file pone.0265925.s001.zip › Repeated experiment Fig 3F_pone.0114021/sh-IRF1/shIRF1-2-DAPI.jpg]

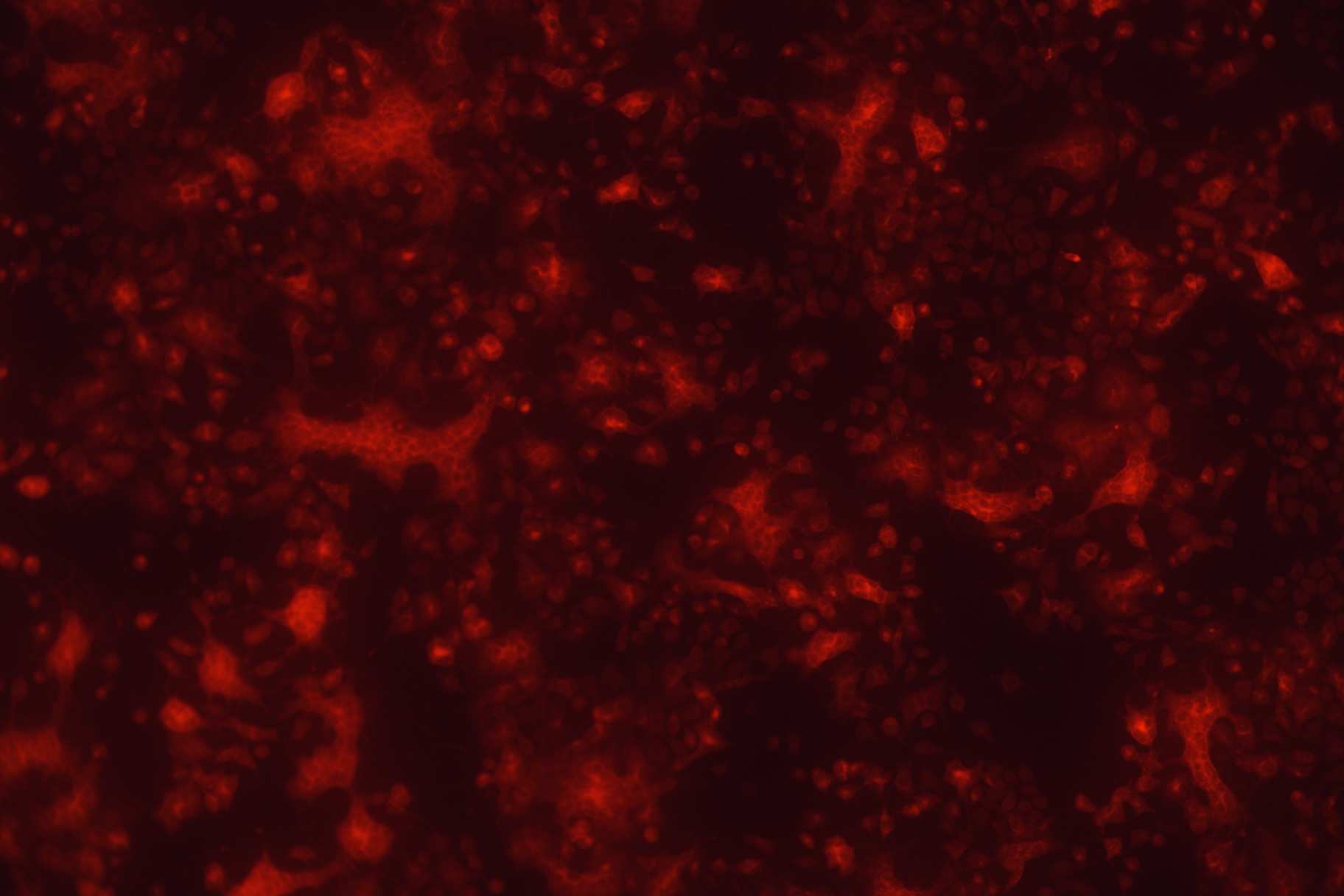

Supplement: S2 File — Level of glycoprotein expression was determined by immunofluorescence assay for the group of IRF1 and its control (pcDNA3), and the group of sh-IRF1 and its control (pSilencer). (ZIP) [file pone.0265925.s001.zip › Repeated experiment Fig 3F_pone.0114021/sh-IRF1/shIRF1-2-HSV1 glycoprotein.jpg]

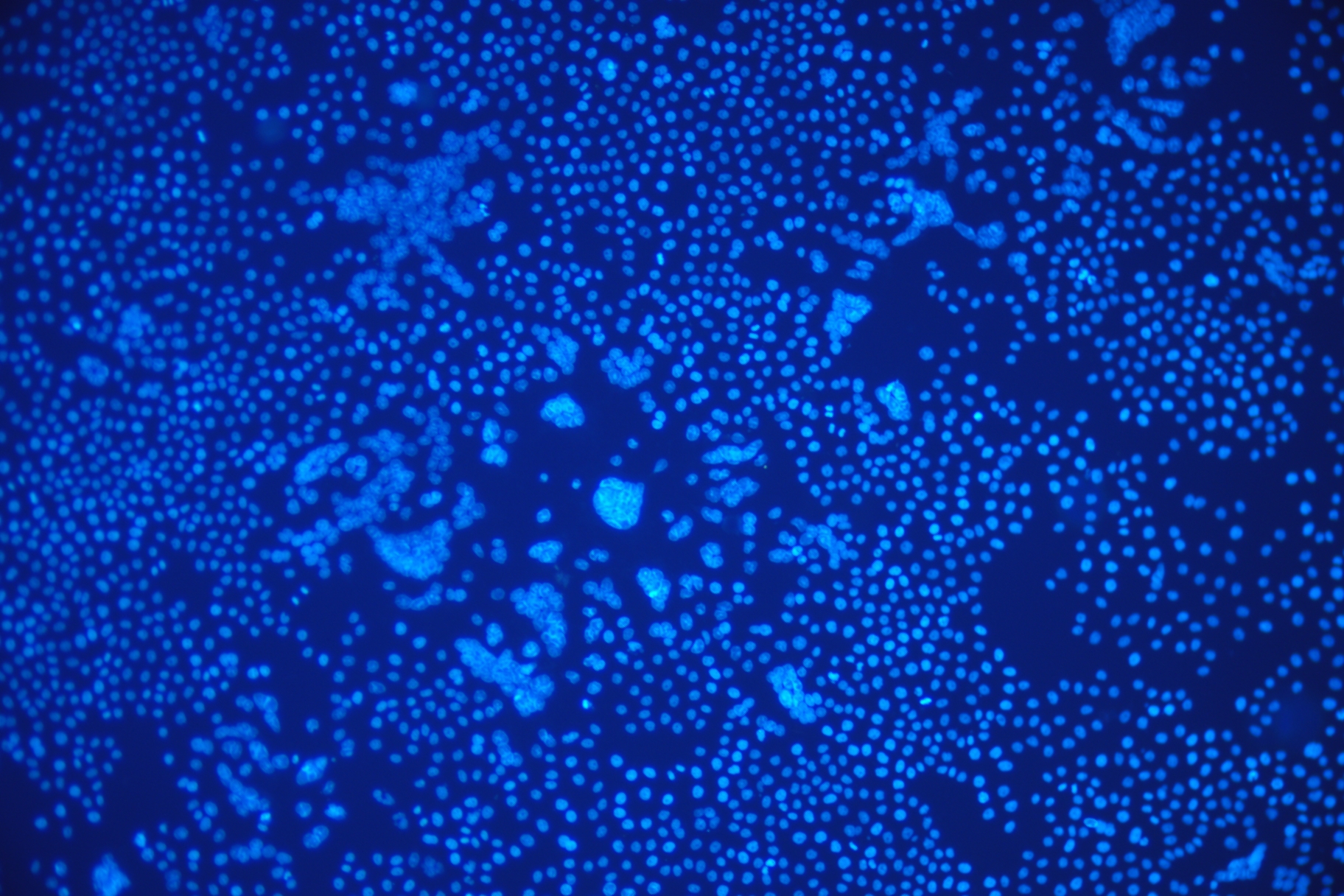

Supplement: S2 File — Level of glycoprotein expression was determined by immunofluorescence assay for the group of IRF1 and its control (pcDNA3), and the group of sh-IRF1 and its control (pSilencer). (ZIP) [file pone.0265925.s001.zip › Repeated experiment Fig 3F_pone.0114021/sh-IRF1/shIRF1-3-DAPI.jpg]

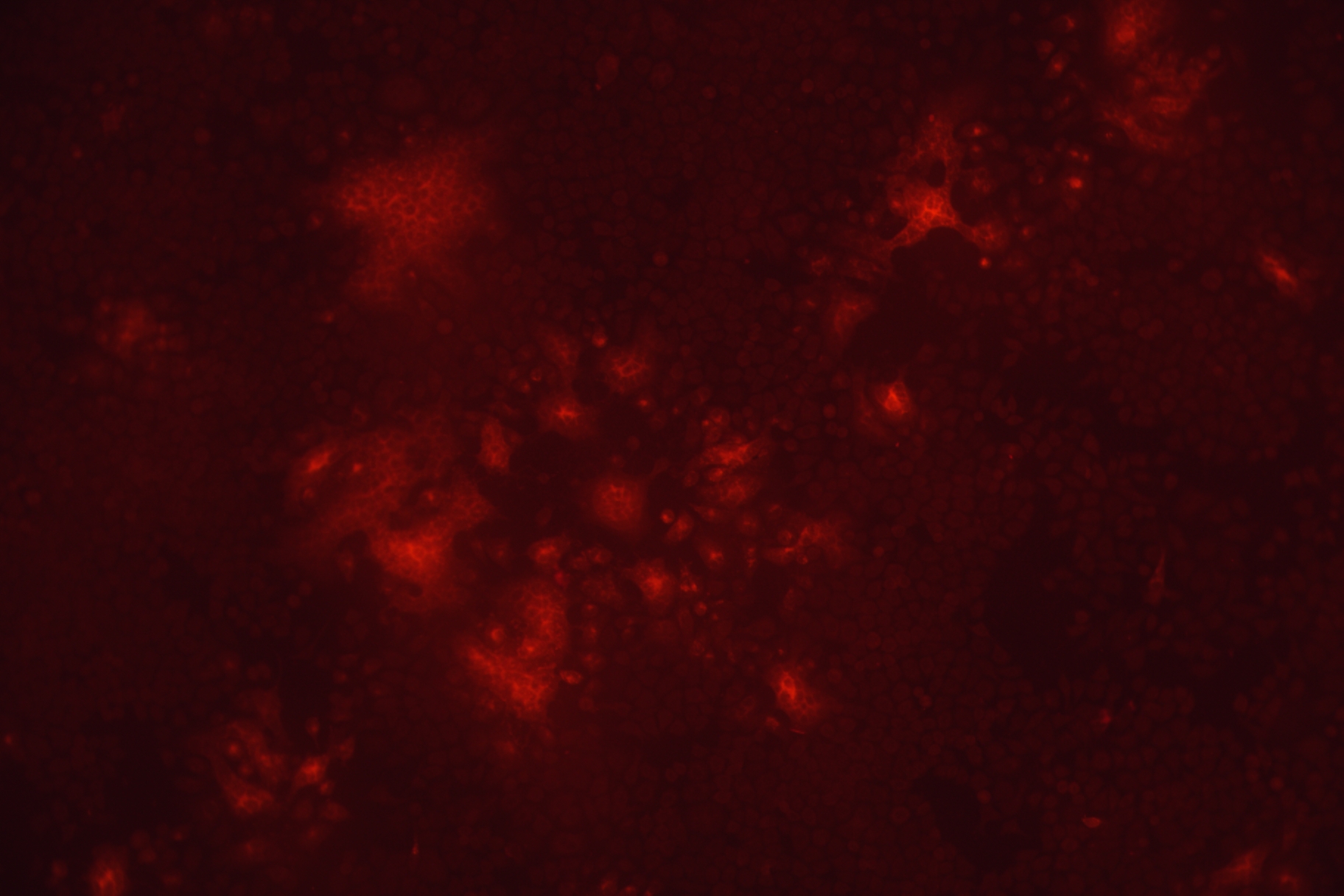

Supplement: S2 File — Level of glycoprotein expression was determined by immunofluorescence assay for the group of IRF1 and its control (pcDNA3), and the group of sh-IRF1 and its control (pSilencer). (ZIP) [file pone.0265925.s001.zip › Repeated experiment Fig 3F_pone.0114021/sh-IRF1/shIRF1-3-HSV1 glycoprotein.jpg]
